# Correction to: A genome-wide association study identified 10 novel genomic loci associated with intrinsic capacity

**DOI:** 10.1093/gerona/glag092

**Published:** 2026-04-29

This is a correction to: Melkamu B. Beyene, Renuka Visvanathan, Robel Alemu, Beben Benyamin, Rudrarup Bhattacharjee, Habtamu B. Beyene, Olga Theou, Matteo Cesari, John R. Beard, Azmeraw T. Amare, A genome-wide association study identified 10 novel genomic loci associated with intrinsic capacity, *The Journals of Gerontology: Series A*, Volume 80, Issue 11, November 2025, glaf196, https://doi.org/10.1093/gerona/glaf196

A second file with supplementary material was erroneously missed at the original publication of the article. This has now been supplied to the article within a zip folder: **Supplementary material.pdf**.

