# Supplementary material for: A genome-wide association study identified 10 novel genomic loci associated with intrinsic capacity
Source: J Gerontol A Biol Sci Med Sci. 2025 Oct 9;80(11):glaf196. doi: 10.1093/gerona/glaf196 (PMC12510315; doi:10.1093/gerona/glaf196)
Supplement: glaf196_Supplementary_Data [file glaf196_supplementary_data.zip › Supplementary material.pdf]

Supplementary Material

Table of Contents

Supplementary Material....1

Supplementary Method 1: Intrinsic capacity score in the CLSA 2

Supplementary Method 2: Data management and analysis codes....11

Supplementary Method 3: GWAS summary access link for traits in Genetic Correlation 24

Supplementary Table 1: SNP-based heritability estimates of IC using GCTA-REML in the UKB and CLSA....26

Supplementary Figure 1: Regional plots for ten loci associated with intrinsic capacity, including nearby genes and functional annotations....27

Supplementary Table 2: The ten genomic loci, Meta GWAS, UKB, and CLSA....28

Supplementary Table 3: IC Candidate SNPs reported in GWAS catalog....29

Supplementary Table 4: The 197 mapped genes from the IC candidate SNPs....62

Supplementary Figure 2: Gene-based Manhattan plot....70

Supplementary Figure 3: Dot plot of beta coefficients and 95% confidence intervals for linear regression of IC on PGS deciles, adjusted for covariates....71

Supplementary Figure 4: Lollipop plot for gene set enrichment analysis....72

Supplementary Table 6: Nominally Enriched pathways....73

Supplementary Figure 5: Dot plot of beta coefficients and 95% confidence intervals for linear regression of IC on PGS deciles, adjusted for covariates....76

Supplementary References....77

Supplementary Method 1: Intrinsic capacity score in the CLSA

The Canadian Longitudinal Study on Aging (CLSA) is a significant research initiative aimed at comprehensively examining the aging process and its implications for health and well-being over time. Initiated in 2010, the CLSA represents one of Canada's largest studies on aging, involving a diverse sample of approximately 50,000 individuals aged 45 to 85 years at baseline. Participants are drawn from various regions across Canada, encompassing both urban and rural settings. The study comprises two primary cohorts: a tracking cohort (~20,000 participants) and a comprehensive cohort (~30,000 participants), each of which is longitudinally followed for 20 years. Data collection includes detailed interviews, physical assessments, and biological samples, providing valuable insights into health determinants and aging trajectories among Canadians. Currently, genomic data has been collected and genotyping performed for the comprehensive cohort (n=26,622 individuals), directing our focus to this cohort for genetic analyses[1, 2]. For more detailed information, please refer to the official CLSA website(https://bmjopen.bmj.com/content/12/3/e059021).

For this study, we developed the Intrinsic Capacity (IC) score within the CLSA using a methodology similar to that we employed in the UK Biobank study[3]. Initially, variables analogous to those used in the UK Biobank and commonly employed to assess the five domains of intrinsic capacity were selected. Subsequently, through exploratory analyses, 14 variables demonstrating factor loadings of 0.2 or higher and exhibiting good fit within the bifactor model were tested in confirmatory models. Thus, a model comprising these 14 variables was utilized to generate IC scores in the CLSA study. The steps undertaken, along with results from exploratory and confirmatory factor analyses and preliminary tests of adequacy, are detailed herein.

Table 1: Description of the items used to measure IC

Variable	Name in the CLSA	Measurement
**Immediate Recall**	COG_REYI_SCORE_COM	Measured using Rey Auditory Verbal Learning Test. Number of words (or variants) correctly recalled in 90 seconds - Immediate Recall.
**Delayed Recall**	COG_REYII_SCORE_COM	Measured using Rey Auditory Verbal Learning Test. Number of words (or variants) correctly recalled in 90 seconds - Delayed Recall.
**Verbal fluency (FAS-score)**	Sum of: FAS_F_SCORE_COM, FAS_A_SCORE_COM, FAS_S_SCORE_COM	This variable measures verbal fluency using the Controlled Oral Word Association Test (COWAT), specifically summing responses for the letters F, A, and S. Each sum represents the total number of words associated with each letter within a designated time frame.
**Standing Balance**	BAL_BEST_COM	This is measured by the best attained time for the accomplishing standing balance test in seconds (given 60 seconds maximum and sopped beyond this).
**Walking Pace**	WLK_TIME_COM	This is a timed 4-meter Walk Test measured in total time required to complete 4mWalk (in seconds).
**Anxiety disorder**	CCC_ANXI_COM	Self-reported (Yes/No) question
**CESD-10 Score**	DEP_CESD10_COM	Center for Epidemiological Studies Short Depression Scale (CES-D 10) score.
**Mood disorder**	CCC_MOOD_COM	Self-reported (yes/no)
**Hearing rating**	HRG_HRG_COM	Self-rated hearing (rated 1-5)
**Hearing aid use**	HRG_AID_COM	Self-reported use of any hearing aid
**Hearing difficulty with background noise**	HRG_NOIS_COM	Self-reported hearing difficulty in the presence of background noise
**Haemoglobin concentration**	BLD_Hgb_COM	Haemoglobin concentration in grams per decilitre
**Hand grip strength**	GS_EXAM_AVG_COM	Average hand grip strength of left and right hand
**FEV1**	Maximum	Forced expiratory volume in 1 second, maximum of all trails made

Factorability (Factor adequacy) test

Though factor adequacy for items commonly used to measure IC is tested for their factorability in different previous studies, we checked this before proceeding to factor analyses to confirm if it is still maintained for the items used in the CLSA. This is usually the first step in factor analyses to determine how suited the data is for factor analysis. We used three methods: the Kaiser-Meyer-Olkin (KMO) Test (should be at least 0.6; mediocre), Bartlett's test of sphericity (a significant test indicates that factor analysis may be worthwhile for the data set), and the determinant of matrix tests (correlation matrix should be > 0.00001)[4]. According to this, our tests using the 14 variables used to develop the IC construct indicate factorability: the overall KMO =0.65, P-value < 0.001 for Bartlett's test of sphericity, and determinant of matrix score = 0.084.

Determining the number of factors

Following factor adequacy testing, we utilized the parallel analysis method—a precise yet underutilized approach previously employed in our study within the UK Biobank—to determine the optimal number of factors for retention. Using this method, we identified five factors in our analysis. [3, 5, 6].

Exploratory factor analysis (EFA)

We conducted both correlated five-factor (conventional) Exploratory Factor Analysis (EFA) and bifactor EFA. The conventional EFA utilized the "psych" package with the "factanal" function, employing the "ProMax" oblique rotation method. For the bifactor EFA, we selected the "bifactor" rotation method. In both analyses, the maximum likelihood (ML) method was chosen for factor extraction [7]. The correlated factors EFA revealed distinct loading patterns of variables across five factors. Similarly, the bifactor EFA demonstrated clear loading of variables onto the five specific domains as well as the general intrinsic capacity domain. The goodness of fit statistics for the bifactor EFA indicated a good model fit, with a Tucker Lewis

index (TLI) of 0.995, root mean square error of approximation (RMSEA) of 0.012, and root mean square residual (RMSR) of 0.01.

Table 2: Conventional EFA

Variable	Factor1	Factor2	Factor3	Factor4	Factor5
Immediate Recall		0.979			
Delayed Recall		0.724			
Verbal fluency (FAS-score)		0.218			0.169
Standing Balance					0.712
Walking Pace					-0.402
Anxiety disorder				0.483	
CESD-10 Score				-0.416	
Mood disorder				0.673	
Hearing rating			-0.611		
Hearing aid use			0.258		0.108
Hearing difficulty with background noise			0.706		
Haemoglobin concentration	0.538				-0.139
Hand grip strength	0.903				
FEV1	0.715				0.179

Table 3: Bifactor EFA

Variable	General	Factor1	Factor2	Factor3	Factor4	Factor5
Immediate Recall	0.45	0.00	0.01	-0.01	0.81	-0.01
Delayed Recall	0.43	-0.02	-0.01	-0.01	0.62	0.03
Verbal fluency (FAS-score)	0.27	0.05	0.00	-0.03	0.19	-0.09
Standing Balance	0.55	0.33	-0.03	0.03	-0.04	-0.01
Walking Pace	-0.34	-0.25	0.00	-0.05	0.04	0.24
Anxiety disorder	-0.02	0.04	0.02	0.47	-0.01	0.02
CESD-10 Score	-0.03	-0.12	-0.12	-0.42	-0.01	0.20
Mood disorder	-0.05	0.06	0.04	0.67	-0.03	0.03
Hearing rating	-0.22	0.02	-0.57	-0.02	-0.01	0.01
Hearing aid use	0.20	0.04	0.24	-0.02	0.01	0.12
Hearing difficulty with background noise	0.14	-0.04	0.67	0.07	0.00	0.00
Haemoglobin concentration	-0.20	0.47	0.00	0.03	-0.01	-0.02
Hand grip strength	-0.11	0.88	0.00	0.03	0.00	-0.03
FEV1	0.09	0.79	-0.02	0.01	0.01	0.05

Confirmatory factor analysis

We did confirmatory factor analysis using the bifactor approach analogous to the one done in the UK biobank, and using this bifactor model under the SEM framework, we developed a score for intrinsic capacity. The "lavaan" package was used for the CFA, employing the Goemin rotation method[8]. The CFA has then confirmed the structure and dimensionality of the IC observed in the EFA (with the goodness of fit statistics; CFI=0.992, TLI=0.985, RMSEA =0.020, and RMSR=0.011). Below are findings from the bifactor CFA.

Table 4: Factor loadings from confirmatory factor analysis

Variable	General	Factor1	Factor2	Factor3	Factor4	Factor5
Hand grip strength	-0.11	0.88	0.00	0.03	0.00	-0.03
FEV1	0.09	0.79	-0.02	0.01	0.01	0.05
Haemoglobin concentration	-0.20	0.47	0.00	0.03	-0.01	-0.02
Hearing aid use	0.20	0.04	0.24	-0.02	0.01	0.12
Hearing rating	-0.22	0.02	-0.57	-0.02	-0.01	0.01
Hearing difficulty with background noise	0.14	-0.04	0.67	0.07	0.00	0.00
Anxiety disorder	-0.02	0.04	0.02	0.47	-0.01	0.02
CESD-10 Score	-0.03	-0.12	-0.12	-0.42	-0.01	0.20
Mood disorder	-0.05	0.06	0.04	0.67	-0.03	0.03
Verbal fluency (FAS-score)	0.27	0.05	0.00	-0.03	0.19	-0.09
Immediate Recall	0.45	0.00	0.01	-0.01	0.81	-0.01
Delayed Recall	0.43	-0.02	-0.01	-0.01	0.62	0.03
Standing Balance	0.55	0.33	-0.03	0.03	-0.04	-0.01
Walking Pace	-0.34	-0.25	0.00	-0.05	0.04	0.24

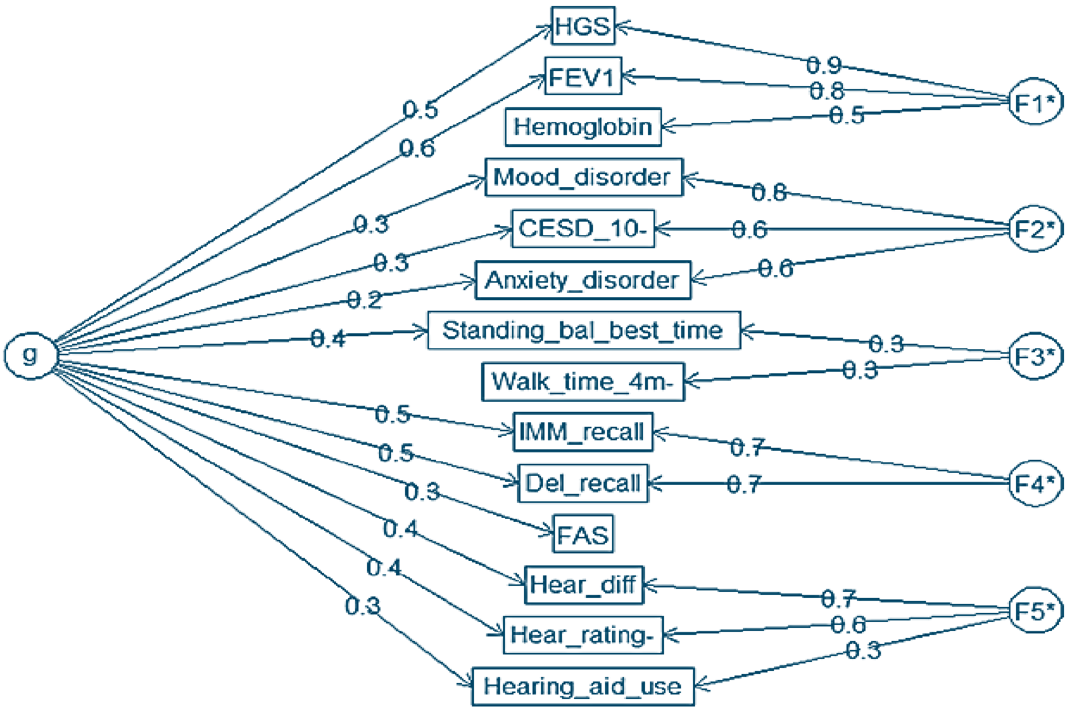

Figure 1. Bifactor CFA model. The g stands for general factor (IC), and F1 to F5 stand for the domains (specific factors) 1-5, respectively.

Distribution of IC score

After generating an intrinsic capacity score for the general and specific domains, we further explored their distributions, patterns of IC with age, and differences in IC score between the two sexes.

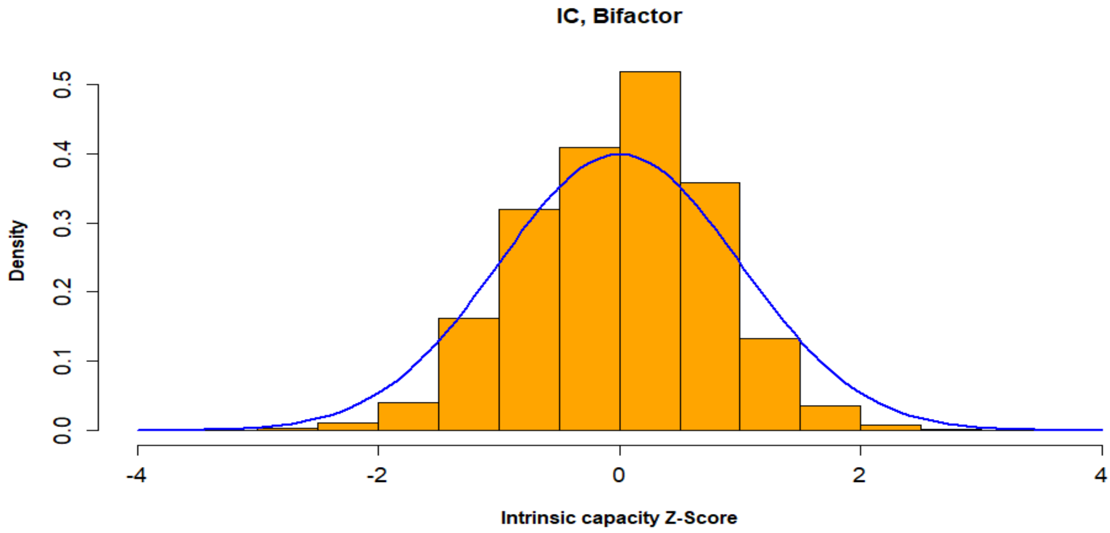

Figure 2: Histogram with Normal Curve for the Intrinsic Capacity General Score. This histogram, overlaid with a normal curve, shows that the intrinsic capacity general score constructed from the bifactor confirmatory model has an approximately normal distribution.

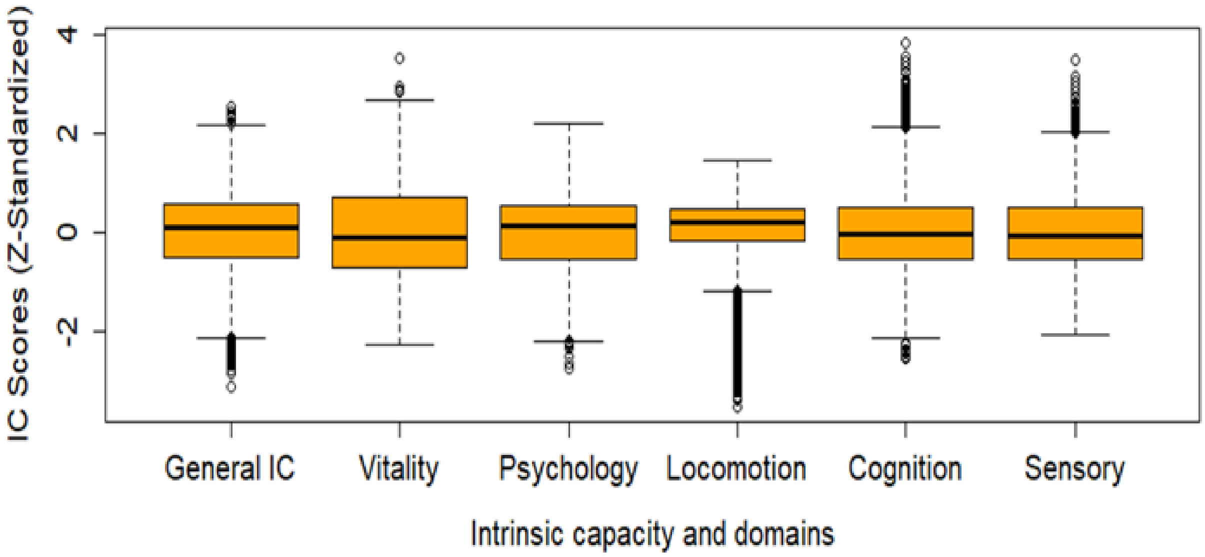

Figure 3: Box plots for the Intrinsic Capacity General Score and Domain-Specific Scores Developed from the Bifactor Confirmatory Model. These box plots illustrate that the distributions of both the general score and domain-specific scores are approximately normal, with no severe outlier values observed.

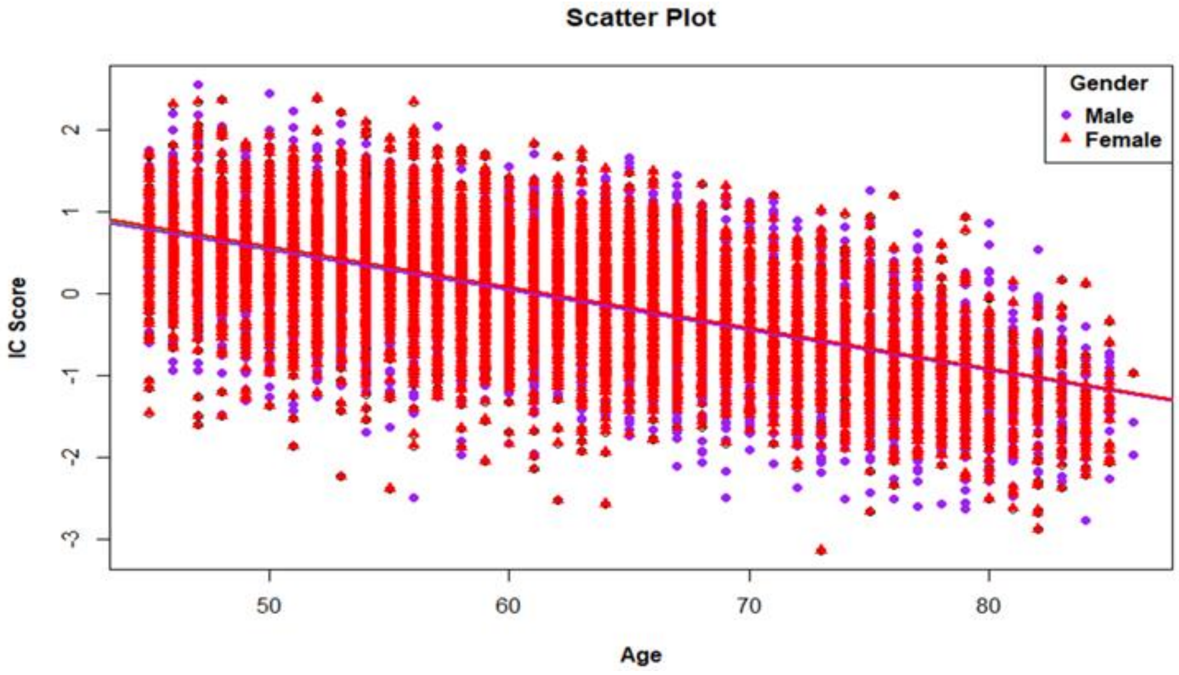

Figure 4: Scatter plot of IC with age with linear regression line for males and females. This figure illustrates a scatter plot with a fitted linear regression line. It demonstrates a consistent decrease in the intrinsic capacity score with increasing age. Furthermore, the distribution of IC scores between males and females exhibits minor differences across age groups.

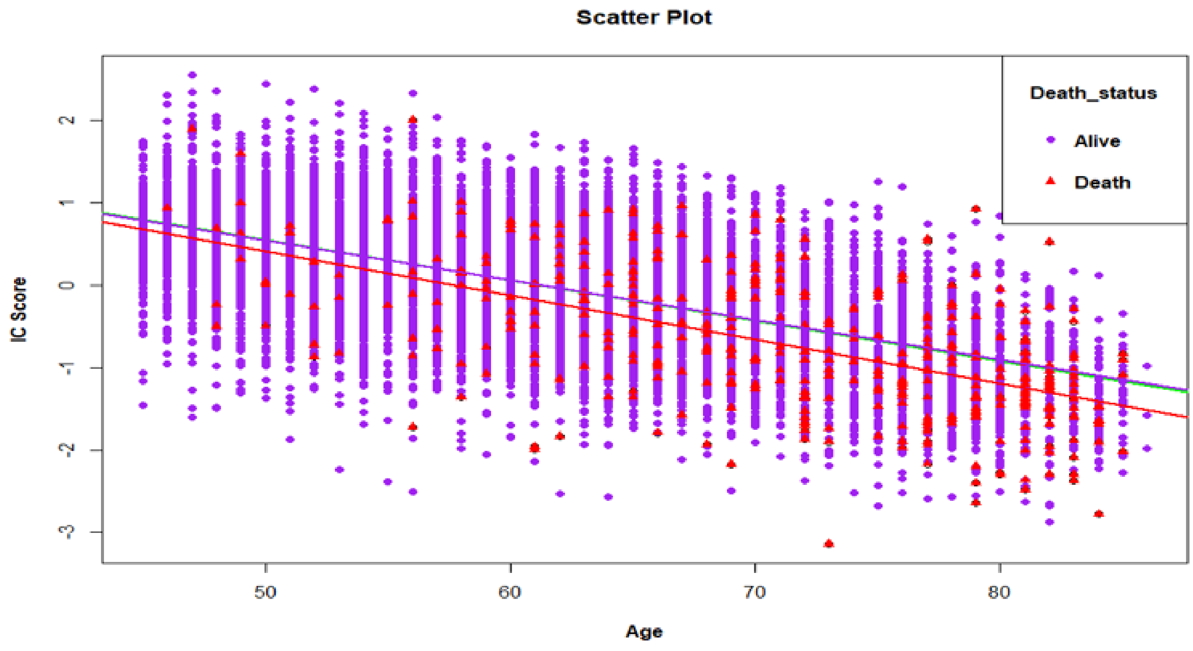

Figure 5: Scatter plot of IC with age, with linear regression lines for deceased and alive. The figure illustrates that, on average, the baseline IC score for the dead is lower than that for the alive.

The logistic regression analysis for exploring if intrinsic capacity predicts death or not showed that the risk of death is higher for people with lower intrinsic capacity. People with a one-unit higher baseline IC Z-score have, on average, about 40% lower risk of death at the end of the second follow-up (OR =0.5951; 95% CI: 0.505-0.701). The death record at the second follow-up (Data released in September 2022) was used for ascertaining death status in this analysis.

Table 5: The distribution of death in the four quartiles of intrinsic capacity. As can be seen from the distribution, more than half (59%) of the deaths are in the first quartile of intrinsic capacity, followed by the next highest death in the second quartile of IC, whereas the third and fourth quartiles have almost the same number of deaths.

		**IC Quartiles**			
		Q1	Q2	Q3	Q4
**Death Status**	Alive	3039	3192	3238	3238
	Dead	239	86	40	40

Table 6: List of all the 27 variables initially selected to operationalise IC

**Cognitive capacity**	**Locomotive capacity**	**Psychological capacity**	**Sensory capacity**	**Vitality capacity**
Immediate recall	Average hrs of walking per day	Anxiety disorder (yes/no)	Hearing rating (self-rating)	Haemoglobin concentration
Delayed recall	Frequency of moderate physical activity	CESD10 depression score	Hearing aid use (Yes/No)	Grip strength (average)
Reaction time	Standing Balance	Mood disorder (yes/No)	Hearing difficulty	Appetite

			with background noise	
Mental alteration test (MAT score)	Gait speed (time-spent_4m-walk)	Average hours of sleep per day (last month)	Visual acuity (best eye)	Weight loss
Animal fluency test (AFT_score1)	Frequency of taking a walk out of home		Vision aid use	Forced expiratory volume in 1 second (FEV1)
Verbal fluency test (FAS total score)	Chair rise test			
	timed Up and Go test (TUG test)			

Table 7: Independent samples t-test comparing mean IC scores between the UKB and CLSA cohorts

Cohort	Mean IC score	SD	t	Df	P-Value
**UKB**	0.000014	0.64	0.002	58318	0.9983
**CLSA**	0.000000012	0.77			

Supplementary Method 2: Data management and analysis codes

```
################# Genetic Data Quality Control ################################
################### A. QC UKB ##############################################
### SNP QC
#Step-1: Extract the SNPS from the main bgen file (infoscore >=0.3; infor score field ID in UK biobank:100319)
wget  -nd  biobank.ndph.ox.ac.uk/ukb/ukb/auxdata/ukb_imp_mfi.tgz
tar -xvzf ukb_imp_mfi.tgz

#Then go to R on phoenix and from the extracted info score for each of the 22 chromosomes, prepare a text file that conatins only those SNPS with info score of 0.3.

#Read file on R (call)
a=read.table("ukb_mfi_chr22_v3.txt", header=F, sep='\t')

# extract only those with info score>=0.3
chr22_info=subset(a, V8>=0.3)

#slect only SNP and info score columns
chr22_info=chr22_info[,c(2,8)]
```

#Finally, on R, prepare a file containg SNPS and info score to take to phoenix for extraction genotype data from the main bgen files.
write.table(chr22_info, 'SNP_list_info_chr22.txt', row.names=F, col.names=F, quote=F, sep='\t')

#Then quit R and go back to phoenix
q()

#Step-2: Extract the bgen file using the the list of SNPs prepared by screening using info score >=0.3 for each chromosome.

Using Combined SNP list for chromosomes 1-22, extract bgen file from each chromosome as:

```
for chr in {1..22}; do
  ./plink2 --threads 8 \
    --bgen ukb22828_c${chr}_b0_v3.bgen ref-first \
    --sample ukb22828_c22_b0_v3_s487160.sample \
    --extract SNP_list_info_chr1_chr22.txt \
    --set-missing-var-ids @:#[b37]\$r,\$a \
    --export bgen-1.2 \
    --out ukb_chr${chr}_post_impute
done
```

#Step-3: extract using the maf 0.01, geno 0.05, mind 0.05, hwe 1e-7 and max-alleles 2.

```
for chr in {1..22}; do
  ./plink2 --threads 6 \
    --bgen ukb_chr${chr}_post_impute.bgen ref-first \
    --sample ukb_chr${chr}_post_impute.sample \
    --maf 0.01 \
    --geno 0.05 \
    --mind 0.05 \
    --hwe 1e-7 \
    --max-alleles 2 \
    --export bgen-1.2 \
    --out ukb_chr${chr}_post_impute_1
done
```

#Step_4: make ".bed" files from the .bgen files

```
for chr in {1..22}; do
  # First command
  ./plink2 --bgen ukb_chr${chr}_post_impute_1.bgen ref-first \
    --sample ukb_chr${chr}_post_impute_1.sample \
    --rm-dup \
    --make-bed \
    --out ukb_chr${chr}_post_impute_2

  # Second command
  ./plink2 --bgen ukb_chr${chr}_post_impute_1.bgen ref-first \
```

```
    --sample ukb_chr${chr}_post_impute_1.sample \
    --exclude ukb_chr${chr}_post_impute_2.rmdup.mismatch \
    --make-bed \
    --out ukb_chr${chr}_post_impute_2
done
```

############### Individuaul (sample) QC

Step-5: exclude those individuals with sex aneuploidy ==1 (use UKB field "sex_aneuoploidy" and prepared ID list of individulas with sex aneuploidy value =1 to "sex_aneuploidy.txt" screen out those individuals.

```
for (( i=1; i<=22; i++ )); do
    ./plink2 --bfile ukb_chr"${i}"_post_impute_2 --remove sex_aneuploidy.txt --make-bed --out ukb_chr"${i}"_post_impute_3
done
```

#Step-6: Sex-Mismatch (exclude sex matched individulas comaring genetic sex and self reported sex).

```
for (( i=1; i<=22; i++ )); do
    ./plink2 --bfile ukb_chr"${i}"_post_impute_3 --remove sex_mismatch_m.txt --make-bed --out ukb_chr"${i}"_post_impute_4
done
```

#Step-7: Hetrozygosity.

```
for (( i=1; i<=22; i++ )); do
    ./plink2 --bfile ukb_chr"${i}"_post_impute_4 --het --out R_check_"${i}"
done
```

#Then go to R on linux

```
het <- read.delim("R_check.het", head=TRUE)
pdf("heterozygosity.pdf")

het$HET_RATE = (het$"E.HOM." - het$"O.HOM.")/het$"E.HOM."

het$HET_RATE=(het$X51077-het$X49388.8)/het$X51077 [I used this for my UKB data instead of the above. It gives me HET_RATE =1- O(HOM)/E(HOM)

hist(het$HET_RATE, xlab="Heterozygosity Rate", ylab="Frequency", main= "Heterozygosity Rate")
dev.off()


het_fail = subset(het, (het$HET_RATE < mean(het$HET_RATE)-3*sd(het$HET_RATE)) | (het$HET_RATE > mean(het$HET_RATE)+3*sd(het$HET_RATE)));
het_fail$HET_DST = (het_fail$HET_RATE-mean(het$HET_RATE))/sd(het$HET_RATE);
```

write.table(het_fail, "fail-het-qc.txt", row.names=FALSE) [have to adjust "fail_het_qc_#" for chr you are doing]

#Then back to phoenix and filter out those indivduals with heterozygosity out of the range (using the text file prepared in R above).

```
for (( i=1; i<=22; i++ )); do
   sed 's/"//g' fail-het-qc_"${i}".txt | awk '{print $1, $2}' > het_fail_ind_"${i}".txt
done
```

```
#Then;
for ((i=1; i<=22; i++)); do
   ./plink2 --bfile ukb_chr"${i}"_post_impute_4 --remove het_fail_ind_"${i}".txt --make-bed
--out ukb_chr"${i}"_post_impute_5
done
```

Step-8: Merge the final binary file for Analysis

```
./plink1.9 --bfile ukb_chr1_post_impute_5 --threads 72 --merge-list all_files_4merging.txt --
make-bed --out merged_data
```

WE THEN USED THIS FINAL "mereged_data" FOR GENETIC ANALYSES!

########################## B. QC CLSA ##########################
SNP QC

#Step1. Prepare list of SNIPs with info score >=0.3 (two columns; SNPs ID and infoscore) and save to file name "clsa_info_score_0.3.txt " ["can do this in R"]

#Step2. Extract bgen by the info score file (clsa_info_score_0.3.txt)created.

```
for chrom in {1..22}; do
  ./plink2 --threads 8 \
    --bgen clsa_imp_${chrom}_v3.bgen ref-first \
    --sample clsa_imp_v3.sample \
    --extract clsa_info_score_0.3.txt \
    --export bgen-1.2 \
    --out clsa_imp_${chrom}_v3_post_impute
done
```

#Step3. Apply SNP based QC filtering

```
for chr in {1..22}; do
  ./plink2 --threads 6 \
```

```
    --bgen clsa_imp_${chr}_v3_post_impute.bgen ref-first \
    --sample clsa_imp_${chr}_v3_post_impute.sample \
    --maf 0.01 \
    --geno 0.05 \
    --mind 0.05 \
    --hwe 1e-7 \
    --max-alleles 2 \
    --export bgen-1.2 \
    --out clsa_imp_${chr}_v3_post_impute_1
done

#Step4. Idenitfy and Remove duplicates and make bed files

for chr in {1..22}; do
  ./plink2 --bgen clsa_imp_${chr}_v3_post_impute_1.bgen ref-first \
    --sample clsa_imp_${chr}_v3_post_impute_1.sample \
    --rm-dup \
    --make-bed \
    --out clsa_imp_${chr}_v3_post_impute_2
done

# Step5. Clean using the duplicate and mismatch file created
for chr in {1..22}; do
  ./plink2 --bgen clsa_imp_${chr}_v3_post_impute_1.bgen ref-first \
    --sample clsa_imp_${chr}_v3_post_impute_1.sample \
    --exclude clsa_imp_${chr}_v3_post_impute_2.rmdup.mismatch \
    --make-bed \
    --out clsa_imp_${chr}_v3_post_impute_2
done

#Step6. Merege the bed files (chr 1-22)
./plink2 \
  --bfile clsa_imp_1_v3_post_impute_2 \
  --threads 12 \
  --merge-list all_files_4merging.txt \
  --make-bed \
  --out clsa_SNP_QC_merged_data


### Individual QC (on the merged bed file)
#Step7.Sex Chromosome anneuploid (remove anneuploid ones)
./plink2 \
  --bfile clsa_SNP_QC_merged_data \
  --remove Sex_chromosome_anneploidy.tab \
  --make-bed \
  --out clsa_SNP_QC_merged_data_1

#Step8. Exclude those with sex mismatch between reported and chromosomal sex
./plink2 \
  --bfile clsa_SNP_QC_merged_data_1 \
```

```
  --remove Sex_mismatch_ids.tab \
  --make-bed \
  --out clsa_SNP_QC_merged_data_2

#Step9. Hetrozygosity
 ./plink2 \
  --bfile clsa_SNP_QC_merged_data_2 \
  --remove Hetrozygosity.tab \
  --make-bed \
  --out clsa_SNP_QC_merged_data_3
```

WE THEN USED THIS FINAL "clsa_SNP_QC_merged_data_3" FOR GENETIC ANALYSES!

#################### C. Heritability UKB ##########################

First prepare files and upload - IC score file, qualitative covariates and quantiative covaraites in separte files (quantitaive covaraties including the ten PCs convereted to z score).
Produce the Genetic relationship matrix (GRM)

GRM (submit job using #!/bin/bash)

```
./gcta-1.94.1 \
  --bfile merged_chr1-22_45K \
  --autosome \
  --make-grm \
  --out GRM_GCTA_UKB_chr1_22_45Ksamples
```

Heritability analysis (sub mit job using #!/bin/bash)

```
./gcta-1.94.1 \
  --grm GRM_GCTA_UKB_All_45Ksamples/GRM_GCTA_UKB_chr1_22_45Ksamples \
  --pheno IC_bifactor_score.tab \
  --reml \
  --covar Quali_cov.tab \
  --qcovar Quanti_cov_z_pc10.tab \
  --out
Heritability_GCTA_UKB_IC_All_45Ksamples/Heritability_estimate_GCTA_UKB_All_45K
samples \
  --thread-num 10
```

##################### D. Heritability CLSA ####################

Heritability in CLSA - exactly same steps followed as in UKB
First prepare files and upload - IC score file for CLSA, qualitative covariates and quantiative covaraites in separte files (quantitaive covaraties including the ten PCs convereted to z score).
Produce the Genetic relationship matrix (GRM)

```
### GRM (linux code: submit job using #!/bin/bash)

./gcta-1.94.1 \
  --bfile CLSA/CLSA_BGEN_CHR1-22/clsa_SNP_QC_merged_data_3 \
  --autosome \
  --make-grm \
  --out GRM_GCTA_CLSA_All_26KSamples


### Heritability CLSA

./gcta-1.94.1 \
  --grm GRM_GCTA_CLSA_All_26KSamples \
  --pheno IC_only_bifactor_score_CLSA.txt \
  --reml \
  --covar Quali_Cov.tab \
  --qcovar Quanti_cov_Zscored_10PCs.tab \
  --out Heritability_estimate_GCTA_CLSA_All_26Ksamples \
  --thread-num 10

################# E. GWAS UKB ###############################################

### for fastGWA-mlm create sparse GRM from dense GRM produced above
./gcta-1.94.1 \
  --grm GRM_GCTA_UKB_chr1_22_45Ksamples \
  --make-bK-sparse 0.05 \
  --out sparse_grm_output_gcta_all_45Ksamles

### GWAS UKB (fastGWA-mlm)
./gcta-1.94.1 \
  --fastGWA-mlm \
  --bfile merged_chr1-22_45K \
  --grm-sparse sparse_grm_output_all_45Ksamples \
  --pheno IC_bifactor_score.tab \
  --covar Quali_cov.tab \
  --qcovar Quanti_cov_z_pc10.tab \
  --out GWAS_Summary_UKB_IC_GCTA_fastGWA-mlm_All_45ksamples \
  --thread-num 11

################################# F. GWAS CLSA ################################

### Sparse GRM
./gcta-1.94.1 \
  --grm GRM_GCTA_CLSA_All_26KSamples \
  --make-bK-sparse 0.05 \
  --out sparse_grm_gcta_clsa_26Ksamles

### GWAS CLSA (fastGWA-mlm)
./gcta-1.94.1 \
```

```
--fastGWA-mlm \
--bfile CLSA/CLSA_BGEN_CHR1-22/clsa_SNP_QC_merged_data_3 \
--grm-sparse sparse_grm_gcta_clsa_26Ksamles \
--pheno IC_only_bifactor_score_CLSA.txt \
--covar Quali_Cov.tab \
--qcovar Quanti_cov_Zscored_10PCs.tab \
--out GWAS_Summary_CLSA_IC_GCTA_fastGWA-mlm_All_26ksamples \
--thread-num 11
```

####################### G. Meta GWAS - Metal ############################

We used metal package (check the github/documentation at: https://genome.sph.umich.edu/wiki/METAL_Documentation)

We prepare the Metal script first and saved it as "metal_SE_weighted_GRCH37.txt" (can be any other name)
Upload the the summary statistics to be meta anlysed (need to be formated same) - for this anlaysis - UKB and CLSA summary statistics
the Metal scirpt has paramters to set (read the documentation).For this anlysis we have set parameters as below;

code in our script file metal_SE_weighted_GRCH37.txt (our gwas summarries are: "GWAS_UKB_GRCH37.tab" and "GWAS_CLSA_GRCH37.tab").

```
#SCHEME STDERR
#SEPARATOR  TAB
#WEIGHT N
#WEIGHTLABEL N
#MARKER SNP
#ALLELE A1 A2
#EFFECT BETA
#STDERR SE
#STDERRLABEL SE
#PVALUE P
#PROCESS GWAS_UKB_GRCH37.tab
#PROCESS GWAS_CLSA_GRCH37.tab
#ANALYZE
#ANALYZE HETEROGENEITY
#QUIT
```

Then saving the script file and going back to linux we run the metal script
./metal metal_SE_weighted_GRCH37.txt

Additional by swapping SCHEME to SAMPLESIZE, we have done run the metal analysis to get N (it is not found in the STDERR option)

```
#SCHEME SAMPLESIZE
#SEPARATOR  TAB
#WEIGHT N
```

#WEIGHTLABEL N
#MARKER SNP
#ALLELE A1 A2
#EFFECT BETA
#STDERR SE
#STDERRLABEL SE
#PVALUE P
#PROCESS GWAS_UKB_GRCH37.tab
#PROCESS GWAS_CLSA_GRCH37.tab
#ANALYZE
#ANALYZE HETEROGENEITY
#QUIT

Then saving the script file and going back to linux we run the metal script
./metal metal_n_weighted_GRCH37.txt

################### H. Polygenic score analysis #################################
github_link: https://github.com/getian107/PRScs

Up loaded files
GWAS summary for UKB (columns required and naming - format to: CHR, SNP, A1, A2, BETA, P)
LD reference panel - EUR reference (download and untar from the github page)
Path to final QCed renamed clsa binary file ("renamed_new_filtered_clsa_SNP_QC_merged_data_3")

#1.Install PRS-CS. Available from GitHub

git clone https://github.com/getian107/PRScs.git

cd PRScs

#2. Create pynthon environement its dependcies - PRS-CS requires a specific Python environment.
module load python #add python version (copy from available in linux)
module load Anaconda #add anaconda version (copy from available in linux)

conda create -n prscs_env python=3.7

conda activate prscs_env

pip install h5py

pip install numpy scipy pandas matplotlib scikit-learn

#3. Post-estimation analysis PRScs (Can be in job submission format too)

```
ppython PRScs.py \
  --ref_dir=${base_dir}/ldblk_1kg_eur \
  --bim_prefix=${base_dir}/renamed_new_filtered_clsa_SNP_QC_merged_data_3 \
  --sst_file=${base_dir}/GWAS_UKB_GRCH38_Final_PB_new.tab \
  --n_gwas=44631 \
  --phi=1e-2 \
  --chr=1,2,3,4,5,6,7,8,9,10,11,12,13,14,15,16,17,18,19,20,21,22 \
  --out_dir=/hpcfs/users/a1877115/PRS/PRScs/UKB_PRS_post_eff_IC

#4. Concatenate the post estimate effect sizes for the 22 chromosomes
cat UKB_PRS_post_eff_IC_pst_eff_a1_b0.5_phi1e-02_chr{1..22}.txt > UKB_PRS_post_eff_IC_pst_eff_a1_b0.5_phi1e-02_all.txt

#5. Developing PRS (first remove duplicate and makebed - renamed_new_filtered_clsa_SNP_QC_merged_data_4 and then proceed to PRS scores creation)
# Remove duplicates
./plink2 \
  --bfile ${base_dir}/renamed_new_filtered_clsa_SNP_QC_merged_data_3 \
  --rm-dup force-first \
  --make-bed \
  --out ${base_dir}/renamed_new_filtered_clsa_SNP_QC_merged_data_4
#Score
./plink2 \
  --bfile ${base_dir}/renamed_new_filtered_clsa_SNP_QC_merged_data_4 \
  --score ${base_dir}/UKB_PRS_post_eff_IC_pst_eff_a1_b0.5_phi1e-02_all.txt 2 4 6 \
  --out ${base_dir}/PRScs_Scores/simplex


### PGS Association testing (linear regression using R)
#Go to R software and prepare mereged data with PRS scores and all varaibles required for regression analysis (All_vars_PRScs_CLSA.tab)
#calculate R2 using full covaraites including PRS
#calculate R2 using covaraites except PRS
#calculate the difference in R2 which tell you how much varaibaility the PRS explains

### Load data to R
All_vars_PRScs<-
read.table("//uofa/users$/users5/a1877115/Desktop/PRS/All_vars_PRScs_CLSA.tab", header = TRUE)

# Models
model1 <- lm(IC_Zscore ~ Age + PC1 + PC2 + PC3 + PC4 + PC5 + PC6 + PC7 + PC8 + PC9 + PC10 + as.factor(Sex) + as.factor(Batch) + PRScs_Zscore, data = All_vars_PRScs)
model2 <- lm(IC_Zscore ~ Age + PC1 + PC2 + PC3 + PC4 + PC5 + PC6 + PC7 + PC8 + PC9 + PC10 + as.factor(Sex) + as.factor(Batch), data = All_vars_PRScs)

summary(model1)
conf_intervals <- confint(model1, level = 0.95)
```

```
print(conf_intervals)

summary(model2)


### Association tetsing using PGS deciles
All_vars_PRScs$PRS_deciles <- cut(All_vars_PRScs$PRScs_Zscore, breaks = quantile(All_vars_PRScs$PRScs_Zscore, probs = 0:10 / 10), labels = FALSE, include.lowest = TRUE)

model_lm_deciles <- lm(IC_Zscore ~ Age + PC1 + PC2 + PC3 + PC4 + PC5 + PC6 + PC7 + PC8 + PC9 + PC10 + as.factor(Sex) + as.factor(Batch) + as.factor(PRS_deciles), data = All_vars_PRScs)
conf_intervals <- confint(model_lm_deciles, level = 0.95)


####################### I. Genetic correlation analysis #########################

### github link to do LDSC genetic correlation analysis
Github: https://github.com/bulik/ldsc

### Instal LDSC
git clone https://github.com/bulik/ldsc.git
cd ldsc

### Instal Python and Anaconda softwares
module load Python/3.11.3-GCCcore-12.3.0
module load Anaconda3/2024.06-1

### Create Python environment
conda env create --file environment.yml
source activate ldsc

### Download Reference LD Scores (European)
wget -r -np -nH --cut-dirs=3 -R index.html https://ibg.colorado.edu/cdrom2021/Day06-nivard/GenomicSEM_practical/eur_w_ld_chr/
vtar -xvjf eur_w_ld_chr.tar.bz2


### Prepare GWAS Summary Statistics for Trait 1 (IC)
#!/bin/bash

# Directory containing the munged summary statistics files
trait_dir="./GWAS_Summary_statistics for genetic correlation"

# Path to the already munged Intrinsic Capacity (IC) summary statistics
ic_sumstats="${trait_dir}/Intrinsic_Capacity_gwas.sumstats.gz"

# Check if the IC summary statistics file exists
if [ ! -f "$ic_sumstats" ]; then
```

```
  echo "Intrinsic Capacity summary statistics file not found: ${ic_sumstats}"
  exit 1
fi

# Loop through each munged summary statistics file in the folder
for file in "${trait_dir}"/*_gwas.sumstats.gz; do
  # Extract the trait name from the file name
  trait=$(basename "${file}" _gwas.sumstats.gz)

  echo "Calculating genetic correlation for trait: ${trait}"

  # Calculate genetic correlation between IC and each trait
  python2 ldsc.py \
  --rg "${ic_sumstats},${file}" \
  --ref-ld-chr eur_w_ld_chr/ \
  --w-ld-chr eur_w_ld_chr/ \
  --out "${trait_dir}/IC_${trait}"

  # Check if the genetic correlation calculation was successful
  if [ $? -ne 0 ]; then
    echo "Error calculating genetic correlation for trait: ${trait}"
  fi
done

############ Loop menging through each summary statistics file in the folder

#!/bin/bash

# Directory containing the trait summary statistics files
trait_dir="./GWAS_Summary_statistics for genetic correlation"

# List the files in the directory for debugging
echo "Listing files in ${trait_dir}:"
ls -l "${trait_dir}"

# Loop through each summary statistics file in the folder
for file in "${trait_dir}"/*.tab; do
  # Check if the file exists
  if [ ! -f "$file" ]; then
    echo "No .tab files found in the directory."
    continue
  fi

  # Extract the trait name from the file name
  trait=$(basename "${file}" .tab)

  echo "Munging summary statistics for trait: ${trait}"

  # Munge summary statistics for each trait
  ./munge_sumstats.py \
```

```
  --out "${trait_dir}/${trait}_gwas" \
  --merge-alleles w_hm3.snplist \
  --N-col N \
  --sumstats "${file}" \
  --a1 A1 \
  --a2 A2 \
  --snp SNP \
  --p P \
  --signed-sumstats BETA,0 \
  --chunksize 50000

  # Check if the munging was successful
  if [ $? -ne 0 ]; then
    echo "Error munging summary statistics for trait: ${trait}"
    continue
  fi
done

### loop GC analysis through each trait with IC

#!/bin/bash

# Directory containing the munged summary statistics files
trait_dir="./GWAS_Summary_statistics for genetic correlation"

# Loop through each munged summary statistics file in the folder
for file in "${trait_dir}"/*_gwas.sumstats.gz; do
  # Extract the trait name from the file name
  trait=$(basename "${file}" _gwas.sumstats.gz)

  echo "Calculating genetic correlation for trait: ${trait}"

  # Calculate genetic correlation between IC and each trait
  python2 ldsc.py \
  --rg "${trait_dir}/Intrinsic_Capacity_gwas.sumstats.gz,${file}" \
  --ref-ld-chr eur_w_ld_chr/ \
  --w-ld-chr eur_w_ld_chr/ \
  --out "${trait_dir}/IC_${trait}"

  # Check if the genetic correlation calculation was successful
  if [ $? -ne 0 ]; then
    echo "Error calculating genetic correlation for trait: ${trait}"
  fi
done

############################## DONE #######################################
```

Supplementary Method 3: GWAS summary access link for traits in Genetic Correlation

No	trait	year	Ancestery	N	Study access link
**1**	Low_grip_strength	2021	European	256,523	https://pubmed.ncbi.nlm.nih.gov/33510174/
**2**	FEV1	2023	European	475,645	https://www.nature.com/articles/s41588-023-01314-0
**3**	Hemoglobin_concentration (UKB)	2024	European	174,488	https://www.nature.com/articles/s42003-024-05874-7
**4**	BMI	2024	European	650,000	https://www.nature.com/articles/s41588-024-01940-2
**5**	Hip_circumference_BMIadjusted	2021	European	219,872	https://www.nature.com/articles/s41598-021-89176-6
**6**	Body_fat_mass	2024	European	337,196	https://www.nature.com/articles/s41598-024-54291-7
**7**	FVC	2024	European	255,647	https://pubmed.ncbi.nlm.nih.gov/38165527/
**8**	ADHD	2023	European	225,534	https://pubmed.ncbi.nlm.nih.gov/36702997/
**9**	Alzheimer's_disease	2022	European	788,989	https://www.nature.com/articles/s41588-022-01024-z
**10**	Anxiety_panic_disorder	2019	European	10,240	https://www.nature.com/articles/s41380-019-0590-2
**11**	Depression_MDD	2019	European	807,553	https://www.nature.com/articles/s41593-018-0326-7
**12**	Bipolar	2021	European	413,466	https://www.nature.com/articles/s41588-021-00857-4
**13**	Schizophrenia	2018	European and east Asian	185,864	https://pubmed.ncbi.nlm.nih.gov/29483656/
**14**	Alcohol_use_disorder	2024	European	118,022	https://www.nature.com/articles/s41562-024-01909-5
**15**	Neuroticism	2024	European	174,488	https://pubmed.ncbi.nlm.nih.gov/38351177/
**16**	Reasoning_verbal_numerical	2016	European	36,035	https://www.nature.com/articles/mp201645
**17**	Intelligence	2018	European	269,867	https://www.nature.com/articles/s41588-018-0152-6
**18**	Reaction_time	2018	European	300,486	https://www.nature.com/articles/s41467-018-04362-x
**19**	Executive_function	2022	European	427,037	https://www.sciencedirect.com/science/article/pii/S0006322322014056?via%3Dihub
**20**	Memory_performance	2016	European	112, 067	https://www.nature.com/articles/mp201645
**21**	Prospective_memory	2022	European	427,037	https://www.sciencedirect.com/science/article/pii/S0006322322014056?via%3Dihub
**22**	Eudcational_attainment	2024	European (766,345) and East Asian (165,232)	931,577	https://www.nature.com/articles/s41562-023-01781-9
**23**	Frailty_index	2021	European	175,226	https://pubmed.ncbi.nlm.nih.gov/34431594/
**24**	Duration_mod_int_PA	2022	European	88411	https://pubmed.ncbi.nlm.nih.gov/35043453/
**25**	Walking_pace_slow	2021	European	428255	https://pubmed.ncbi.nlm.nih.gov/34662886/
**26**	Duration_of_walks	2021	European	372,854	https://pubmed.ncbi.nlm.nih.gov/34662886/
**27**	Fat_free_mass	2024	European	337739	https://pubmed.ncbi.nlm.nih.gov/38538606/

**28**	Bone_density	2024	European	115532	https://pubmed.ncbi.nlm.nih.gov/38965376/
**29**	Hearing_difficulty_background_noise	2021	European	447071	https://pubmed.ncbi.nlm.nih.gov/34737426/
**30**	Visual_impairment (progressive visual loss)	2024	European	80058	https://pubmed.ncbi.nlm.nih.gov/38965376/
**31**	Retinal_detachment	2019	European	364210	https://pubmed.ncbi.nlm.nih.gov/31816047/
**32**	Age-related hearing impairment	2020	European	330759	https://pubmed.ncbi.nlm.nih.gov/32986727/
**33**	Cardiometabolic_multimorbidity	2024	European	367,147	https://pubmed.ncbi.nlm.nih.gov/38409652/
**34**	Parkinson_disease	2023	Multi-ancestery (578,413 European; 31,575 east asian and 1497 Hispanic)	611,485	https://www.nature.com/articles/s41588-023-01584-8
**35**	Cancer (UKB data field 2453)	2021	European	454,736	https://pubmed.ncbi.nlm.nih.gov/34737426/
**36**	Multiple_system_atrophy	2024	European	8016	https://pubmed.ncbi.nlm.nih.gov/38701790/
**37**	Dementias	2018	European	403339	https://pubmed.ncbi.nlm.nih.gov/30104761/
**38**	Systolic_BP	2024	European	1028980	https://pubmed.ncbi.nlm.nih.gov/38689001/
**39**	Dystolic_BP	2024	European	1028980	https://pubmed.ncbi.nlm.nih.gov/38689001/
**40**	Essential_Hypertension	2021	European	331754	https://pubmed.ncbi.nlm.nih.gov/34662886/
**41**	Total_lipid_LDL	2024	European	93799	https://pubmed.ncbi.nlm.nih.gov/39278973/
**42**	Total_lipid_HDL	2024	European	93799	https://pubmed.ncbi.nlm.nih.gov/39278973/
**43**	fasting_glucose	2023	Mixed (10177 African, 651 East Asian, 113279 European, 3153 Hispanic, 2405 South Asian	129665	https://pubmed.ncbi.nlm.nih.gov/39280063/
**44**	Telomere_length	2022	European	902	https://pubmed.ncbi.nlm.nih.gov/35681050/
**45**	fasting_insulin	2023	Mixed (8690 African, 652 East Asian, 91501 European, 1142 Hispanic, 2155 South Asian	104140	https://pubmed.ncbi.nlm.nih.gov/39280063/
**46**	Heart_rate_variability	2023	European	46075	https://pubmed.ncbi.nlm.nih.gov/37803156/
**47**	Asthma	2023	European	381782	https://pubmed.ncbi.nlm.nih.gov/37377600/
**48**	CRP_levels (UKB)	2024	European	174488	https://pubmed.ncbi.nlm.nih.gov/38351177/
**49**	Chronic_pain	2024	European	360249	https://pubmed.ncbi.nlm.nih.gov/38965376/
**50**	Insulin_resistance	2023	European	53287	https://pubmed.ncbi.nlm.nih.gov/37291194/
**51**	Sleep_duration	2018	European	91105	https://pubmed.ncbi.nlm.nih.gov/30531941/
**52**	kidney_function_GFR	2024	European	406504	https://pubmed.ncbi.nlm.nih.gov/39256582/

Supplementary Table 1: SNP-based heritability estimates of IC using GCTA-REML in the UKB and CLSA.

V (G) = variance explained by genetic variability, V(e) = variance explained by non-genetic (environmental) factors, V(P) = variance in population, V(G)/V(P) = proportion of total IC variability in the population explained by genetic variation. The rest are test statistics parameters; logL = loglikelihood, LRT = likelihood ratio test, df =degree of freedom, Pval = P value, n = sample size.

**SNP-based heritability statistics, UKB**			**SNP-based heritability statistics, CLSA**		
**Source**	**Variance**	**SE**	**Source**	**Variance**	**SE**
**V(G)**	0.062475	0.002669	V(G)	0.071038	0.010012
**V(e)**	0.185960	0.002598	V(e)	0.293082	0.010202
**Vp**	0.248435	0.001701	Vp	0.364120	0.004526
V(G)/Vp	***0.251474***	0.010249	***V(G)/Vp***	***0.195095***	0.027156
**logL**	9112.565		logL	24.187	
**logL0**	8726.085		logL0	-12.497	
**LRT**	772.960		LRT	73.368	
**df**	1		df	1	
**Pval**	0.0000e+00		Pval	0.0000e+00	
**n**	44631		n	13085	

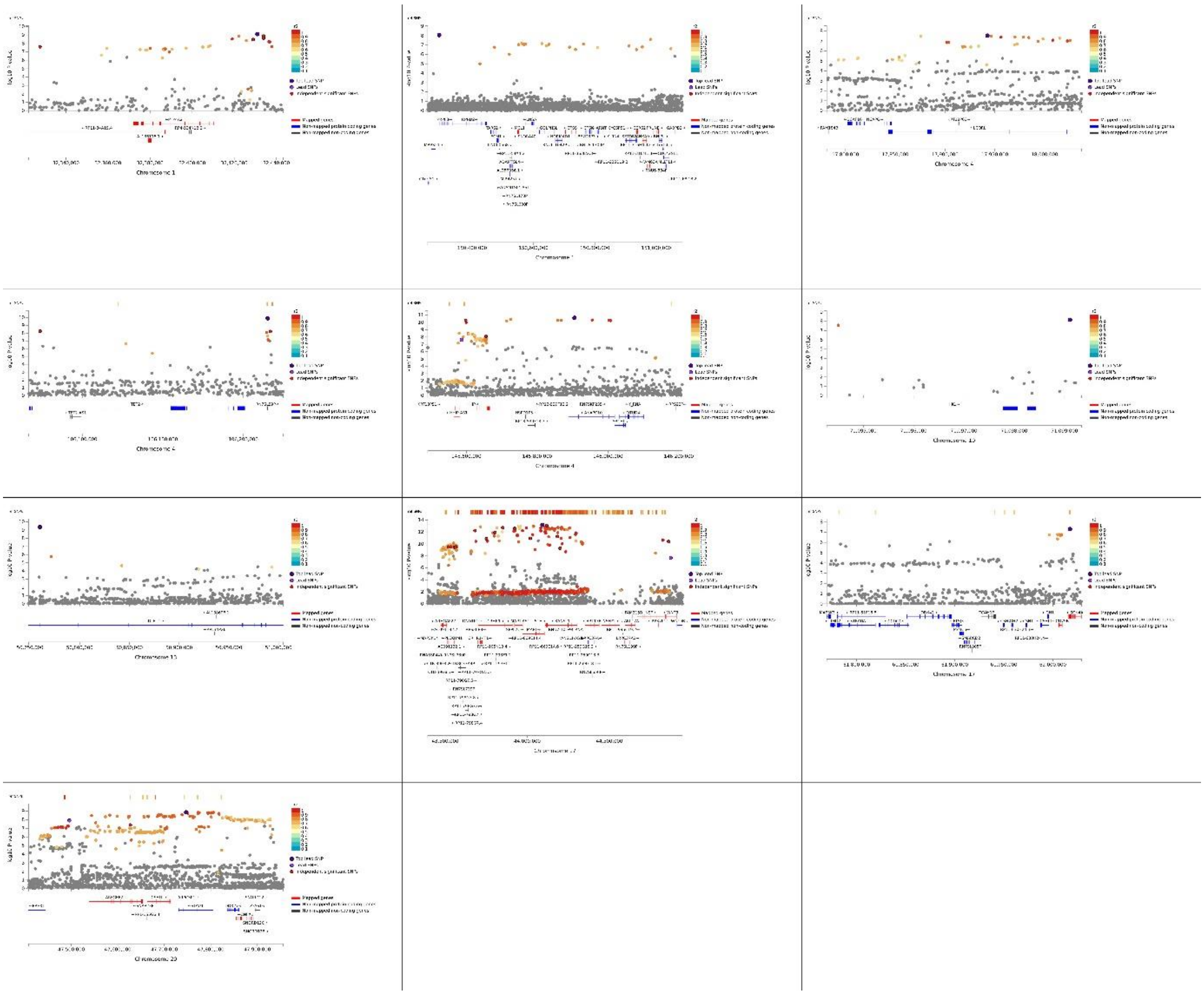

Supplementary Figure 1: Regional plots for ten loci associated with intrinsic capacity, including nearby genes and functional annotations.

In each plot, the circles represent individual SNPs, with the x-axis showing chromosomal position and the y-axis displaying the statistical significance of the associations (−log10(P-value)). Red circles denote independent significant SNPs, light purple circles represent lead SNPs, and bold purple circles highlight the top lead SNPs.

Supplementary Table 2: The ten genomic loci, Meta GWAS, UKB, and CLSA.

Locus	Lead SNPs	Chr	Start	End	A1	A2	MAF (%)	P-value	Nearby genes	P_UKB	P-CLSA	Het_Isq	Het_Pval (Cochran's Q)
**1**	rs72666714 (intergenic)	1	32327685	32437775	C	G	8.5	$7.9\text{x}10^{-10}$	*PTP4A2*	*5.09E-09*	*0.0469709*	*0.308*	*0.5791*
**2**	rs11581793 (intergenic)	1	150292561	151047648	T	C	0.3	$8.7\ \text{x}10^{-9}$	*PRPF3*	*6.69E-07*	*0.00256761*	*0*	*0.4094*
**3**	rs2707450 (intronic)	4	17792869	18025484	T	C	28.6	$2.9\ \text{x}10^{-8}$	*LCORL*	*8.42E-08*	*0.106674*	*0*	*0.4693*
**4**	rs200768290 (downstream)	4	106073735	106217588	T	C	44.4	$1.3\ \text{x}10^{-10}$	*RN7SL89P*	*1.25E-10*	-	*0*	*1*
**5**	rs200457388 (intergenic)	4	145523071	146177583	CA	C	28.9	$2.4\ \text{x}10^{-11}$	*ANAPC10*	*1.75E-11*	*0.00752433*	*57.7*	*0.124*
**6**	rs72805692 (intronic)	10	71094504	71099109	A	G	3.6	$6.9\ \text{x}10^{-9}$	*HK1*	*1.90E-10*	*0.906707*	*85.8*	*0.008032*
**7**	rs182234180 (intergenic)	13	50760125	50993083	T	C	0.8	$4.3\ \text{x}10^{-10}$	*DLEU1*	*4.32E-10*	-	*0*	*1*
**8**	rs9891103 (intronic)	17	43463493	44874453	T	C	17.7	$6.5\ \text{x}10^{-14}$	*MAPT*	*2.79E-12*	*0.00613923*	*0*	*0.7208*
**9**	rs2532111 (UTR3)	17	61781331	62017421	A	G	40.4	$4.9\ \text{x}10^{-8}$	*SCN4A*	*1.75E-07*	*0.0931071*	*0*	*0.5512*
**10**	rs34841991 (intronic)	20	47432799	47930866	T	C	15.5	$1.4\ \text{x}10^{-9}$	*STAU1*	*1.37E-08*	*0.0320122*	*0*	*0.7565*

Supplementary Table 3: IC Candidate SNPs reported in GWAS catalog

Genomic Locus	IndSigSNP	chr	bp	snp	Date Added To Catalog	PMID	Link	Trait
1	1:32327685	1	32356815	rs34701865	24/08/2021	34021172	www.ncbi.nlm.nih.gov/pubmed/34021172	Hip circumference adjusted for BMI
1	1:32327685	1	32397127	rs72665000	17/04/2020	31669095	www.ncbi.nlm.nih.gov/pubmed/31669095	Waist circumference adjusted for body mass index
2	1:150292561	1	150551327	rs11580946	29/06/2021	33462484	www.ncbi.nlm.nih.gov/pubmed/33462484	Alanine aminotransferase levels
2	1:150292561	1	150551327	rs11580946	29/06/2021	33462484	www.ncbi.nlm.nih.gov/pubmed/33462484	Aspartate aminotransferase to alanine aminotransferase ratio
2	1:150292561	1	150551327	rs11580946	29/06/2021	33462484	www.ncbi.nlm.nih.gov/pubmed/33462484	Gamma glutamyl transferase levels
2	1:150292561	1	150551327	rs11580946	21/09/2021	33230300	www.ncbi.nlm.nih.gov/pubmed/33230300	Pulse pressure
2	1:150292561	1	150551327	rs11580946	21/09/2021	33230300	www.ncbi.nlm.nih.gov/pubmed/33230300	Systolic blood pressure
2	1:150292561	1	150551327	rs11580946	8/02/2019	30595370	www.ncbi.nlm.nih.gov/pubmed/30595370	Systolic blood pressure
2	1:150292561	1	150292561	rs11581793	22/03/2021	33339817	www.ncbi.nlm.nih.gov/pubmed/33339817	Alanine aminotransferase levels
2	1:150292561	1	150425376	rs187799739	21/05/2019	30595370	www.ncbi.nlm.nih.gov/pubmed/30595370	Height
2	1:150292561	1	150425376	rs187799739	8/02/2019	30595370	www.ncbi.nlm.nih.gov/pubmed/30595370	Lung function (FVC)
2	1:150292561	1	150562043	rs190596489	16/11/2018	30072576	www.ncbi.nlm.nih.gov/pubmed/30072576	Blood protein levels
2	1:150292561	1	150954720	rs28521412	8/02/2019	30593698	www.ncbi.nlm.nih.gov/pubmed/30593698	Fat-free mass
2	1:150292561	1	150954720	rs28521412	8/02/2019	30593698	www.ncbi.nlm.nih.gov/pubmed/30593698	Fat-free mass
2	1:150292561	1	150954720	rs28521412	24/08/2021	34021172	www.ncbi.nlm.nih.gov/pubmed/34021172	Hip circumference adjusted for BMI
2	1:150292561	1	150981225	rs6684154	13/08/2021	34315874	www.ncbi.nlm.nih.gov/pubmed/34315874	Alanine aminotransferase levels
2	1:150292561	1	150981225	rs6684154	13/08/2021	34315874	www.ncbi.nlm.nih.gov/pubmed/34315874	Aspartate aminotransferase levels
2	1:150292561	1	150981225	rs6684154	9/06/2021	33972514	www.ncbi.nlm.nih.gov/pubmed/33972514	Liver enzyme levels (alanine transaminase)
2	1:150292561	1	150717488	rs72704611	17/04/2020	31669095	www.ncbi.nlm.nih.gov/pubmed/31669095	Waist-to-hip ratio adjusted for BMI

3	4:17942560	4	17792869	rs12640250	8/01/2020	30297969	www.ncbi.nlm.nih.gov/pubmed/30297969	Type 2 diabetes
3	4:17942560	4	18015156	rs16896276	26/01/2021	32193507	www.ncbi.nlm.nih.gov/pubmed/32193507	Corneal curvature
3	4:17942560	4	18015156	rs16896276	26/01/2021	32193507	www.ncbi.nlm.nih.gov/pubmed/32193507	Corneal curvature
3	4:17942560	4	18015156	rs16896276	26/01/2021	32193507	www.ncbi.nlm.nih.gov/pubmed/32193507	Corneal curvature
3	4:17942560	4	18015156	rs16896276	16/11/2022	34791242	www.ncbi.nlm.nih.gov/pubmed/34791242	Orbital telorism
3	4:17942560	4	18015156	rs16896276	17/04/2020	31669095	www.ncbi.nlm.nih.gov/pubmed/31669095	Waist circumference adjusted for body mass index
3	4:17942560	4	18025484	rs2011603	19/10/2018	30038396	www.ncbi.nlm.nih.gov/pubmed/30038396	Educational attainment (years of education)
3	4:17942560	4	18025484	rs2011603	7/03/2019	30578418	www.ncbi.nlm.nih.gov/pubmed/30578418	Pulse pressure
3	4:17942560	4	18025484	rs2011603	14/09/2020	32541925	www.ncbi.nlm.nih.gov/pubmed/32541925	Type 2 diabetes
3	4:17942560	4	17998426	rs2061456	15/01/2020	31097437	www.ncbi.nlm.nih.gov/pubmed/31097437	Birth weight
3	4:17942560	4	17998426	rs2061456	17/07/2019	28552196	www.ncbi.nlm.nih.gov/pubmed/28552196	Height
3	4:17942560	4	17998426	rs2061456	5/08/2022	35831902	www.ncbi.nlm.nih.gov/pubmed/35831902	Height
3	4:17942560	4	17812615	rs2074974	24/08/2021	34021172	www.ncbi.nlm.nih.gov/pubmed/34021172	Hip circumference adjusted for BMI
3	4:17942560	4	17917781	rs2174633	28/01/2019	30598549	www.ncbi.nlm.nih.gov/pubmed/30598549	Heel bone mineral density
3	4:17942560	4	17917781	rs2174633	8/02/2019	30595370	www.ncbi.nlm.nih.gov/pubmed/30595370	Heel bone mineral density
3	4:17942560	4	17917781	rs2174633	18/10/2018	30048462	www.ncbi.nlm.nih.gov/pubmed/30048462	Heel bone mineral density
3	4:17942560	4	17917781	rs2174633	5/08/2019	31043758	www.ncbi.nlm.nih.gov/pubmed/31043758	Offspring birth weight
3	4:17942560	4	17972372	rs2320299	21/09/2021	33230300	www.ncbi.nlm.nih.gov/pubmed/33230300	Diastolic blood pressure
3	4:17942560	4	17972372	rs2320299	21/09/2021	33230300	www.ncbi.nlm.nih.gov/pubmed/33230300	Hypertension
3	4:17942560	4	17972372	rs2320299	21/09/2021	33230300	www.ncbi.nlm.nih.gov/pubmed/33230300	Pulse pressure
3	4:17942560	4	17972372	rs2320299	21/09/2021	33230300	www.ncbi.nlm.nih.gov/pubmed/33230300	Systolic blood pressure
3	4:17942560	4	18022834	rs2610989	20/06/2022	35315439	www.ncbi.nlm.nih.gov/pubmed/35315439	BMI at 1 year old
3	4:17942560	4	18022834	rs2610989	20/06/2022	35315439	www.ncbi.nlm.nih.gov/pubmed/35315439	BMI at 1.5 years old
3	4:17942560	4	18022834	rs2610989	20/06/2022	35315439	www.ncbi.nlm.nih.gov/pubmed/35315439	BMI at 2 years old
3	4:17942560	4	18022834	rs2610989	20/06/2022	35315439	www.ncbi.nlm.nih.gov/pubmed/35315439	BMI at 5 years old
3	4:17942560	4	18022834	rs2610989	20/06/2022	35315439	www.ncbi.nlm.nih.gov/pubmed/35315439	BMI at 6 months old
3	4:17942560	4	18022834	rs2610989	20/06/2022	35315439	www.ncbi.nlm.nih.gov/pubmed/35315439	BMI at 8 months old
3	4:17942560	4	18008232	rs2610990	1/07/2022	35361970	www.ncbi.nlm.nih.gov/pubmed/35361970	Educational attainment

3	4:17942560	4	18008232	rs2610990	24/08/2021	34021172	www.ncbi.nlm.nih.gov/pubmed/34021172	Hip circumference adjusted for BMI
3	4:17942560	4	18008232	rs2610990	29/11/2018	30224653	www.ncbi.nlm.nih.gov/pubmed/30224653	Systolic blood pressure
3	4:17942560	4	17942560	rs2707450	16/10/2019	31562340	www.ncbi.nlm.nih.gov/pubmed/31562340	Height
3	4:17942560	4	17942560	rs2707450	7/03/2019	30578418	www.ncbi.nlm.nih.gov/pubmed/30578418	Systolic blood pressure
3	4:17942560	4	17946432	rs2724475	24/10/2012	20881960	www.ncbi.nlm.nih.gov/pubmed/20881960	Height
3	4:17942560	4	17946432	rs2724475	2/07/2019	31217584	www.ncbi.nlm.nih.gov/pubmed/31217584	Height
3	4:17942560	4	17946432	rs2724475	26/04/2022	35399580	www.ncbi.nlm.nih.gov/pubmed/35399580	Height
3	4:17942560	4	17946432	rs2724475	14/12/2020	32042192	www.ncbi.nlm.nih.gov/pubmed/32042192	Sex hormone-binding globulin levels
3	4:17942560	4	17946432	rs2724475	14/12/2020	32042192	www.ncbi.nlm.nih.gov/pubmed/32042192	Sex hormone-binding globulin levels adjusted for BMI
3	4:17942560	4	17969698	rs2724485	24/08/2022	35762941	www.ncbi.nlm.nih.gov/pubmed/35762941	Systolic blood pressure
3	4:17942560	4	17903654	rs4144829	5/08/2019	31043758	www.ncbi.nlm.nih.gov/pubmed/31043758	Birth weight
3	4:17942560	4	17903654	rs4144829	13/08/2021	34128465	www.ncbi.nlm.nih.gov/pubmed/34128465	Lung volume
3	4:17942560	4	17903654	rs4144829	24/08/2021	34021172	www.ncbi.nlm.nih.gov/pubmed/34021172	Waist circumference adjusted for body mass index
3	4:17942560	4	17854055	rs6842303	16/12/2019	31575865	www.ncbi.nlm.nih.gov/pubmed/31575865	BMI at 1 year old
3	4:17942560	4	17854055	rs6842303	16/12/2019	31575865	www.ncbi.nlm.nih.gov/pubmed/31575865	BMI at 1.5 years old
3	4:17942560	4	17993410	rs724577	27/01/2020	31681408	www.ncbi.nlm.nih.gov/pubmed/31681408	Birth length (MTAG)
3	4:17942560	4	17993410	rs724577	28/02/2013	23202124	www.ncbi.nlm.nih.gov/pubmed/23202124	Birth weight
3	4:17942560	4	17993410	rs724577	27/01/2020	31681408	www.ncbi.nlm.nih.gov/pubmed/31681408	Birth weight (MTAG)
3	4:17942560	4	17993410	rs724577	8/11/2011	21998595	www.ncbi.nlm.nih.gov/pubmed/21998595	Height
3	4:17942560	4	17993410	rs724577	2/07/2019	31217584	www.ncbi.nlm.nih.gov/pubmed/31217584	Height
3	4:17942560	4	17871012	rs73242122	1/07/2022	35361970	www.ncbi.nlm.nih.gov/pubmed/35361970	Educational attainment
3	4:17942560	4	17798334	rs7679066	24/08/2021	34021172	www.ncbi.nlm.nih.gov/pubmed/34021172	Hip circumference adjusted for BMI
3	4:17942560	4	17919811	rs925098	15/01/2018	27680694	www.ncbi.nlm.nih.gov/pubmed/27680694	Birth weight
3	4:17942560	4	17919811	rs925098	25/11/2011	22021425	www.ncbi.nlm.nih.gov/pubmed/22021425	Height
3	4:17942560	4	17919811	rs925098	2/07/2019	31217584	www.ncbi.nlm.nih.gov/pubmed/31217584	Height
3	4:17942560	4	17919811	rs925098	1/07/2021	33713608	www.ncbi.nlm.nih.gov/pubmed/33713608	Height
3	4:17942560	4	17919811	rs925098	1/07/2021	33713608	www.ncbi.nlm.nih.gov/pubmed/33713608	Height
3	4:17942560	4	17919811	rs925098	1/07/2021	33713608	www.ncbi.nlm.nih.gov/pubmed/33713608	Height

3	4:17942560	4	17919811	rs925098	1/07/2021	33713608	www.ncbi.nlm.nih.gov/pubmed/33713608	Height
3	4:17942560	4	17919811	rs925098	14/12/2020	32042192	www.ncbi.nlm.nih.gov/pubmed/32042192	Sex hormone-binding globulin levels
3	4:17942560	4	17919811	rs925098	13/06/2022	35192695	www.ncbi.nlm.nih.gov/pubmed/35192695	Sex hormone-binding globulin levels
3	4:17942560	4	17919811	rs925098	13/06/2022	35192695	www.ncbi.nlm.nih.gov/pubmed/35192695	Sex hormone-binding globulin levels
3	4:17942560	4	17919811	rs925098	14/12/2020	32042192	www.ncbi.nlm.nih.gov/pubmed/32042192	Sex hormone-binding globulin levels adjusted for BMI
3	4:17942560	4	17901679	rs979532	24/08/2021	34021172	www.ncbi.nlm.nih.gov/pubmed/34021172	Hip circumference adjusted for BMI
3	4:17942560	4	17901679	rs979532	8/02/2019	30595370	www.ncbi.nlm.nih.gov/pubmed/30595370	Systolic blood pressure
5	4:106215109	4	106215109	rs200768290	29/06/2021	32895543	www.ncbi.nlm.nih.gov/pubmed/32895543	Verbal-numerical reasoning
5	4:106216800	4	106127121	rs34104813	14/12/2021	34594039	www.ncbi.nlm.nih.gov/pubmed/34594039	Neutrophil count
5	4:106216800	4	106127121	rs34104813	24/08/2022	35762941	www.ncbi.nlm.nih.gov/pubmed/35762941	Pulse pressure
5	4:106215109	4	106214476	rs57692580	2/09/2019	31263887	www.ncbi.nlm.nih.gov/pubmed/31263887	Mental health study participation (completed survey)
5	4:106216800	4	106143492	rs62332762	18/02/2021	33287642	www.ncbi.nlm.nih.gov/pubmed/33287642	Caffeine consumption from coffee or tea
6	4:145586459	4	145557651	rs11100862	30/07/2021	33104735	www.ncbi.nlm.nih.gov/pubmed/33104735	interferon-related traits
6	4:145905213	4	146133826	rs11733300	24/08/2021	34021172	www.ncbi.nlm.nih.gov/pubmed/34021172	Hip circumference adjusted for BMI
6	4:145597759	4	145599534	rs11946517	28/01/2019	30598549	www.ncbi.nlm.nih.gov/pubmed/30598549	Heel bone mineral density
6	4:145597759	4	145599534	rs11946517	8/02/2019	30595370	www.ncbi.nlm.nih.gov/pubmed/30595370	Heel bone mineral density
6	4:145597759	4	145599534	rs11946517	24/08/2021	34021172	www.ncbi.nlm.nih.gov/pubmed/34021172	Hip circumference adjusted for BMI
6	4:145586459	4	145567471	rs12507427	1/08/2019	31053729	www.ncbi.nlm.nih.gov/pubmed/31053729	Femoral neck size
6	4:145586459	4	145567471	rs12507427	1/08/2019	31053729	www.ncbi.nlm.nih.gov/pubmed/31053729	Hip bone size
6	4:145586459	4	145566848	rs13125694	16/10/2019	31562340	www.ncbi.nlm.nih.gov/pubmed/31562340	Height
6	4:145586459	4	145566848	rs13125694	5/08/2022	35831902	www.ncbi.nlm.nih.gov/pubmed/35831902	Height
6	4:145586459	4	145566848	rs13125694	14/12/2021	34594039	www.ncbi.nlm.nih.gov/pubmed/34594039	Height
6	4:145586459	4	145566848	rs13125694	24/08/2021	34021172	www.ncbi.nlm.nih.gov/pubmed/34021172	Hip circumference adjusted for BMI
6	4:145586459	4	145566848	rs13125694	24/08/2021	34021172	www.ncbi.nlm.nih.gov/pubmed/34021172	Hip index
6	4:145586459	4	145569692	rs13146972	26/04/2022	35399580	www.ncbi.nlm.nih.gov/pubmed/35399580	Height
6	4:145655389	4	145638178	rs1389029	4/08/2022	35505052	www.ncbi.nlm.nih.gov/pubmed/35505052	Whole brain restricted isotropic diffusion (multivariate analysis)
6	4:145655389	4	145619413	rs1492819	24/08/2021	34021172	www.ncbi.nlm.nih.gov/pubmed/34021172	Waist circumference adjusted for body mass index

6	4:145655389	4	145650021	rs1492820	12/09/2008	18391950	www.ncbi.nlm.nih.gov/pubmed/18391950	Height
6	4:145655389	4	145650021	rs1492820	2/07/2019	31217584	www.ncbi.nlm.nih.gov/pubmed/31217584	Height
6	4:145586459	4	145544836	rs17776795	15/01/2020	31097437	www.ncbi.nlm.nih.gov/pubmed/31097437	Birth weight
6	4:145905213	4	145905213	rs200457388	7/02/2020	31761296	www.ncbi.nlm.nih.gov/pubmed/31761296	Appendicular lean mass
6	4:145905213	4	145905213	rs200457388	24/08/2021	34021172	www.ncbi.nlm.nih.gov/pubmed/34021172	Hip circumference adjusted for BMI
6	4:145905213	4	145905213	rs200457388	24/08/2021	34021172	www.ncbi.nlm.nih.gov/pubmed/34021172	Waist-hip index
6	4:145905213	4	145905213	rs200457388	24/08/2021	34021172	www.ncbi.nlm.nih.gov/pubmed/34021172	Waist-to-hip ratio adjusted for BMI
6	4:145586459	4	145599908	rs2131354	17/07/2019	28552196	www.ncbi.nlm.nih.gov/pubmed/28552196	Height
6	4:145586459	4	145599908	rs2131354	18/05/2021	32902719	www.ncbi.nlm.nih.gov/pubmed/32902719	Height
6	4:145586459	4	145599908	rs2131354	17/07/2019	28552196	www.ncbi.nlm.nih.gov/pubmed/28552196	Hip circumference adjusted for BMI
6	4:145586459	4	145599908	rs2131354	5/08/2019	31043758	www.ncbi.nlm.nih.gov/pubmed/31043758	Offspring birth weight
6	4:145586459	4	145599908	rs2131354	4/08/2022	35505052	www.ncbi.nlm.nih.gov/pubmed/35505052	Whole brain restricted directional diffusion (multivariate analysis)
6	4:145905213	4	145713769	rs4576021	7/02/2020	31761296	www.ncbi.nlm.nih.gov/pubmed/31761296	Appendicular lean mass
6	4:145905213	4	145713769	rs4576021	18/10/2018	30048462	www.ncbi.nlm.nih.gov/pubmed/30048462	Heel bone mineral density
6	4:145655389	4	145655389	rs55817790	24/08/2021	34021172	www.ncbi.nlm.nih.gov/pubmed/34021172	Waist-hip index
6	4:145655389	4	145655389	rs55817790	24/08/2021	34021172	www.ncbi.nlm.nih.gov/pubmed/34021172	Waist-to-hip ratio adjusted for BMI
6	4:145905213	4	145724712	rs6537315	28/05/2020	32231278	www.ncbi.nlm.nih.gov/pubmed/32231278	Refractive error
6	4:145905213	4	146007706	rs67365433	7/02/2020	31761296	www.ncbi.nlm.nih.gov/pubmed/31761296	Appendicular lean mass
6	4:145905213	4	146007706	rs67365433	24/08/2021	34021172	www.ncbi.nlm.nih.gov/pubmed/34021172	Waist-hip index
6	4:145905213	4	146007706	rs67365433	24/08/2021	34021172	www.ncbi.nlm.nih.gov/pubmed/34021172	Waist-to-hip ratio adjusted for BMI
6	4:145655389	4	145613807	rs6812830	14/02/2022	35079123	www.ncbi.nlm.nih.gov/pubmed/35079123	Total cerebellar volume (excluding Crus I vermis)
6	4:145655389	4	145626414	rs6817771	26/04/2022	35399580	www.ncbi.nlm.nih.gov/pubmed/35399580	Height
6	4:145655389	4	145653694	rs6827641	4/08/2022	35505052	www.ncbi.nlm.nih.gov/pubmed/35505052	Whole brain free water diffusion (multivariate analysis)
6	4:145586459	4	145565826	rs6845999	5/08/2019	31043758	www.ncbi.nlm.nih.gov/pubmed/31043758	Birth weight
6	4:145586459	4	145565826	rs6845999	15/07/2015	25429064	www.ncbi.nlm.nih.gov/pubmed/25429064	Height
6	4:145586459	4	145565826	rs6845999	2/07/2019	31217584	www.ncbi.nlm.nih.gov/pubmed/31217584	Height
6	4:145586459	4	145565826	rs6845999	26/04/2022	35399580	www.ncbi.nlm.nih.gov/pubmed/35399580	Height

6	4:145586459	4	145565826	rs6845999	5/08/2022	35831902	www.ncbi.nlm.nih.gov/pubmed/35831902	Height
6	4:145655389	4	145643079	rs6854783	17/09/2008	18391952	www.ncbi.nlm.nih.gov/pubmed/18391952	Height
6	4:145586459	4	145572046	rs7675744	24/08/2021	34021172	www.ncbi.nlm.nih.gov/pubmed/34021172	Hip circumference adjusted for BMI
6	4:145586459	4	145536336	rs7692554	25/03/2022	34270706	www.ncbi.nlm.nih.gov/pubmed/34270706	Height
6	4:145586459	4	145591294	rs77362667	25/03/2022	34270706	www.ncbi.nlm.nih.gov/pubmed/34270706	Height
6	4:145905213	4	145868370	rs789351	24/08/2021	34021172	www.ncbi.nlm.nih.gov/pubmed/34021172	Hip circumference adjusted for BMI
6	4:145905213	4	145868370	rs789351	4/11/2019	30239722	www.ncbi.nlm.nih.gov/pubmed/30239722	Waist-hip ratio
6	4:145586459	4	145586459	rs982903	17/04/2020	31669095	www.ncbi.nlm.nih.gov/pubmed/31669095	Waist circumference adjusted for body mass index
7	10:71099109	10	71094504	rs17476364	21/07/2020	32203549	www.ncbi.nlm.nih.gov/pubmed/32203549	Apolipoprotein B levels
7	10:71099109	10	71094504	rs17476364	10/06/2021	34059833	www.ncbi.nlm.nih.gov/pubmed/34059833	Glycated hemoglobin levels
7	10:71099109	10	71094504	rs17476364	30/08/2017	27863252	www.ncbi.nlm.nih.gov/pubmed/27863252	Hematocrit
7	10:71099109	10	71094504	rs17476364	22/03/2021	32171239	www.ncbi.nlm.nih.gov/pubmed/32171239	Hematocrit
7	10:71099109	10	71094504	rs17476364	22/03/2021	32171239	www.ncbi.nlm.nih.gov/pubmed/32171239	Hematocrit
7	10:71099109	10	71094504	rs17476364	22/03/2021	32171239	www.ncbi.nlm.nih.gov/pubmed/32171239	Hematocrit
7	10:71099109	10	71094504	rs17476364	18/09/2020	32888494	www.ncbi.nlm.nih.gov/pubmed/32888494	Hematocrit
7	10:71099109	10	71094504	rs17476364	14/12/2021	34594039	www.ncbi.nlm.nih.gov/pubmed/34594039	Hematocrit
7	10:71099109	10	71094504	rs17476364	18/09/2020	32888494	www.ncbi.nlm.nih.gov/pubmed/32888494	Hemoglobin
7	10:71099109	10	71094504	rs17476364	14/12/2021	34594039	www.ncbi.nlm.nih.gov/pubmed/34594039	Hemoglobin
7	10:71099109	10	71094504	rs17476364	14/12/2021	34594039	www.ncbi.nlm.nih.gov/pubmed/34594039	Hemoglobin A1c levels
7	10:71099109	10	71094504	rs17476364	30/08/2017	27863252	www.ncbi.nlm.nih.gov/pubmed/27863252	Hemoglobin concentration
7	10:71099109	10	71094504	rs17476364	22/03/2021	32171239	www.ncbi.nlm.nih.gov/pubmed/32171239	Hemoglobin concentration
7	10:71099109	10	71094504	rs17476364	22/03/2021	32171239	www.ncbi.nlm.nih.gov/pubmed/32171239	Hemoglobin concentration
7	10:71099109	10	71094504	rs17476364	22/03/2021	32171239	www.ncbi.nlm.nih.gov/pubmed/32171239	Hemoglobin concentration
7	10:71099109	10	71094504	rs17476364	30/08/2017	27863252	www.ncbi.nlm.nih.gov/pubmed/27863252	High light scatter reticulocyte count
7	10:71099109	10	71094504	rs17476364	18/09/2020	32888494	www.ncbi.nlm.nih.gov/pubmed/32888494	High light scatter reticulocyte count
7	10:71099109	10	71094504	rs17476364	30/08/2017	27863252	www.ncbi.nlm.nih.gov/pubmed/27863252	High light scatter reticulocyte percentage of red cells
7	10:71099109	10	71094504	rs17476364	18/09/2020	32888494	www.ncbi.nlm.nih.gov/pubmed/32888494	High light scatter reticulocyte percentage of red cells

7	10:71099109	10	71094504	rs17476364	18/09/2020	32888494	www.ncbi.nlm.nih.gov/pubmed/32888494	Immature fraction of reticulocytes
7	10:71099109	10	71094504	rs17476364	22/03/2021	33536631	www.ncbi.nlm.nih.gov/pubmed/33536631	Iron status biomarkers (ferritin levels)
7	10:71099109	10	71094504	rs17476364	21/07/2020	32203549	www.ncbi.nlm.nih.gov/pubmed/32203549	LDL cholesterol levels
7	10:71099109	10	71094504	rs17476364	17/11/2022	34887591	www.ncbi.nlm.nih.gov/pubmed/34887591	Low density lipoprotein cholesterol levels
7	10:71099109	10	71094504	rs17476364	17/11/2022	34887591	www.ncbi.nlm.nih.gov/pubmed/34887591	Low density lipoprotein cholesterol levels
7	10:71099109	10	71094504	rs17476364	30/08/2017	27863252	www.ncbi.nlm.nih.gov/pubmed/27863252	Mean corpuscular hemoglobin
7	10:71099109	10	71094504	rs17476364	18/09/2020	32888494	www.ncbi.nlm.nih.gov/pubmed/32888494	Mean corpuscular hemoglobin
7	10:71099109	10	71094504	rs17476364	21/09/2020	32888493	www.ncbi.nlm.nih.gov/pubmed/32888493	Mean corpuscular hemoglobin
7	10:71099109	10	71094504	rs17476364	21/09/2020	32888493	www.ncbi.nlm.nih.gov/pubmed/32888493	Mean corpuscular hemoglobin
7	10:71099109	10	71094504	rs17476364	8/02/2019	30595370	www.ncbi.nlm.nih.gov/pubmed/30595370	Mean corpuscular hemoglobin
7	10:71099109	10	71094504	rs17476364	14/12/2021	34594039	www.ncbi.nlm.nih.gov/pubmed/34594039	Mean corpuscular hemoglobin
7	10:71099109	10	71094504	rs17476364	30/08/2017	27863252	www.ncbi.nlm.nih.gov/pubmed/27863252	Mean corpuscular volume
7	10:71099109	10	71094504	rs17476364	22/03/2021	32171239	www.ncbi.nlm.nih.gov/pubmed/32171239	Mean corpuscular volume
7	10:71099109	10	71094504	rs17476364	18/09/2020	32888494	www.ncbi.nlm.nih.gov/pubmed/32888494	Mean corpuscular volume
7	10:71099109	10	71094504	rs17476364	21/09/2020	32888493	www.ncbi.nlm.nih.gov/pubmed/32888493	Mean corpuscular volume
7	10:71099109	10	71094504	rs17476364	21/09/2020	32888493	www.ncbi.nlm.nih.gov/pubmed/32888493	Mean corpuscular volume
7	10:71099109	10	71094504	rs17476364	12/11/2021	34104963	www.ncbi.nlm.nih.gov/pubmed/34104963	Mean corpuscular volume
7	10:71099109	10	71094504	rs17476364	14/12/2021	34594039	www.ncbi.nlm.nih.gov/pubmed/34594039	Mean corpuscular volume
7	10:71099109	10	71094504	rs17476364	18/09/2020	32888494	www.ncbi.nlm.nih.gov/pubmed/32888494	Mean reticulocyte volume
7	10:71099109	10	71094504	rs17476364	18/09/2020	32888494	www.ncbi.nlm.nih.gov/pubmed/32888494	Mean spheric corpuscular volume
7	10:71099109	10	71094504	rs17476364	17/11/2022	34887591	www.ncbi.nlm.nih.gov/pubmed/34887591	Non-HDL cholesterol levels
7	10:71099109	10	71094504	rs17476364	17/11/2022	34887591	www.ncbi.nlm.nih.gov/pubmed/34887591	Non-HDL cholesterol levels
7	10:71099109	10	71094504	rs17476364	13/12/2021	34446064	www.ncbi.nlm.nih.gov/pubmed/34446064	Obstructive sleep apnea trait (minimum oxyhemoglobin saturation across sleep episode)
7	10:71099109	10	71094504	rs17476364	21/09/2020	32888493	www.ncbi.nlm.nih.gov/pubmed/32888493	Platelet count
7	10:71099109	10	71094504	rs17476364	18/09/2020	32888494	www.ncbi.nlm.nih.gov/pubmed/32888494	Plateletcrit
7	10:71099109	10	71094504	rs17476364	30/08/2017	27863252	www.ncbi.nlm.nih.gov/pubmed/27863252	Red blood cell count
7	10:71099109	10	71094504	rs17476364	21/09/2020	32888493	www.ncbi.nlm.nih.gov/pubmed/32888493	Red blood cell count

7	10:71099109	10	71094504	rs17476364	21/09/2020	32888493	www.ncbi.nlm.nih.gov/pubmed/32888493	Red blood cell count
7	10:71099109	10	71094504	rs17476364	8/02/2019	30595370	www.ncbi.nlm.nih.gov/pubmed/30595370	Red blood cell count
7	10:71099109	10	71094504	rs17476364	18/09/2020	32888494	www.ncbi.nlm.nih.gov/pubmed/32888494	Red blood cell count
7	10:71099109	10	71094504	rs17476364	14/12/2021	34594039	www.ncbi.nlm.nih.gov/pubmed/34594039	Red blood cell count
7	10:71099109	10	71094504	rs17476364	30/08/2017	27863252	www.ncbi.nlm.nih.gov/pubmed/27863252	Red cell distribution width
7	10:71099109	10	71094504	rs17476364	21/09/2020	32888493	www.ncbi.nlm.nih.gov/pubmed/32888493	Red cell distribution width
7	10:71099109	10	71094504	rs17476364	21/09/2020	32888493	www.ncbi.nlm.nih.gov/pubmed/32888493	Red cell distribution width
7	10:71099109	10	71094504	rs17476364	18/09/2020	32888494	www.ncbi.nlm.nih.gov/pubmed/32888494	Red cell distribution width
7	10:71099109	10	71094504	rs17476364	8/02/2019	30595370	www.ncbi.nlm.nih.gov/pubmed/30595370	Red cell distribution width
7	10:71099109	10	71094504	rs17476364	30/08/2017	27863252	www.ncbi.nlm.nih.gov/pubmed/27863252	Reticulocyte count
7	10:71099109	10	71094504	rs17476364	18/09/2020	32888494	www.ncbi.nlm.nih.gov/pubmed/32888494	Reticulocyte count
7	10:71099109	10	71094504	rs17476364	30/08/2017	27863252	www.ncbi.nlm.nih.gov/pubmed/27863252	Reticulocyte fraction of red cells
7	10:71099109	10	71094504	rs17476364	18/09/2020	32888494	www.ncbi.nlm.nih.gov/pubmed/32888494	Reticulocyte fraction of red cells
7	10:71099109	10	71094504	rs17476364	14/12/2021	34594039	www.ncbi.nlm.nih.gov/pubmed/34594039	Total bilirubin levels
7	10:71099109	10	71094504	rs17476364	17/11/2022	34887591	www.ncbi.nlm.nih.gov/pubmed/34887591	Total cholesterol levels
7	10:71099109	10	71094504	rs17476364	17/11/2022	34887591	www.ncbi.nlm.nih.gov/pubmed/34887591	Total cholesterol levels
7	10:71099109	10	71099109	rs72805692	1/03/2022	34951656	www.ncbi.nlm.nih.gov/pubmed/34951656	Glycated haemoglobin HbA1c levels adjusted for BMI
7	10:71099109	10	71099109	rs72805692	1/03/2022	34951656	www.ncbi.nlm.nih.gov/pubmed/34951656	Glycated haemoglobin HbA1c levels adjusted for BMI
7	10:71099109	10	71099109	rs72805692	1/03/2022	34951656	www.ncbi.nlm.nih.gov/pubmed/34951656	Glycated haemoglobin HbA1c levels adjusted for BMI
7	10:71099109	10	71099109	rs72805692	12/08/2019	31213470	www.ncbi.nlm.nih.gov/pubmed/31213470	Glycated hemoglobin levels
7	10:71099109	10	71099109	rs72805692	10/06/2021	34059833	www.ncbi.nlm.nih.gov/pubmed/34059833	Glycated hemoglobin levels
7	10:71099109	10	71099109	rs72805692	21/07/2020	32203549	www.ncbi.nlm.nih.gov/pubmed/32203549	HDL cholesterol levels
7	10:71099109	10	71099109	rs72805692	28/01/2022	33887194	www.ncbi.nlm.nih.gov/pubmed/33887194	Hematocrit
7	10:71099109	10	71099109	rs72805692	28/01/2022	33887194	www.ncbi.nlm.nih.gov/pubmed/33887194	Hemoglobin
7	10:71099109	10	71099109	rs72805692	1/07/2019	31217584	www.ncbi.nlm.nih.gov/pubmed/31217584	Hemoglobin A1c levels
7	10:71099109	10	71099109	rs72805692	1/07/2019	31217584	www.ncbi.nlm.nih.gov/pubmed/31217584	Hemoglobin A1c levels
7	10:71099109	10	71099109	rs72805692	21/09/2020	32888493	www.ncbi.nlm.nih.gov/pubmed/32888493	Hemoglobin concentration

7	10:71099109	10	71099109	rs72805692	27/07/2021	34014839	www.ncbi.nlm.nih.gov/pubmed/34014839	hemolysis of donated blood (osmotic)
7	10:71099109	10	71099109	rs72805692	27/07/2021	34014839	www.ncbi.nlm.nih.gov/pubmed/34014839	hemolysis of donated blood (osmotic)
7	10:71099109	10	71099109	rs72805692	30/08/2017	27863252	www.ncbi.nlm.nih.gov/pubmed/27863252	Immature fraction of reticulocytes
7	10:71099109	10	71099109	rs72805692	28/01/2022	33887194	www.ncbi.nlm.nih.gov/pubmed/33887194	Mean corpuscular volume
7	10:71099109	10	71099109	rs72805692	22/02/2022	35047847	www.ncbi.nlm.nih.gov/pubmed/35047847	Nonalcoholic fatty liver disease (imputed)
7	10:71099109	10	71099109	rs72805692	15/03/2022	34743558	www.ncbi.nlm.nih.gov/pubmed/34743558	Retinal vascular fractal density
8	13:50760125	13	50993083	rs143280072	24/08/2021	34021172	www.ncbi.nlm.nih.gov/pubmed/34021172	Hip circumference adjusted for BMI
8	13:50760125	13	50993083	rs143280072	24/08/2021	34021172	www.ncbi.nlm.nih.gov/pubmed/34021172	Hip circumference adjusted for BMI
8	13:50760125	13	50760125	rs182234180	18/09/2020	32888494	www.ncbi.nlm.nih.gov/pubmed/32888494	Lymphocyte count
8	13:50760125	13	50760125	rs182234180	18/09/2020	32888494	www.ncbi.nlm.nih.gov/pubmed/32888494	Lymphocyte percentage of white cells
8	13:50760125	13	50771235	rs2703087	24/08/2021	34021172	www.ncbi.nlm.nih.gov/pubmed/34021172	Hip circumference adjusted for BMI
8	13:50760125	13	50771235	rs2703087	24/08/2021	34021172	www.ncbi.nlm.nih.gov/pubmed/34021172	Hip circumference adjusted for BMI
9	17:44118848	17	43932789	rs10445365	31/05/2022	33875891	www.ncbi.nlm.nih.gov/pubmed/33875891	IDP dMRI ProbtrackX L2 atr l
9	17:44118848	17	43932789	rs10445365	31/05/2022	33875891	www.ncbi.nlm.nih.gov/pubmed/33875891	IDP dMRI ProbtrackX MD atr l
9	17:44118848	17	43932789	rs10445365	31/05/2022	33875891	www.ncbi.nlm.nih.gov/pubmed/33875891	IDP dMRI TBSS ICVF Anterior corona radiata R
9	17:44118848	17	43932789	rs10445365	31/05/2022	33875891	www.ncbi.nlm.nih.gov/pubmed/33875891	IDP dMRI TBSS ICVF Anterior limb of internal capsule R
9	17:44118848	17	43932789	rs10445365	31/05/2022	33875891	www.ncbi.nlm.nih.gov/pubmed/33875891	IDP dMRI TBSS ICVF Retrolenticular part of internal capsule L
9	17:44118848	17	43932797	rs10445366	27/07/2021	34211149	www.ncbi.nlm.nih.gov/pubmed/34211149	Age at first birth
9	17:44118848	17	43932798	rs10445367	14/12/2021	34594039	www.ncbi.nlm.nih.gov/pubmed/34594039	Neutrophil count
9	17:44118848	17	43932798	rs10445367	5/02/2020	31666681	www.ncbi.nlm.nih.gov/pubmed/31666681	White matter microstructure (fractional anisotropy)
9	17:44118848	17	43933171	rs10445368	23/10/2020	32665545	www.ncbi.nlm.nih.gov/pubmed/32665545	Cortical surface area (MOSTest)
9	17:44118848	17	43950976	rs10491140	18/10/2018	30048462	www.ncbi.nlm.nih.gov/pubmed/30048462	Heel bone mineral density
9	17:44118848	17	44073889	rs1052553	26/04/2018	25751624	www.ncbi.nlm.nih.gov/pubmed/25751624	Type 1 diabetes
9	17:44118848	17	44073889	rs1052553	7/07/2021	34127860	www.ncbi.nlm.nih.gov/pubmed/34127860	Type 1 diabetes
9	17:44118848	17	44073889	rs1052553	7/07/2021	34127860	www.ncbi.nlm.nih.gov/pubmed/34127860	Type 1 diabetes
9	17:43564222	17	43513441	rs11012	5/02/2010	20070850	www.ncbi.nlm.nih.gov/pubmed/20070850	Parkinson's disease

9	17:44118848	17	43839951	rs11079718	18/09/2020	32888494	www.ncbi.nlm.nih.gov/pubmed/32888494	Reticulocyte count
9	17:44118848	17	43857989	rs111433752	13/12/2016	27067015	www.ncbi.nlm.nih.gov/pubmed/27067015	Neuroticism
9	17:44118848	17	44183403	rs111676341	13/01/2021	33414549	www.ncbi.nlm.nih.gov/pubmed/33414549	Cognitive aspects of educational attainment
9	17:44118848	17	44019107	rs111825734	31/05/2022	33875891	www.ncbi.nlm.nih.gov/pubmed/33875891	IDP dMRI TBSS MD Anterior corona radiata R
9	17:44118848	17	43666492	rs112010353	12/11/2018	30038396	www.ncbi.nlm.nih.gov/pubmed/30038396	Self-reported math ability
9	17:44118848	17	43785098	rs112123127	31/05/2022	33875891	www.ncbi.nlm.nih.gov/pubmed/33875891	IDP dMRI TBSS MD Anterior limb of internal capsule R
9	17:43564222	17	43563304	rs112275793	14/12/2021	34594039	www.ncbi.nlm.nih.gov/pubmed/34594039	Aspartate aminotransferase levels
9	17:44118848	17	43669491	rs112327620	14/12/2021	34594039	www.ncbi.nlm.nih.gov/pubmed/34594039	Hemoglobin
9	17:44118848	17	44126673	rs112333322	22/01/2019	29500382	www.ncbi.nlm.nih.gov/pubmed/29500382	Experiencing mood swings
9	17:44118848	17	44066172	rs112385572	3/12/2018	28196072	www.ncbi.nlm.nih.gov/pubmed/28196072	Male-pattern baldness
9	17:44118848	17	44019103	rs112568425	18/09/2020	32888494	www.ncbi.nlm.nih.gov/pubmed/32888494	Immature fraction of reticulocytes
9	17:44118848	17	44072984	rs112572874	24/10/2022	34321204	www.ncbi.nlm.nih.gov/pubmed/34321204	Free testosterone levels
9	17:44118848	17	44348634	rs112665297	5/02/2020	31666681	www.ncbi.nlm.nih.gov/pubmed/31666681	White matter microstructure (axial diusivities)
9	17:44118848	17	43695263	rs112997627	24/08/2021	33692100	www.ncbi.nlm.nih.gov/pubmed/33692100	Eye color
9	17:44118848	17	43569245	rs113322852	13/01/2021	33414549	www.ncbi.nlm.nih.gov/pubmed/33414549	Cognitive aspects of educational attainment
9	17:44118848	17	43569245	rs113322852	18/03/2019	30643256	www.ncbi.nlm.nih.gov/pubmed/30643256	Neuroticism
9	17:44118848	17	43850932	rs113518470	11/05/2017	27182965	www.ncbi.nlm.nih.gov/pubmed/27182965	Joint mobility (Beighton score)
9	17:44118848	17	43991272	rs113568679	15/03/2022	34743297	www.ncbi.nlm.nih.gov/pubmed/34743297	Alzheimer's disease or educational attainment (pleiotropy)
9	17:44091886	17	44228169	rs114974956	6/12/2019	31530798	www.ncbi.nlm.nih.gov/pubmed/31530798	Ventral diencephalon volume
9	17:44118848	17	44699851	rs116956554	8/10/2020	32769997	www.ncbi.nlm.nih.gov/pubmed/32769997	Thyroid stimulating hormone levels
9	17:44118848	17	44051588	rs117124984	25/06/2019	30804565	www.ncbi.nlm.nih.gov/pubmed/30804565	Daytime nap
9	17:44118848	17	44051589	rs118087478	28/01/2020	31676860	www.ncbi.nlm.nih.gov/pubmed/31676860	Brain region volumes
9	17:44118848	17	44015446	rs12150229	25/06/2019	30804565	www.ncbi.nlm.nih.gov/pubmed/30804565	Ease of getting up in the morning
9	17:44118848	17	43826637	rs12150672	11/05/2017	28017375	www.ncbi.nlm.nih.gov/pubmed/28017375	Red blood cell count
9	17:44118848	17	43923654	rs12185233	4/11/2019	30239722	www.ncbi.nlm.nih.gov/pubmed/30239722	Waist-to-hip ratio adjusted for BMI
9	17:44118848	17	43923683	rs12185268	8/07/2016	26077951	www.ncbi.nlm.nih.gov/pubmed/26077951	Corticobasal degeneration

9	17:44118848	17	43923683	rs12185268	20/07/2011	21738487	www.ncbi.nlm.nih.gov/pubmed/21738487	Parkinson's disease
9	17:44118848	17	43924073	rs12373123	24/01/2022	33637690	www.ncbi.nlm.nih.gov/pubmed/33637690	Alzheimer's disease (age of onset) in APOE e4 non-carriers
9	17:44118848	17	43924073	rs12373123	17/06/2020	32327693	www.ncbi.nlm.nih.gov/pubmed/32327693	Hemoglobin levels
9	17:44118848	17	43924219	rs12373124	15/08/2012	22693459	www.ncbi.nlm.nih.gov/pubmed/22693459	Male-pattern baldness
9	17:44118848	17	43924219	rs12373124	3/12/2018	28196072	www.ncbi.nlm.nih.gov/pubmed/28196072	Male-pattern baldness
9	17:44118848	17	43924200	rs12373142	1/05/2019	30804561	www.ncbi.nlm.nih.gov/pubmed/30804561	Chronic obstructive pulmonary disease
9	17:44118848	17	43924200	rs12373142	1/06/2021	33909500	www.ncbi.nlm.nih.gov/pubmed/33909500	Chronic obstructive pulmonary disease
9	17:43924337	17	43924337	rs12373168	5/09/2022	35764056	www.ncbi.nlm.nih.gov/pubmed/35764056	Attention deficit hyperactivity disorder or autism spectrum disorder or intelligence (pleiotropy)
9	17:43520118	17	43495420	rs12936645	3/08/2020	32352494	www.ncbi.nlm.nih.gov/pubmed/32352494	Spherical equivalent
9	17:43564222	17	43507297	rs12949256	17/06/2020	32327693	www.ncbi.nlm.nih.gov/pubmed/32327693	Hemoglobin levels
9	17:43803189	17	43803189	rs1358071	31/05/2022	33875891	www.ncbi.nlm.nih.gov/pubmed/33875891	IDP dMRI TBSS L1 Superior longitudinal fasciculus R
9	17:44118848	17	44787312	rs1378358	20/07/2020	32198502	www.ncbi.nlm.nih.gov/pubmed/32198502	Cortical surface area (global PC1)
9	17:44118848	17	44787312	rs1378358	22/01/2019	29500382	www.ncbi.nlm.nih.gov/pubmed/29500382	Feeling miserable
9	17:44118848	17	44787312	rs1378358	22/01/2019	29500382	www.ncbi.nlm.nih.gov/pubmed/29500382	Feeling worry
9	17:44118848	17	44787312	rs1378358	5/03/2021	33495596	www.ncbi.nlm.nih.gov/pubmed/33495596	Hypertrophic cardiomyopathy
9	17:44118848	17	44787312	rs1378358	5/03/2021	33495596	www.ncbi.nlm.nih.gov/pubmed/33495596	Hypertrophic cardiomyopathy (MTAG)
9	17:44118848	17	44787312	rs1378358	22/01/2019	29500382	www.ncbi.nlm.nih.gov/pubmed/29500382	Neurociticism
9	17:44118848	17	44787312	rs1378358	18/03/2019	30643256	www.ncbi.nlm.nih.gov/pubmed/30643256	Neuroticism
9	17:44118848	17	44335579	rs139077859	1/02/2022	34753499	www.ncbi.nlm.nih.gov/pubmed/34753499	Cardiorespiratory fitness (vo2 max)
9	17:44118848	17	44335579	rs139077859	31/05/2022	33875891	www.ncbi.nlm.nih.gov/pubmed/33875891	IDP dMRI TBSS L1 Superior longitudinal fasciculus L
9	17:44118848	17	43933790	rs142955985	14/12/2021	34594039	www.ncbi.nlm.nih.gov/pubmed/34594039	Eosinophil counts
9	17:44118848	17	43685698	rs143246821	1/11/2018	30061609	www.ncbi.nlm.nih.gov/pubmed/30061609	Lung function (FEV1)
9	17:44118848	17	43685698	rs143246821	1/11/2018	30061609	www.ncbi.nlm.nih.gov/pubmed/30061609	Lung function (FVC)

9	17:44118848	17	44132458	rs143364530	15/03/2022	34743297	www.ncbi.nlm.nih.gov/pubmed/34743297	Alzheimer's disease or educational attainment (pleiotropy)
9	17:44118848	17	43546057	rs143982995	15/03/2022	34743297	www.ncbi.nlm.nih.gov/pubmed/34743297	Alzheimer's disease or educational attainment (pleiotropy)
9	17:43564222	17	43564222	rs144733372	3/12/2018	28196072	www.ncbi.nlm.nih.gov/pubmed/28196072	Male-pattern baldness
9	17:43564222	17	43564222	rs144733372	10/07/2018	29559929	www.ncbi.nlm.nih.gov/pubmed/29559929	Remission after SSRI treatment in MDD or neuroticism
9	17:44118848	17	43569909	rs146746174	12/11/2018	30038396	www.ncbi.nlm.nih.gov/pubmed/30038396	Cognitive performance
9	17:44118848	17	43569909	rs146746174	12/11/2018	30038396	www.ncbi.nlm.nih.gov/pubmed/30038396	Cognitive performance (MTAG)
9	17:44118848	17	43569909	rs146746174	12/11/2018	30038396	www.ncbi.nlm.nih.gov/pubmed/30038396	Self-reported math ability (MTAG)
9	17:44118848	17	44335635	rs147431626	5/02/2020	31666681	www.ncbi.nlm.nih.gov/pubmed/31666681	White matter microstructure (radial diusivities)
9	17:44118848	17	43549608	rs149366495	4/05/2021	32963231	www.ncbi.nlm.nih.gov/pubmed/32963231	Cortical volume
9	17:44118848	17	43687072	rs150180355	15/03/2022	34743297	www.ncbi.nlm.nih.gov/pubmed/34743297	Alzheimer's disease or educational attainment (pleiotropy)
9	17:44874453	17	44874453	rs1563304	17/10/2018	30061737	www.ncbi.nlm.nih.gov/pubmed/30061737	Atrial fibrillation
9	17:44874453	17	44874453	rs1563304	22/01/2019	29500382	www.ncbi.nlm.nih.gov/pubmed/29500382	Experiencing mood swings
9	17:44874453	17	44874453	rs1563304	22/01/2019	29500382	www.ncbi.nlm.nih.gov/pubmed/29500382	Feeling hurt
9	17:44874453	17	44874453	rs1563304	31/10/2018	29942085	www.ncbi.nlm.nih.gov/pubmed/29942085	Neuroticism
9	17:44874453	17	44874453	rs1563304	1/02/2018	29255261	www.ncbi.nlm.nih.gov/pubmed/29255261	Neuroticism
9	17:44874453	17	44874453	rs1563304	25/06/2019	30804565	www.ncbi.nlm.nih.gov/pubmed/30804565	Snoring
9	17:44118848	17	43675489	rs1614886	31/05/2022	33875891	www.ncbi.nlm.nih.gov/pubmed/33875891	IDP dMRI TBSS MD Anterior limb of internal capsule L
9	17:43750921	17	43756969	rs1635288	31/05/2022	33875891	www.ncbi.nlm.nih.gov/pubmed/33875891	aparc-a2009s lh area S-collat-transv-ant
9	17:43750921	17	43751913	rs1635291	30/03/2020	31998841	www.ncbi.nlm.nih.gov/pubmed/31998841	Alcohol consumption (heavy vs. light/non-drinkers)
9	17:43750921	17	43744344	rs1635298	1/06/2021	33169155	www.ncbi.nlm.nih.gov/pubmed/33169155	Schizophrenia
9	17:44118848	17	44790203	rs169201	4/05/2021	32963231	www.ncbi.nlm.nih.gov/pubmed/32963231	Cortical volume
9	17:44118848	17	43908152	rs16940672	29/01/2020	31844048	www.ncbi.nlm.nih.gov/pubmed/31844048	Household income (MTAG)
9	17:44091886	17	44186063	rs16940904	31/08/2022	35692035	www.ncbi.nlm.nih.gov/pubmed/35692035	Circulating docosahexaenoic acid levels

9	17:44091886	17	44186063	rs16940904	27/04/2022	35213538	www.ncbi.nlm.nih.gov/pubmed/35213538	Docosahexaenoic acid levels
9	17:44091886	17	44186063	rs16940904	27/04/2022	35213538	www.ncbi.nlm.nih.gov/pubmed/35213538	Omega-3 fatty acid levels
9	17:44091886	17	44186063	rs16940904	27/04/2022	35213538	www.ncbi.nlm.nih.gov/pubmed/35213538	Ratio of docosahexaenoic acid to total fatty acid levels
9	17:44091886	17	44186063	rs16940904	27/04/2022	35213538	www.ncbi.nlm.nih.gov/pubmed/35213538	Ratio of omega-3 fatty acids to total fatty acids
9	17:44091886	17	44186063	rs16940904	31/08/2022	35692035	www.ncbi.nlm.nih.gov/pubmed/35692035	Total omega-3 fatty acid levels
9	17:44118848	17	43672118	rs1724417	4/08/2022	35585065	www.ncbi.nlm.nih.gov/pubmed/35585065	Baked/steamed fish liking
9	17:44118848	17	43672118	rs1724417	4/08/2022	35585065	www.ncbi.nlm.nih.gov/pubmed/35585065	F-fish liking (derived food-liking factor)
9	17:44118848	17	43672118	rs1724417	4/08/2022	35585065	www.ncbi.nlm.nih.gov/pubmed/35585065	F-oily fish liking (derived food-liking factor)
9	17:44118848	17	43672118	rs1724417	4/08/2022	35585065	www.ncbi.nlm.nih.gov/pubmed/35585065	F-seafood liking (derived food-liking factor)
9	17:44118848	17	43662020	rs1724429	14/09/2020	32451486	www.ncbi.nlm.nih.gov/pubmed/32451486	Problematic alcohol use (MTAG)
9	17:44118848	17	43656176	rs1724438	21/09/2020	32888493	www.ncbi.nlm.nih.gov/pubmed/32888493	Eosinophil counts
9	17:44118848	17	43656176	rs1724438	21/09/2020	32888493	www.ncbi.nlm.nih.gov/pubmed/32888493	Eosinophil counts
9	17:44118848	17	43656176	rs1724438	31/05/2022	33875891	www.ncbi.nlm.nih.gov/pubmed/33875891	IDP dMRI TBSS MD Cingulum cingulate gyrus R
9	17:44118848	17	43830938	rs17426174	17/11/2022	36324656	www.ncbi.nlm.nih.gov/pubmed/36324656	Disruptive behavior (multivariate analysis)
9	17:44118848	17	43830938	rs17426174	23/02/2018	29326435	www.ncbi.nlm.nih.gov/pubmed/29326435	Intelligence (MTAG)
9	17:44118848	17	43811072	rs17563683	30/08/2017	27863252	www.ncbi.nlm.nih.gov/pubmed/27863252	Hematocrit
9	17:44118848	17	43811072	rs17563683	30/08/2017	27863252	www.ncbi.nlm.nih.gov/pubmed/27863252	Hemoglobin concentration
9	17:44118848	17	43811072	rs17563683	17/06/2020	32327693	www.ncbi.nlm.nih.gov/pubmed/32327693	Hemoglobin levels
9	17:44118848	17	43991272	rs17563986	12/01/2018	29186694	www.ncbi.nlm.nih.gov/pubmed/29186694	Cognitive ability
9	17:44118848	17	43991272	rs17563986	12/01/2018	29186694	www.ncbi.nlm.nih.gov/pubmed/29186694	Cognitive ability (MTAG)
9	17:44118848	17	44006601	rs17564829	14/05/2018	22961000	www.ncbi.nlm.nih.gov/pubmed/22961000	Primary biliary cirrhosis
9	17:44118848	17	44187492	rs17577094	5/12/2014	24842889	www.ncbi.nlm.nih.gov/pubmed/24842889	Parkinson's disease
9	17:44118848	17	44192923	rs17577369	22/01/2019	29500382	www.ncbi.nlm.nih.gov/pubmed/29500382	Feeling miserable
9	17:44118848	17	43516402	rs17631303	2/08/2013	23544013	www.ncbi.nlm.nih.gov/pubmed/23544013	Ovarian cancer in BRCA1 mutation carriers
9	17:44118848	17	43516402	rs17631303	2/08/2013	23544013	www.ncbi.nlm.nih.gov/pubmed/23544013	Ovarian cancer in BRCA1 mutation carriers
9	17:44118848	17	43994648	rs17649553	9/05/2015	25064009	www.ncbi.nlm.nih.gov/pubmed/25064009	Parkinson's disease

9	17:44118848	17	43994648	rs17649553	24/10/2017	28892059	www.ncbi.nlm.nih.gov/pubmed/28892059	Parkinson's disease
9	17:44118848	17	44037491	rs17650842	22/01/2019	29500382	www.ncbi.nlm.nih.gov/pubmed/29500382	Irritable mood
9	17:44118848	17	44098967	rs17652520	29/01/2020	31844048	www.ncbi.nlm.nih.gov/pubmed/31844048	Household income
9	17:44118848	17	44098967	rs17652520	4/08/2022	35585065	www.ncbi.nlm.nih.gov/pubmed/35585065	Lager liking
9	17:44118848	17	44098967	rs17652520	21/06/2019	31015401	www.ncbi.nlm.nih.gov/pubmed/31015401	Medication use (anilides)
9	17:44118848	17	44098967	rs17652520	1/02/2018	29255261	www.ncbi.nlm.nih.gov/pubmed/29255261	Neuroticism
9	17:44118848	17	44098967	rs17652520	8/02/2019	30595370	www.ncbi.nlm.nih.gov/pubmed/30595370	Neuroticism
9	17:44118848	17	44192957	rs17661015	22/01/2019	29500382	www.ncbi.nlm.nih.gov/pubmed/29500382	Feeling hurt
9	17:44118848	17	44192957	rs17661015	22/01/2019	29500382	www.ncbi.nlm.nih.gov/pubmed/29500382	Irritable mood
9	17:44118848	17	44357351	rs17665188	22/01/2019	29500382	www.ncbi.nlm.nih.gov/pubmed/29500382	Experiencing mood swings
9	17:44118848	17	43906828	rs17689882	3/11/2015	25607358	www.ncbi.nlm.nih.gov/pubmed/25607358	Subcortical brain region volumes
9	17:44118848	17	43927290	rs17769552	13/09/2022	35935937	www.ncbi.nlm.nih.gov/pubmed/35935937	Eosinophil counts
9	17:44118848	17	43927290	rs17769552	9/07/2018	29691431	www.ncbi.nlm.nih.gov/pubmed/29691431	Hand grip strength
9	17:44118848	17	44219831	rs1819040	14/03/2022	34237774	www.ncbi.nlm.nih.gov/pubmed/34237774	COVID-19 (hospitalized vs population)
9	17:44118848	17	44219831	rs1819040	14/03/2022	34237774	www.ncbi.nlm.nih.gov/pubmed/34237774	SARS-CoV-2 infection
9	17:44118848	17	44359783	rs184837332	12/11/2021	34104963	www.ncbi.nlm.nih.gov/pubmed/34104963	Mean corpuscular volume
9	17:44118848	17	44408004	rs1863114	17/06/2020	32327693	www.ncbi.nlm.nih.gov/pubmed/32327693	Hemoglobin levels
9	17:44118848	17	43977827	rs1864325	11/04/2013	22504420	www.ncbi.nlm.nih.gov/pubmed/22504420	Lumbar spine bone mineral density
9	17:44118848	17	43682323	rs186806998	1/11/2018	30061609	www.ncbi.nlm.nih.gov/pubmed/30061609	Lung function (FEV1)
9	17:44118848	17	43682323	rs186806998	1/11/2018	30061609	www.ncbi.nlm.nih.gov/pubmed/30061609	Lung function (FEV1)
9	17:44118848	17	43682323	rs186806998	1/11/2018	30061609	www.ncbi.nlm.nih.gov/pubmed/30061609	Lung function (FVC)
9	17:44118848	17	43911443	rs1876829	22/09/2019	30575882	www.ncbi.nlm.nih.gov/pubmed/30575882	Waist-hip ratio
9	17:44118848	17	43911443	rs1876829	22/09/2019	30575882	www.ncbi.nlm.nih.gov/pubmed/30575882	Waist-to-hip ratio adjusted for BMI
9	17:44118848	17	43567337	rs1879586	4/08/2017	28346442	www.ncbi.nlm.nih.gov/pubmed/28346442	Epithelial ovarian cancer
9	17:44118848	17	43567337	rs1879586	7/08/2017	28346442	www.ncbi.nlm.nih.gov/pubmed/28346442	High-grade serous ovarian cancer
9	17:44118848	17	43567337	rs1879586	3/08/2017	28346442	www.ncbi.nlm.nih.gov/pubmed/28346442	Invasive epithelial ovarian cancer
9	17:44118848	17	43567337	rs1879586	3/08/2017	28346442	www.ncbi.nlm.nih.gov/pubmed/28346442	Ovarian cancer in BRCA1 mutation carriers

9	17:44118848	17	43567337	rs1879586	7/08/2017	28346442	www.ncbi.nlm.nih.gov/pubmed/28346442	Serous invasive ovarian cancer
9	17:44118848	17	44248769	rs1881193	22/01/2019	29500382	www.ncbi.nlm.nih.gov/pubmed/29500382	Experiencing mood swings
9	17:44091886	17	44228169	rs1918793	31/05/2022	33875891	www.ncbi.nlm.nih.gov/pubmed/33875891	IDP dMRI TBSS L3 Genu of corpus callosum
9	17:44118848	17	44218138	rs1918800	31/05/2022	33875891	www.ncbi.nlm.nih.gov/pubmed/33875891	ThalamNuclei lh volume VAmc
9	17:44118848	17	43991515	rs192818565	25/02/2017	27225129	www.ncbi.nlm.nih.gov/pubmed/27225129	Educational attainment (college completion)
9	17:44118848	17	43991515	rs192818565	24/02/2017	27225129	www.ncbi.nlm.nih.gov/pubmed/27225129	Educational attainment (years of education)
9	17:44118848	17	44142332	rs193236081	17/03/2017	27089181	www.ncbi.nlm.nih.gov/pubmed/27089181	Neuroticism
9	17:44118848	17	43539437	rs1971631	3/10/2022	34426670	www.ncbi.nlm.nih.gov/pubmed/34426670	Dietary macronutrient intake (multi-trait analysis)
9	17:44118848	17	44056767	rs1981997	1/11/2013	23583980	www.ncbi.nlm.nih.gov/pubmed/23583980	Interstitial lung disease
9	17:44118848	17	44083402	rs1991556	24/09/2019	31358974	www.ncbi.nlm.nih.gov/pubmed/31358974	Alcohol consumption
9	17:44118848	17	44083402	rs1991556	8/02/2019	30595370	www.ncbi.nlm.nih.gov/pubmed/30595370	Lung function (FVC)
9	17:44118848	17	44083402	rs1991556	18/01/2019	30531941	www.ncbi.nlm.nih.gov/pubmed/30531941	Sleep duration
9	17:44118848	17	44819565	rs199443	22/01/2019	29500382	www.ncbi.nlm.nih.gov/pubmed/29500382	Feeling fed-up
9	17:44118848	17	44819565	rs199443	27/09/2016	26632684	www.ncbi.nlm.nih.gov/pubmed/26632684	Sense of smell
9	17:44118848	17	44819565	rs199443	27/09/2016	26632684	www.ncbi.nlm.nih.gov/pubmed/26632684	Sense of smell
9	17:44118848	17	44812188	rs199447	31/10/2018	29942085	www.ncbi.nlm.nih.gov/pubmed/29942085	Neuroticism
9	17:44118848	17	44797919	rs199456	28/01/2019	30535121	www.ncbi.nlm.nih.gov/pubmed/30535121	Macular thickness
9	17:44874453	17	44866602	rs199497	19/08/2022	35872910	www.ncbi.nlm.nih.gov/pubmed/35872910	Atrial fibrillation (MTAG)
9	17:44874453	17	44866602	rs199497	12/11/2018	30038396	www.ncbi.nlm.nih.gov/pubmed/30038396	Cognitive performance (MTAG)
9	17:44874453	17	44866602	rs199497	2/05/2019	30867560	www.ncbi.nlm.nih.gov/pubmed/30867560	General factor of neuroticism
9	17:44874453	17	44866602	rs199497	27/09/2022	35396580	www.ncbi.nlm.nih.gov/pubmed/35396580	Schizophrenia
9	17:44118848	17	44865498	rs199499	30/08/2022	32193382	www.ncbi.nlm.nih.gov/pubmed/32193382	Alcohol consumption (drinks per month) (UKB data field 1578, 4424)
9	17:44118848	17	44865498	rs199499	3/02/2016	25778476	www.ncbi.nlm.nih.gov/pubmed/25778476	Alzheimer's disease in APOE e4- carriers
9	17:44118848	17	44862162	rs199503	15/03/2022	34743297	www.ncbi.nlm.nih.gov/pubmed/34743297	Alzheimer's disease or educational attainment (pleiotropy)
9	17:44118848	17	44862162	rs199503	27/04/2021	33686288	www.ncbi.nlm.nih.gov/pubmed/33686288	Schizophrenia vs autism spectrum disorder (ordinary least squares (OLS))

9	17:44118848	17	44862162	rs199503	24/08/2021	34021172	www.ncbi.nlm.nih.gov/pubmed/34021172	Waist-hip index
9	17:44118848	17	44862162	rs199503	24/08/2021	34021172	www.ncbi.nlm.nih.gov/pubmed/34021172	Waist-to-hip ratio adjusted for BMI
9	17:44861003	17	44861003	rs199504	4/05/2021	32963231	www.ncbi.nlm.nih.gov/pubmed/32963231	Cortical thickness
9	17:44861003	17	44859410	rs199505	31/10/2018	29942085	www.ncbi.nlm.nih.gov/pubmed/29942085	Depressed affect
9	17:44861003	17	44857929	rs199510	2/11/2021	34446935	www.ncbi.nlm.nih.gov/pubmed/34446935	Externalizing behaviour (multivariate analysis)
9	17:44861003	17	44857352	rs199512	8/10/2019	31504236	www.ncbi.nlm.nih.gov/pubmed/31504236	Handedness (Left-handed vs. non-left-handed)
9	17:44861003	17	44857352	rs199512	8/10/2019	31504236	www.ncbi.nlm.nih.gov/pubmed/31504236	Handedness (left-handed vs. right-handed)
9	17:44861003	17	44857352	rs199512	8/10/2019	31504236	www.ncbi.nlm.nih.gov/pubmed/31504236	Handedness (non-right-handed vs right-handed)
9	17:44861003	17	44856641	rs199515	6/04/2022	35379992	www.ncbi.nlm.nih.gov/pubmed/35379992	Alzheimer's disease
9	17:44861003	17	44856641	rs199515	20/04/2012	22451204	www.ncbi.nlm.nih.gov/pubmed/22451204	Parkinson's disease
9	17:44118848	17	44847834	rs199525	22/01/2019	29500382	www.ncbi.nlm.nih.gov/pubmed/29500382	Feeling guilty
9	17:44118848	17	44847834	rs199525	24/08/2021	34021172	www.ncbi.nlm.nih.gov/pubmed/34021172	Hip circumference adjusted for BMI
9	17:44118848	17	44847834	rs199525	17/04/2019	30818988	www.ncbi.nlm.nih.gov/pubmed/30818988	Intracranial volume
9	17:44118848	17	44847834	rs199525	1/11/2018	30061609	www.ncbi.nlm.nih.gov/pubmed/30061609	Lung function (FEV1)
9	17:44118848	17	44847834	rs199525	1/11/2018	30061609	www.ncbi.nlm.nih.gov/pubmed/30061609	Lung function (FEV1)
9	17:44118848	17	44847834	rs199525	1/11/2018	30061609	www.ncbi.nlm.nih.gov/pubmed/30061609	Lung function (FVC)
9	17:44118848	17	44847834	rs199525	1/11/2018	30061609	www.ncbi.nlm.nih.gov/pubmed/30061609	Lung function (FVC)
9	17:44118848	17	44843136	rs199528	4/10/2019	30679032	www.ncbi.nlm.nih.gov/pubmed/30679032	Alcohol consumption (drinks per week)
9	17:44118848	17	44828931	rs199533	24/01/2022	33637690	www.ncbi.nlm.nih.gov/pubmed/33637690	Alzheimer's disease (age of onset) (adjusted for APOE e4 carrier status)
9	17:44118848	17	44828931	rs199533	24/01/2022	33637690	www.ncbi.nlm.nih.gov/pubmed/33637690	Alzheimer's disease (age of onset) in APOE e4 non-carriers
9	17:44118848	17	44828931	rs199533	13/11/2020	32887889	www.ncbi.nlm.nih.gov/pubmed/32887889	Breast cancer
9	17:44118848	17	44828931	rs199533	13/11/2020	32887889	www.ncbi.nlm.nih.gov/pubmed/32887889	Cancer (pleiotropy)
9	17:44118848	17	44828931	rs199533	10/12/2009	19915575	www.ncbi.nlm.nih.gov/pubmed/19915575	Parkinson's disease
9	17:44118848	17	44828931	rs199533	22/09/2010	20711177	www.ncbi.nlm.nih.gov/pubmed/20711177	Parkinson's disease
9	17:44118848	17	44828931	rs199533	1/02/2022	34753499	www.ncbi.nlm.nih.gov/pubmed/34753499	Physical activity

9	17:44118848	17	44822662	rs199535	4/05/2021	32963231	www.ncbi.nlm.nih.gov/pubmed/32963231	Cortical surface area
9	17:44118848	17	44822662	rs199535	3/10/2022	34426670	www.ncbi.nlm.nih.gov/pubmed/34426670	Dietary macronutrient intake (multi-trait analysis)
9	17:44118848	17	44625866	rs199913382	17/06/2020	32327693	www.ncbi.nlm.nih.gov/pubmed/32327693	Hemoglobin levels
9	17:44118848	17	44214888	rs2077551	9/03/2020	31710517	www.ncbi.nlm.nih.gov/pubmed/31710517	Idiopathic pulmonary fibrosis
9	17:44118848	17	44214888	rs2077551	26/08/2022	35688625	www.ncbi.nlm.nih.gov/pubmed/35688625	Idiopathic pulmonary fibrosis
9	17:44118848	17	43919105	rs2106785	22/01/2019	29500382	www.ncbi.nlm.nih.gov/pubmed/29500382	Irritable mood
9	17:44118848	17	43919096	rs2106786	28/01/2020	31676860	www.ncbi.nlm.nih.gov/pubmed/31676860	Brain region volumes
9	17:44118848	17	43919096	rs2106786	30/08/2017	27863252	www.ncbi.nlm.nih.gov/pubmed/27863252	Red blood cell count
9	17:44118848	17	43953719	rs2214258	1/02/2018	29255261	www.ncbi.nlm.nih.gov/pubmed/29255261	Neuroticism
9	17:44118848	17	44222019	rs2222746	10/12/2020	32210231	www.ncbi.nlm.nih.gov/pubmed/32210231	Male puberty timing (age at voice breaking MTAG)
9	17:44118848	17	44222019	rs2222746	10/12/2020	32210231	www.ncbi.nlm.nih.gov/pubmed/32210231	Male puberty timing (early vs. average onset facial hair)
9	17:44118848	17	44222019	rs2222746	10/12/2020	32210231	www.ncbi.nlm.nih.gov/pubmed/32210231	Male puberty timing (late vs. average onset facial hair)
9	17:44118848	17	44222019	rs2222746	10/12/2020	32210231	www.ncbi.nlm.nih.gov/pubmed/32210231	Male puberty timing (late vs. average onset voice breaking)
9	17:44118848	17	44354549	rs2261201	22/01/2019	29500382	www.ncbi.nlm.nih.gov/pubmed/29500382	Irritable mood
9	17:44118848	17	44304884	rs2261497	31/05/2022	33875891	www.ncbi.nlm.nih.gov/pubmed/33875891	aparc-a2009s lh area S-orbital-H-Shaped
9	17:44118848	17	43919068	rs2316766	21/07/2020	32193296	www.ncbi.nlm.nih.gov/pubmed/32193296	Cortical thickness
9	17:44118848	17	43733983	rs241033	24/08/2022	35762941	www.ncbi.nlm.nih.gov/pubmed/35762941	Systolic blood pressure
9	17:44118848	17	43731719	rs241036	22/01/2019	29500382	www.ncbi.nlm.nih.gov/pubmed/29500382	Experiencing mood swings
9	17:44118848	17	43731719	rs241036	8/02/2019	30595370	www.ncbi.nlm.nih.gov/pubmed/30595370	Menarche (age at onset)
9	17:44118848	17	44327370	rs2458185	4/08/2022	35585065	www.ncbi.nlm.nih.gov/pubmed/35585065	Salami liking
9	17:44118848	17	43665468	rs2458198	31/05/2022	33875891	www.ncbi.nlm.nih.gov/pubmed/33875891	aparc-Desikan lh area TotalSurface
9	17:44118848	17	43665468	rs2458198	31/05/2022	33875891	www.ncbi.nlm.nih.gov/pubmed/33875891	aparc-Desikan rh area TotalSurface
9	17:44118848	17	43665468	rs2458198	31/05/2022	33875891	www.ncbi.nlm.nih.gov/pubmed/33875891	aparc-pial rh area TotalSurface
9	17:44118848	17	43665468	rs2458198	31/05/2022	33875891	www.ncbi.nlm.nih.gov/pubmed/33875891	IDP T1 SIENAX white normalised volume

9	17:44118848	17	43665468	rs2458198	31/05/2022	33875891	www.ncbi.nlm.nih.gov/pubmed/33875891	IDP T1 SIENAX white unnormalised volume
9	17:44118848	17	43671737	rs2463523	13/11/2020	32887889	www.ncbi.nlm.nih.gov/pubmed/32887889	Breast cancer
9	17:44118848	17	43671737	rs2463523	27/04/2022	35213538	www.ncbi.nlm.nih.gov/pubmed/35213538	Ratio of omega-6 fatty acids to omega-3 fatty acids
9	17:44118848	17	44285531	rs2469933	4/10/2019	30679032	www.ncbi.nlm.nih.gov/pubmed/30679032	Alcohol consumption (drinks per week)
9	17:44091886	17	44257783	rs2532252	31/05/2022	33875891	www.ncbi.nlm.nih.gov/pubmed/33875891	aparc-Desikan rh area TotalSurface
9	17:44118848	17	44256296	rs2532253	18/09/2020	32888494	www.ncbi.nlm.nih.gov/pubmed/32888494	Red cell distribution width
9	17:44118848	17	44252468	rs2532263	30/11/2017	29059683	www.ncbi.nlm.nih.gov/pubmed/29059683	Breast cancer
9	17:44118848	17	44252468	rs2532263	2/05/2019	30867560	www.ncbi.nlm.nih.gov/pubmed/30867560	General factor of neuroticism
9	17:44118848	17	44229986	rs2532300	7/06/2022	35255492	www.ncbi.nlm.nih.gov/pubmed/35255492	COVID-19 (critical illness vs population or mild symptoms)
9	17:44118848	17	44229986	rs2532300	7/06/2022	35255492	www.ncbi.nlm.nih.gov/pubmed/35255492	COVID-19 (critical illness vs population or mild symptoms)
9	17:44118848	17	44339473	rs2532349	13/09/2019	26423011	www.ncbi.nlm.nih.gov/pubmed/26423011	Lung function in never smokers (low FEV1 vs high FEV1)
9	17:44118848	17	44317210	rs2532384	4/08/2022	35585065	www.ncbi.nlm.nih.gov/pubmed/35585065	Goat cheese liking
9	17:44118848	17	44313757	rs2532386	26/11/2020	32589924	www.ncbi.nlm.nih.gov/pubmed/32589924	Osteoarthrosis (time to event)
9	17:44118848	17	44313522	rs2532387	4/08/2022	35585065	www.ncbi.nlm.nih.gov/pubmed/35585065	F-sharp flavour liking (derived food-liking factor)
9	17:44118848	17	44313522	rs2532387	13/12/2021	34469753	www.ncbi.nlm.nih.gov/pubmed/34469753	Lymphocyte-to-monocyte ratio
9	17:44118848	17	44311107	rs2532389	13/11/2020	32887889	www.ncbi.nlm.nih.gov/pubmed/32887889	Cancer (pleiotropy)
9	17:44118848	17	44307193	rs2532395	4/08/2022	35585065	www.ncbi.nlm.nih.gov/pubmed/35585065	Brussel sprout liking
9	17:44118848	17	44304130	rs2532402	4/08/2022	35585065	www.ncbi.nlm.nih.gov/pubmed/35585065	Horseradish liking
9	17:44118848	17	44304130	rs2532402	3/02/2022	35043453	www.ncbi.nlm.nih.gov/pubmed/35043453	Moderate to vigorous physical activity duration
9	17:44118848	17	44304130	rs2532402	3/02/2022	35043453	www.ncbi.nlm.nih.gov/pubmed/35043453	Physical activity (Total log acceleration)
9	17:44091886	17	44324539	rs2668639	31/05/2022	33875891	www.ncbi.nlm.nih.gov/pubmed/33875891	IDP dMRI ProbtrackX L1 slf r
9	17:44091886	17	44324539	rs2668639	31/05/2022	33875891	www.ncbi.nlm.nih.gov/pubmed/33875891	IDP dMRI TBSS L1 Superior longitudinal fasciculus R
9	17:44118848	17	43657440	rs2668674	5/03/2021	33495596	www.ncbi.nlm.nih.gov/pubmed/33495596	Left ventricle wall thickness
9	17:44118848	17	43657440	rs2668674	5/03/2021	33495596	www.ncbi.nlm.nih.gov/pubmed/33495596	Left ventricular mass

9	17:44118848	17	43657440	rs2668674	5/03/2021	33495596	www.ncbi.nlm.nih.gov/pubmed/33495596	Left ventricular mass to end-diastolic volume ratio
9	17:44118848	17	43659975	rs2668680	28/03/2019	30643251	www.ncbi.nlm.nih.gov/pubmed/30643251	Alcohol consumption (drinks per week) (MTAG)
9	17:44118848	17	44287373	rs2668690	27/04/2022	35213538	www.ncbi.nlm.nih.gov/pubmed/35213538	Phospholipids to total lipids ratio in small HDL
9	17:44118848	17	44287373	rs2668690	27/04/2022	35213538	www.ncbi.nlm.nih.gov/pubmed/35213538	Phospholipids to total lipids ratio in very large HDL
9	17:43520118	17	43520118	rs2684641	26/10/2022	36150907	www.ncbi.nlm.nih.gov/pubmed/36150907	Common executive function
9	17:44118848	17	44333889	rs2696422	31/05/2022	33875891	www.ncbi.nlm.nih.gov/pubmed/33875891	IDP SWI T2star left putamen
9	17:44118848	17	44283571	rs2696455	27/04/2022	35213538	www.ncbi.nlm.nih.gov/pubmed/35213538	Average diameter for HDL particles
9	17:44118848	17	44283571	rs2696455	31/05/2022	33875891	www.ncbi.nlm.nih.gov/pubmed/33875891	IDP dMRI TBSS MD Anterior limb of internal capsule L
9	17:44118848	17	44284057	rs2696457	22/01/2019	29500382	www.ncbi.nlm.nih.gov/pubmed/29500382	Feeling worry
9	17:44118848	17	44364649	rs2696497	22/01/2019	29500382	www.ncbi.nlm.nih.gov/pubmed/29500382	Feeling fed-up
9	17:44118848	17	44363929	rs2696500	22/01/2019	29500382	www.ncbi.nlm.nih.gov/pubmed/29500382	Neurociticism
9	17:44118848	17	44357885	rs2696518	31/05/2022	33875891	www.ncbi.nlm.nih.gov/pubmed/33875891	IDP dMRI ProbtrackX L1 ptr l
9	17:44118848	17	44357885	rs2696518	31/05/2022	33875891	www.ncbi.nlm.nih.gov/pubmed/33875891	IDP dMRI ProbtrackX L1 str r
9	17:44118848	17	44356793	rs2696524	5/02/2020	31666681	www.ncbi.nlm.nih.gov/pubmed/31666681	White matter microstructure (fractional anisotropy)
9	17:44118848	17	44355602	rs2696532	22/01/2019	29500382	www.ncbi.nlm.nih.gov/pubmed/29500382	Feeling guilty
9	17:44118848	17	44347218	rs2696557	22/01/2019	29500382	www.ncbi.nlm.nih.gov/pubmed/29500382	Feeling nervous
9	17:44118848	17	44347218	rs2696557	31/05/2022	33875891	www.ncbi.nlm.nih.gov/pubmed/33875891	IDP dMRI TBSS ICVF Superior fronto-occipital fasciculus R
9	17:44118848	17	44221836	rs2696589	1/02/2018	29255261	www.ncbi.nlm.nih.gov/pubmed/29255261	Neuroticism
9	17:44118848	17	44318663	rs2696591	4/08/2022	35505052	www.ncbi.nlm.nih.gov/pubmed/35505052	Whole brain restricted directional diffusion (multivariate analysis)
9	17:44091886	17	44318863	rs2696606	31/05/2022	33875891	www.ncbi.nlm.nih.gov/pubmed/33875891	aseg global volume BrainSegNotVentSurf
9	17:44091886	17	44318863	rs2696606	31/05/2022	33875891	www.ncbi.nlm.nih.gov/pubmed/33875891	aseg global volume SupraTentorialNotVent
9	17:44091886	17	44318863	rs2696606	31/05/2022	33875891	www.ncbi.nlm.nih.gov/pubmed/33875891	BA-exvivo rh area V2
9	17:44091886	17	44325635	rs2696618	31/05/2022	33875891	www.ncbi.nlm.nih.gov/pubmed/33875891	aparc-DKTatlas lh area fusiform

9	17:44118848	17	44326864	rs2696625	1/02/2022	34753499	www.ncbi.nlm.nih.gov/pubmed/34753499	Physical activity
9	17:44118848	17	44326864	rs2696625	18/01/2019	30531941	www.ncbi.nlm.nih.gov/pubmed/30531941	Physical activity (overall physical activity time)
9	17:44118848	17	44327482	rs2696630	31/05/2022	33875891	www.ncbi.nlm.nih.gov/pubmed/33875891	aparc-Desikan lh area fusiform
9	17:44118848	17	44245766	rs2696658	23/10/2020	32665545	www.ncbi.nlm.nih.gov/pubmed/32665545	Brain morphology (MOSTest)
9	17:44118848	17	44318184	rs2696668	22/01/2019	29500382	www.ncbi.nlm.nih.gov/pubmed/29500382	Feeling hurt
9	17:44118848	17	44310101	rs2696682	23/11/2022	35164939	www.ncbi.nlm.nih.gov/pubmed/35164939	Brain morphology (MOSTest)
9	17:44118848	17	44257788	rs2696697	18/08/2022	35589863	www.ncbi.nlm.nih.gov/pubmed/35589863	AlzheimerÔÇÖs disease polygenic risk score (upper quantile vs lower quantile)
9	17:44118848	17	44269546	rs2732596	29/01/2020	31844048	www.ncbi.nlm.nih.gov/pubmed/31844048	Household income
9	17:44118848	17	44364209	rs2732614	27/09/2016	26632684	www.ncbi.nlm.nih.gov/pubmed/26632684	Sense of smell
9	17:44118848	17	44364209	rs2732614	27/09/2016	26632684	www.ncbi.nlm.nih.gov/pubmed/26632684	Sense of smell
9	17:44118848	17	44289232	rs2732631	28/01/2019	30535121	www.ncbi.nlm.nih.gov/pubmed/30535121	Macular thickness
9	17:44118848	17	44344988	rs2732650	31/05/2022	33875891	www.ncbi.nlm.nih.gov/pubmed/33875891	IDP dMRI TBSS MD Anterior limb of internal capsule R
9	17:44118848	17	44344988	rs2732650	31/05/2022	33875891	www.ncbi.nlm.nih.gov/pubmed/33875891	IDP dMRI TBSS MD Cingulum cingulate gyrus L
9	17:44118848	17	44344988	rs2732650	21/01/2020	31518406	www.ncbi.nlm.nih.gov/pubmed/31518406	Loneliness
9	17:44118848	17	44346407	rs2732655	5/02/2020	31666681	www.ncbi.nlm.nih.gov/pubmed/31666681	White matter microstructure (fractional anisotropy)
9	17:44118848	17	44338503	rs2732689	4/08/2022	35585065	www.ncbi.nlm.nih.gov/pubmed/35585065	F-acquired taste liking (derived food-liking factor)
9	17:44118848	17	44354904	rs2732699	30/11/2017	29059683	www.ncbi.nlm.nih.gov/pubmed/29059683	Breast cancer
9	17:44118848	17	44353728	rs2732702	31/05/2022	33875891	www.ncbi.nlm.nih.gov/pubmed/33875891	aparc-Desikan lh area TotalSurface
9	17:44118848	17	44351686	rs2732706	14/01/2022	34957435	www.ncbi.nlm.nih.gov/pubmed/34957435	Stool frequency
9	17:44118848	17	44351387	rs2732708	22/01/2019	29500382	www.ncbi.nlm.nih.gov/pubmed/29500382	Feeling miserable
9	17:44118848	17	44351387	rs2732708	1/02/2018	29255261	www.ncbi.nlm.nih.gov/pubmed/29255261	Neuroticism
9	17:44118848	17	44321478	rs2732714	31/05/2022	33875891	www.ncbi.nlm.nih.gov/pubmed/33875891	IDP T1 SIENAX brain-normalised volume
9	17:44118848	17	44321478	rs2732714	31/05/2022	33875891	www.ncbi.nlm.nih.gov/pubmed/33875891	IDP T1 SIENAX brain-unnormalised volume
9	17:44118848	17	44321478	rs2732714	31/05/2022	33875891	www.ncbi.nlm.nih.gov/pubmed/33875891	IDP T1 SIENAX white normalised volume
9	17:44118848	17	44321478	rs2732714	31/05/2022	33875891	www.ncbi.nlm.nih.gov/pubmed/33875891	IDP T1 SIENAX white unnormalised volume

9	17:44296846	17	43688317	rs28768976	27/07/2021	33495597	www.ncbi.nlm.nih.gov/pubmed/33495597	Hypertrophic cardiomyopathy
9	17:44296846	17	43688317	rs28768976	27/07/2021	33495597	www.ncbi.nlm.nih.gov/pubmed/33495597	Hypertrophic cardiomyopathy (sarcomere negative)
9	17:44296846	17	43688317	rs28768976	1/02/2018	29255261	www.ncbi.nlm.nih.gov/pubmed/29255261	Neuroticism
9	17:44118848	17	43714850	rs2942168	20/04/2015	21292315	www.ncbi.nlm.nih.gov/pubmed/21292315	Parkinson's disease
9	17:44118848	17	44332351	rs2950015	18/09/2020	32888494	www.ncbi.nlm.nih.gov/pubmed/32888494	Hematocrit
9	17:44118848	17	44332351	rs2950015	18/09/2020	32888494	www.ncbi.nlm.nih.gov/pubmed/32888494	Hemoglobin
9	17:44118848	17	44343004	rs2950706	5/08/2019	31168069	www.ncbi.nlm.nih.gov/pubmed/31168069	Mood instability
9	17:44118848	17	44368212	rs2957297	17/09/2018	29844566	www.ncbi.nlm.nih.gov/pubmed/29844566	General cognitive ability
9	17:44118848	17	44331214	rs2957316	14/12/2021	34594039	www.ncbi.nlm.nih.gov/pubmed/34594039	Interstitial lung disease
9	17:44118848	17	44331214	rs2957316	14/12/2021	34594039	www.ncbi.nlm.nih.gov/pubmed/34594039	Pulmonary fibrosis
9	17:43564222	17	43511435	rs34465449	24/09/2021	33349686	www.ncbi.nlm.nih.gov/pubmed/33349686	Neuroticism conditioned on cognitive performance (multi-trait conditioning and joint analysis)
9	17:43520118	17	43520675	rs35329349	14/12/2021	34594039	www.ncbi.nlm.nih.gov/pubmed/34594039	White blood cell count
9	17:43564222	17	43515927	rs35354512	24/09/2021	33349686	www.ncbi.nlm.nih.gov/pubmed/33349686	Neuroticism conditioned on highest math class (multi-trait conditioning and joint analysis)
9	17:43564222	17	43515927	rs35354512	24/09/2021	33349686	www.ncbi.nlm.nih.gov/pubmed/33349686	Neuroticism conditioned on self-rated math ability (multi-trait conditioning and joint analysis)
9	17:43556807	17	43499328	rs35519908	5/02/2020	31666681	www.ncbi.nlm.nih.gov/pubmed/31666681	White matter microstructure (mean diusivities)
9	17:44118848	17	44192590	rs35524223	16/06/2017	28166213	www.ncbi.nlm.nih.gov/pubmed/28166213	Lung function (FEV1)
9	17:44118848	17	44192590	rs35524223	16/06/2017	28166213	www.ncbi.nlm.nih.gov/pubmed/28166213	Lung function (FVC)
9	17:44118848	17	44248837	rs35643216	17/06/2020	32327693	www.ncbi.nlm.nih.gov/pubmed/32327693	Hemoglobin levels
9	17:44118848	17	44353414	rs36050210	31/05/2022	33875891	www.ncbi.nlm.nih.gov/pubmed/33875891	IDP dMRI TBSS L3 Anterior limb of internal capsule R
9	17:44296846	17	43705601	rs365825	11/05/2017	27182965	www.ncbi.nlm.nih.gov/pubmed/27182965	Parkinson's disease
9	17:44118848	17	44611782	rs369234167	31/05/2022	33875891	www.ncbi.nlm.nih.gov/pubmed/33875891	aseg global volume BrainSegNotVent
9	17:44118848	17	44086726	rs370558872	1/07/2022	35361970	www.ncbi.nlm.nih.gov/pubmed/35361970	Educational attainment

9	17:44118848	17	44355304	rs371894855	31/05/2022	33875891	www.ncbi.nlm.nih.gov/pubmed/33875891	IDP dMRI TBSS ICVF Posterior limb of internal capsule R
9	17:44183317	17	44183317	rs371996525	28/03/2022	33821002	www.ncbi.nlm.nih.gov/pubmed/33821002	Brain shape (segment 1)
9	17:43564222	17	43557054	rs375822897	18/10/2018	30048462	www.ncbi.nlm.nih.gov/pubmed/30048462	Heel bone mineral density
9	17:44118848	17	44057595	rs3785884	21/09/2020	32888493	www.ncbi.nlm.nih.gov/pubmed/32888493	Hematocrit
9	17:44118848	17	44057595	rs3785884	21/09/2020	32888493	www.ncbi.nlm.nih.gov/pubmed/32888493	Hemoglobin concentration
9	17:44118848	17	43686419	rs385199	31/05/2022	33875891	www.ncbi.nlm.nih.gov/pubmed/33875891	aparc-pial lh area TotalSurface
9	17:44118848	17	43686419	rs385199	15/04/2021	33568662	www.ncbi.nlm.nih.gov/pubmed/33568662	Daytime nap
9	17:44118848	17	43687268	rs391338	31/05/2022	33875891	www.ncbi.nlm.nih.gov/pubmed/33875891	IDP dMRI TBSS L3 Anterior limb of internal capsule R
9	17:44296846	17	43719143	rs393152	8/07/2016	26077951	www.ncbi.nlm.nih.gov/pubmed/26077951	Corticobasal degeneration
9	17:44296846	17	43719143	rs393152	10/12/2009	19915575	www.ncbi.nlm.nih.gov/pubmed/19915575	Parkinson's disease
9	17:44861003	17	44859144	rs415430	14/09/2011	21812969	www.ncbi.nlm.nih.gov/pubmed/21812969	Parkinson's disease
9	17:44118848	17	44021717	rs4327091	24/08/2022	35762941	www.ncbi.nlm.nih.gov/pubmed/35762941	Systolic blood pressure
9	17:44118848	17	43686420	rs440778	31/05/2022	33875891	www.ncbi.nlm.nih.gov/pubmed/33875891	aseg global volume BrainSeg
9	17:44118848	17	43686420	rs440778	31/05/2022	33875891	www.ncbi.nlm.nih.gov/pubmed/33875891	aseg global volume BrainSegNotVent
9	17:44118848	17	43686420	rs440778	31/05/2022	33875891	www.ncbi.nlm.nih.gov/pubmed/33875891	aseg global volume BrainSegNotVentSurf
9	17:44118848	17	43686420	rs440778	31/05/2022	33875891	www.ncbi.nlm.nih.gov/pubmed/33875891	aseg global volume SupraTentorial
9	17:44118848	17	43686420	rs440778	31/05/2022	33875891	www.ncbi.nlm.nih.gov/pubmed/33875891	aseg global volume SupraTentorialNotVent
9	17:44118848	17	43686420	rs440778	31/05/2022	33875891	www.ncbi.nlm.nih.gov/pubmed/33875891	aseg lh volume CerebralWhiteMatter
9	17:44118848	17	43686420	rs440778	31/05/2022	33875891	www.ncbi.nlm.nih.gov/pubmed/33875891	aseg rh volume CerebralWhiteMatter
9	17:44118848	17	43686420	rs440778	31/05/2022	33875891	www.ncbi.nlm.nih.gov/pubmed/33875891	IDP T1 SIENAX brain-normalised volume
9	17:44118848	17	43686420	rs440778	31/05/2022	33875891	www.ncbi.nlm.nih.gov/pubmed/33875891	IDP T1 SIENAX brain-unnormalised volume
9	17:44118848	17	44205690	rs4471723	22/01/2019	29500382	www.ncbi.nlm.nih.gov/pubmed/29500382	Feeling guilty
9	17:44118848	17	44205690	rs4471723	4/08/2022	35505052	www.ncbi.nlm.nih.gov/pubmed/35505052	Whole brain restricted isotropic diffusion (multivariate analysis)
9	17:44118848	17	44192618	rs4606752	30/08/2017	27863252	www.ncbi.nlm.nih.gov/pubmed/27863252	High light scatter reticulocyte count
9	17:44118848	17	44192618	rs4606752	30/08/2017	27863252	www.ncbi.nlm.nih.gov/pubmed/27863252	Reticulocyte count
9	17:43556807	17	43471489	rs4763	30/11/2017	29059683	www.ncbi.nlm.nih.gov/pubmed/29059683	Breast cancer

9	17:43556807	17	43471489	rs4763	5/02/2020	31666681	www.ncbi.nlm.nih.gov/pubmed/31666681	White matter microstructure (axial diusivities)
9	17:44118848	17	44787313	rs538628	4/05/2021	32963231	www.ncbi.nlm.nih.gov/pubmed/32963231	Cortical surface area
9	17:44118848	17	44787313	rs538628	22/01/2019	29500382	www.ncbi.nlm.nih.gov/pubmed/29500382	Feeling nervous
9	17:44118848	17	44787313	rs538628	3/12/2018	28196072	www.ncbi.nlm.nih.gov/pubmed/28196072	Male-pattern baldness
9	17:44118848	17	44284057	rs548785130	5/02/2020	31666681	www.ncbi.nlm.nih.gov/pubmed/31666681	White matter microstructure (radial diusivities)
9	17:44118848	17	44338694	rs550530305	5/02/2020	31666681	www.ncbi.nlm.nih.gov/pubmed/31666681	White matter microstructure (axial diusivities)
9	17:44118848	17	44324572	rs555203107	17/12/2018	28957414	www.ncbi.nlm.nih.gov/pubmed/28957414	Red cell distribution width
9	17:44118848	17	43844560	rs55657917	16/08/2018	29899525	www.ncbi.nlm.nih.gov/pubmed/29899525	Accelerometer-based physical activity measurement (average acceleration)
9	17:44118848	17	43844560	rs55657917	22/01/2019	29500382	www.ncbi.nlm.nih.gov/pubmed/29500382	Experiencing mood swings
9	17:44118848	17	43844560	rs55657917	22/01/2019	29500382	www.ncbi.nlm.nih.gov/pubmed/29500382	Feeling hurt
9	17:44118848	17	43844560	rs55657917	18/01/2019	30531941	www.ncbi.nlm.nih.gov/pubmed/30531941	Physical activity (overall physical activity time)
9	17:44118848	17	44164323	rs55692232	14/09/2020	32451486	www.ncbi.nlm.nih.gov/pubmed/32451486	Alcohol consumption (drinks per week) (MTAG)
9	17:44118848	17	44517766	rs557236423	31/05/2022	33875891	www.ncbi.nlm.nih.gov/pubmed/33875891	aseg global volume BrainSeg
9	17:44118848	17	44034575	rs55746658	10/03/2022	34732054	www.ncbi.nlm.nih.gov/pubmed/34732054	High-sensitivity cardiac troponin I concentration (MTAG)
9	17:44118848	17	44034575	rs55746658	10/03/2022	34732054	www.ncbi.nlm.nih.gov/pubmed/34732054	High-sensitivity cardiac troponin T levels (MTAG)
9	17:44118848	17	43679193	rs55826010	31/05/2022	33875891	www.ncbi.nlm.nih.gov/pubmed/33875891	aparc-a2009s lh thickness S-orbital-H-Shaped
9	17:44118848	17	43757450	rs55974014	9/01/2021	32989287	www.ncbi.nlm.nih.gov/pubmed/32989287	Left-handedness
9	17:44118848	17	44013966	rs56072903	4/08/2022	35585065	www.ncbi.nlm.nih.gov/pubmed/35585065	F-small fish liking (derived food-liking factor)
9	17:44118848	17	44013966	rs56072903	4/08/2022	35585065	www.ncbi.nlm.nih.gov/pubmed/35585065	Herring liking
9	17:44118848	17	44013966	rs56072903	4/08/2022	35585065	www.ncbi.nlm.nih.gov/pubmed/35585065	Mackerel liking
9	17:44118848	17	44077851	rs56108300	21/09/2020	32888493	www.ncbi.nlm.nih.gov/pubmed/32888493	Neutrophil count
9	17:44118848	17	43512318	rs56168933	20/07/2020	32198502	www.ncbi.nlm.nih.gov/pubmed/32198502	Cortical surface area (global PC1)
9	17:43564222	17	43555253	rs56192752	19/10/2018	30038396	www.ncbi.nlm.nih.gov/pubmed/30038396	Educational attainment (years of education)

9	17:43564222	17	43555253	rs56192752	24/09/2021	33349686	www.ncbi.nlm.nih.gov/pubmed/33349686	Neuroticism conditioned on average household income before tax (multi-trait conditioning and joint analysis)
9	17:43564222	17	43555253	rs56192752	24/09/2021	33349686	www.ncbi.nlm.nih.gov/pubmed/33349686	Neuroticism conditioned on educational attainment (multi-trait conditioning and joint analysis)
9	17:43564222	17	43555253	rs56192752	24/09/2021	33349686	www.ncbi.nlm.nih.gov/pubmed/33349686	Neuroticism conditioned on Townsend deprivation index (multi-trait conditioning and joint analysis)
9	17:44118848	17	44041107	rs56280951	22/01/2019	29500382	www.ncbi.nlm.nih.gov/pubmed/29500382	Feeling miserable
9	17:44118848	17	43853922	rs56303031	8/02/2019	30595370	www.ncbi.nlm.nih.gov/pubmed/30595370	Heel bone mineral density
9	17:44118848	17	43871982	rs56319902	19/10/2018	30038396	www.ncbi.nlm.nih.gov/pubmed/30038396	Educational attainment (years of education)
9	17:44118848	17	43871982	rs56319902	7/03/2022	34910505	www.ncbi.nlm.nih.gov/pubmed/34910505	Vertex-wise sulcal depth
9	17:44118848	17	43871982	rs56319902	7/03/2022	34910505	www.ncbi.nlm.nih.gov/pubmed/34910505	Vertex-wise sulcal depth
9	17:44118848	17	43871982	rs56319902	7/03/2022	34910505	www.ncbi.nlm.nih.gov/pubmed/34910505	Vertex-wise sulcal depth
9	17:44118848	17	44326712	rs56368550	4/08/2022	35585065	www.ncbi.nlm.nih.gov/pubmed/35585065	Salad dressing liking
9	17:43803189	17	43798787	rs564681262	5/02/2020	31666681	www.ncbi.nlm.nih.gov/pubmed/31666681	White matter microstructure (axial diusivities)
9	17:44118848	17	43668850	rs564850730	4/08/2022	35585065	www.ncbi.nlm.nih.gov/pubmed/35585065	Shellfish liking
9	17:44118848	17	44364335	rs578102530	30/08/2022	32193382	www.ncbi.nlm.nih.gov/pubmed/32193382	Alcohol consumption (drinks per month) (UKB data field 1578, 4424)
9	17:43850645	17	43850645	rs60814418	1/07/2022	35361970	www.ncbi.nlm.nih.gov/pubmed/35361970	Educational attainment
9	17:44118848	17	43785349	rs61667602	14/09/2022	35922517	www.ncbi.nlm.nih.gov/pubmed/35922517	COVID-19 (critical illness vs population)
9	17:44118848	17	43785349	rs61667602	14/09/2022	35922517	www.ncbi.nlm.nih.gov/pubmed/35922517	COVID-19 (hospitalized vs population)
9	17:44118848	17	43785349	rs61667602	18/09/2019	31151762	www.ncbi.nlm.nih.gov/pubmed/31151762	Maximum habitual alcohol consumption
9	17:44118848	17	43744203	rs62053943	24/08/2021	34021172	www.ncbi.nlm.nih.gov/pubmed/34021172	A body shape index
9	17:44118848	17	43744203	rs62053943	2/05/2019	30867560	www.ncbi.nlm.nih.gov/pubmed/30867560	General factor of neuroticism
9	17:44118848	17	43744203	rs62053943	27/01/2020	31660654	www.ncbi.nlm.nih.gov/pubmed/31660654	Parkinson's disease

9	17:44118848	17	43744203	rs62053943	19/12/2019	31701892	www.ncbi.nlm.nih.gov/pubmed/31701892	Parkinson's disease or first degree relation to individual with Parkinson's disease
9	17:44118848	17	43744203	rs62053943	5/02/2020	31666681	www.ncbi.nlm.nih.gov/pubmed/31666681	White matter microstructure (radial diusivities)
9	17:44118848	17	43950441	rs62055489	15/12/2021	34560273	www.ncbi.nlm.nih.gov/pubmed/34560273	Cortical surface area
9	17:44118848	17	43964539	rs62055544	22/01/2019	29500382	www.ncbi.nlm.nih.gov/pubmed/29500382	Feeling fed-up
9	17:44118848	17	43964567	rs62055546	18/03/2019	30643258	www.ncbi.nlm.nih.gov/pubmed/30643258	Alcohol consumption (drinks per week)
9	17:44118848	17	43754850	rs62055691	4/10/2019	30679032	www.ncbi.nlm.nih.gov/pubmed/30679032	Alcohol consumption (drinks per week)
9	17:44118848	17	43758125	rs62055696	1/02/2022	34753499	www.ncbi.nlm.nih.gov/pubmed/34753499	Physical activity
9	17:44118848	17	43758125	rs62055696	1/02/2022	34753499	www.ncbi.nlm.nih.gov/pubmed/34753499	Physical activity
9	17:44118848	17	43758787	rs62055701	22/01/2019	29500382	www.ncbi.nlm.nih.gov/pubmed/29500382	Irritable mood
9	17:44118848	17	43838720	rs62055888	14/12/2021	34594039	www.ncbi.nlm.nih.gov/pubmed/34594039	Hematocrit
9	17:44118848	17	43848750	rs62055935	22/01/2019	29500382	www.ncbi.nlm.nih.gov/pubmed/29500382	Feeling nervous
9	17:44118848	17	43975415	rs62056789	27/05/2022	35181757	www.ncbi.nlm.nih.gov/pubmed/35181757	Generalized anxiety disorder (mental health questionnaire or predicted)
9	17:44118848	17	43975415	rs62056789	27/05/2022	35181757	www.ncbi.nlm.nih.gov/pubmed/35181757	Generalized anxiety disorder (phenotype risk score)
9	17:44118848	17	43975415	rs62056789	29/01/2020	31844048	www.ncbi.nlm.nih.gov/pubmed/31844048	Household income
9	17:44118848	17	43975415	rs62056789	29/01/2020	31844048	www.ncbi.nlm.nih.gov/pubmed/31844048	Household income (MTAG)
9	17:44118848	17	43975415	rs62056789	27/05/2022	35181757	www.ncbi.nlm.nih.gov/pubmed/35181757	Post-traumatic stress disorder (mental health questionnaire or predicted)
9	17:44118848	17	43975415	rs62056789	27/05/2022	35181757	www.ncbi.nlm.nih.gov/pubmed/35181757	Post-traumatic stress disorder (phenotype risk score)
9	17:44118848	17	43790005	rs62056934	7/03/2022	34910505	www.ncbi.nlm.nih.gov/pubmed/34910505	Vertex-wise cortical surface area
9	17:44118848	17	43790005	rs62056934	7/03/2022	34910505	www.ncbi.nlm.nih.gov/pubmed/34910505	Vertex-wise cortical surface area
9	17:44118848	17	43790005	rs62056934	7/03/2022	34910505	www.ncbi.nlm.nih.gov/pubmed/34910505	Vertex-wise cortical thickness
9	17:44118848	17	43856639	rs62057061	31/10/2018	29942085	www.ncbi.nlm.nih.gov/pubmed/29942085	Depressed affect
9	17:44118848	17	43859640	rs62057070	21/07/2020	32193296	www.ncbi.nlm.nih.gov/pubmed/32193296	Cortical surface area
9	17:44118848	17	43896032	rs62057107	12/11/2018	30038396	www.ncbi.nlm.nih.gov/pubmed/30038396	Educational attainment (MTAG)
9	17:44118848	17	43896032	rs62057107	12/11/2018	30038396	www.ncbi.nlm.nih.gov/pubmed/30038396	Highest math class taken (MTAG)

9	17:44118848	17	43903842	rs62057151	22/01/2019	29500382	www.ncbi.nlm.nih.gov/pubmed/29500382	Feeling worry
9	17:44118848	17	44243870	rs62060891	15/12/2021	34560273	www.ncbi.nlm.nih.gov/pubmed/34560273	Cortical thickness
9	17:44118848	17	44273653	rs62060954	27/04/2022	35213538	www.ncbi.nlm.nih.gov/pubmed/35213538	Degree of unsaturation
9	17:44118848	17	44018399	rs62061733	8/02/2019	30595370	www.ncbi.nlm.nih.gov/pubmed/30595370	Eosinophil counts
9	17:44118848	17	44018399	rs62061733	22/01/2019	29500382	www.ncbi.nlm.nih.gov/pubmed/29500382	Feeling hurt
9	17:44118848	17	44018488	rs62061734	30/11/2017	29059683	www.ncbi.nlm.nih.gov/pubmed/29059683	Breast cancer
9	17:44118848	17	44018488	rs62061734	25/06/2019	30804565	www.ncbi.nlm.nih.gov/pubmed/30804565	Sleep duration
9	17:44118848	17	44096553	rs62062288	14/12/2018	30336701	www.ncbi.nlm.nih.gov/pubmed/30336701	Alcohol use disorder (consumption score)
9	17:44118848	17	44096553	rs62062288	7/09/2021	33861876	www.ncbi.nlm.nih.gov/pubmed/33861876	Alcohol use disorder (consumption score)
9	17:44118848	17	44096553	rs62062288	14/12/2018	30336701	www.ncbi.nlm.nih.gov/pubmed/30336701	Alcohol use disorder (total score)
9	17:44118848	17	44096553	rs62062288	18/03/2019	30643258	www.ncbi.nlm.nih.gov/pubmed/30643258	Automobile speeding propensity
9	17:44118848	17	44096553	rs62062288	22/01/2019	29500382	www.ncbi.nlm.nih.gov/pubmed/29500382	Feeling guilty
9	17:44118848	17	44096553	rs62062288	22/01/2019	29500382	www.ncbi.nlm.nih.gov/pubmed/29500382	Feeling worry
9	17:44118848	17	44096553	rs62062288	22/01/2019	29500382	www.ncbi.nlm.nih.gov/pubmed/29500382	Neurociticism
9	17:44118848	17	44096553	rs62062288	29/05/2020	32231276	www.ncbi.nlm.nih.gov/pubmed/32231276	Neuroticism
9	17:44118848	17	44096553	rs62062288	18/03/2019	30643258	www.ncbi.nlm.nih.gov/pubmed/30643258	Risk-taking tendency (4-domain principal component model)
9	17:44118848	17	44096553	rs62062288	22/01/2019	29500382	www.ncbi.nlm.nih.gov/pubmed/29500382	Worry too long after an embarrassing experience
9	17:44118848	17	44027366	rs62062797	5/02/2020	31666681	www.ncbi.nlm.nih.gov/pubmed/31666681	White matter microstructure (fractional anisotropy)
9	17:44118848	17	44038785	rs62063281	4/08/2022	35585065	www.ncbi.nlm.nih.gov/pubmed/35585065	F-strong vegetable liking (derived food-liking factor)
9	17:44118848	17	44038785	rs62063281	18/03/2019	30643258	www.ncbi.nlm.nih.gov/pubmed/30643258	Number of sexual partners
9	17:44118848	17	44038785	rs62063281	8/02/2019	30664745	www.ncbi.nlm.nih.gov/pubmed/30664745	Osteoarthritis (hip)
9	17:44118848	17	44038785	rs62063281	26/11/2020	32589924	www.ncbi.nlm.nih.gov/pubmed/32589924	Osteoarthrosis (time to event)
9	17:44118848	17	44038785	rs62063281	17/11/2022	36324656	www.ncbi.nlm.nih.gov/pubmed/36324656	Risk-taking behavior (multivariate analysis)
9	17:44118848	17	44061023	rs62063786	17/06/2020	32327693	www.ncbi.nlm.nih.gov/pubmed/32327693	Hemoglobin levels

9	17:44118848	17	43654468	rs62064364	31/05/2022	33875891	www.ncbi.nlm.nih.gov/pubmed/33875891	IDP dMRI TBSS L3 Anterior limb of internal capsule L
9	17:44118848	17	43654468	rs62064364	28/01/2019	30535121	www.ncbi.nlm.nih.gov/pubmed/30535121	Macular thickness
9	17:44118848	17	43654468	rs62064364	5/02/2020	31666681	www.ncbi.nlm.nih.gov/pubmed/31666681	White matter microstructure (axial diusivities)
9	17:44118848	17	43666001	rs62064395	31/05/2022	33875891	www.ncbi.nlm.nih.gov/pubmed/33875891	IDP dMRI ProbtrackX MD str l
9	17:44118848	17	43666001	rs62064395	31/05/2022	33875891	www.ncbi.nlm.nih.gov/pubmed/33875891	IDP dMRI TBSS L2 Genu of corpus callosum
9	17:44118848	17	43666001	rs62064395	31/05/2022	33875891	www.ncbi.nlm.nih.gov/pubmed/33875891	IDP dMRI TBSS L3 Genu of corpus callosum
9	17:44118848	17	43666001	rs62064395	31/05/2022	33875891	www.ncbi.nlm.nih.gov/pubmed/33875891	IDP dMRI TBSS MD Anterior corona radiata L
9	17:44118848	17	43473307	rs62064597	24/08/2022	35762941	www.ncbi.nlm.nih.gov/pubmed/35762941	Systolic blood pressure
9	17:43556807	17	43474668	rs62064598	2/05/2019	30867560	www.ncbi.nlm.nih.gov/pubmed/30867560	General factor of neuroticism
9	17:43556807	17	43488792	rs62064641	8/02/2019	30595370	www.ncbi.nlm.nih.gov/pubmed/30595370	White blood cell count
9	17:43556807	17	43488792	rs62064641	31/10/2018	29942085	www.ncbi.nlm.nih.gov/pubmed/29942085	Worry
9	17:44118848	17	44082528	rs62064667	31/05/2022	33875891	www.ncbi.nlm.nih.gov/pubmed/33875891	IDP dMRI TBSS L1 Anterior corona radiata L
9	17:44118848	17	43573061	rs62065452	31/05/2022	33875891	www.ncbi.nlm.nih.gov/pubmed/33875891	aparc-Desikan rh area lateraloccipital
9	17:44118848	17	43573061	rs62065452	31/05/2022	33875891	www.ncbi.nlm.nih.gov/pubmed/33875891	aparc-Desikan rh volume fusiform
9	17:44118848	17	43573061	rs62065452	31/05/2022	33875891	www.ncbi.nlm.nih.gov/pubmed/33875891	aparc-DKTatlas rh area lateraloccipital
9	17:44118848	17	43573419	rs62065453	22/01/2019	29500382	www.ncbi.nlm.nih.gov/pubmed/29500382	Feeling nervous
9	17:44118848	17	43573419	rs62065453	22/01/2019	29500382	www.ncbi.nlm.nih.gov/pubmed/29500382	Irritable mood
9	17:44118848	17	43573419	rs62065453	9/06/2021	33972514	www.ncbi.nlm.nih.gov/pubmed/33972514	Liver enzyme levels (alkaline phosphatase)
9	17:44118848	17	43573419	rs62065453	1/02/2018	29255261	www.ncbi.nlm.nih.gov/pubmed/29255261	Neuroticism
9	17:44118848	17	43688042	rs62066084	4/08/2022	35505052	www.ncbi.nlm.nih.gov/pubmed/35505052	Whole brain free water diffusion (multivariate analysis)
9	17:44118848	17	44308053	rs62070949	31/05/2022	33875891	www.ncbi.nlm.nih.gov/pubmed/33875891	aparc-Desikan rh area fusiform
9	17:44118848	17	44308053	rs62070949	31/05/2022	33875891	www.ncbi.nlm.nih.gov/pubmed/33875891	aparc-DKTatlas rh area fusiform
9	17:44118848	17	44308053	rs62070949	31/05/2022	33875891	www.ncbi.nlm.nih.gov/pubmed/33875891	aparc-pial rh area fusiform
9	17:44118848	17	44316491	rs62073099	4/08/2022	35585065	www.ncbi.nlm.nih.gov/pubmed/35585065	F-bitter food liking (derived food-liking factor)
9	17:44118848	17	44316491	rs62073099	4/08/2022	35585065	www.ncbi.nlm.nih.gov/pubmed/35585065	F-strong flavour liking (derived food-liking factor)

9	17:44118848	17	44388912	rs62073233	27/04/2022	35213538	www.ncbi.nlm.nih.gov/pubmed/35213538	Glucose levels
9	17:44118848	17	43696159	rs675600	30/08/2017	27863252	www.ncbi.nlm.nih.gov/pubmed/27863252	Granulocyte count
9	17:44118848	17	43696159	rs675600	30/08/2017	27863252	www.ncbi.nlm.nih.gov/pubmed/27863252	Myeloid white cell count
9	17:44118848	17	43696159	rs675600	30/08/2017	27863252	www.ncbi.nlm.nih.gov/pubmed/27863252	Neutrophil count
9	17:44118848	17	43696159	rs675600	30/08/2017	27863252	www.ncbi.nlm.nih.gov/pubmed/27863252	Sum basophil neutrophil counts
9	17:44118848	17	43696159	rs675600	30/08/2017	27863252	www.ncbi.nlm.nih.gov/pubmed/27863252	Sum neutrophil eosinophil counts
9	17:44118848	17	44860021	rs70600	22/01/2019	29500382	www.ncbi.nlm.nih.gov/pubmed/29500382	Irritable mood
9	17:44118848	17	44332793	rs71375338	18/09/2020	32888494	www.ncbi.nlm.nih.gov/pubmed/32888494	Neutrophil count
9	17:44118848	17	44332793	rs71375338	21/09/2020	32888493	www.ncbi.nlm.nih.gov/pubmed/32888493	Neutrophil count
9	17:44118848	17	44332793	rs71375338	13/12/2021	34469753	www.ncbi.nlm.nih.gov/pubmed/34469753	Neutrophil-to-lymphocyte ratio
9	17:43520118	17	43484598	rs73984391	31/05/2022	33875891	www.ncbi.nlm.nih.gov/pubmed/33875891	IDP dMRI TBSS L3 Anterior corona radiata L
9	17:44118848	17	44260845	rs74693982	31/05/2022	33875891	www.ncbi.nlm.nih.gov/pubmed/33875891	IDP dMRI ProbtrackX L1 slf l
9	17:44118848	17	43810873	rs75022332	22/01/2019	29500382	www.ncbi.nlm.nih.gov/pubmed/29500382	Worry too long after an embarrassing experience
9	17:44118848	17	44357654	rs75067210	4/08/2022	35585065	www.ncbi.nlm.nih.gov/pubmed/35585065	Beans liking
9	17:44118848	17	44357654	rs75067210	4/08/2022	35585065	www.ncbi.nlm.nih.gov/pubmed/35585065	Broad bean liking
9	17:44118848	17	43973233	rs76324150	28/05/2020	32231278	www.ncbi.nlm.nih.gov/pubmed/32231278	Refractive error
9	17:44118848	17	43758887	rs76539714	24/10/2022	34321204	www.ncbi.nlm.nih.gov/pubmed/34321204	Albumin levels
9	17:44118848	17	43758887	rs76539714	31/05/2022	33875891	www.ncbi.nlm.nih.gov/pubmed/33875891	aparc-Desikan lh area fusiform
9	17:44118848	17	43758887	rs76539714	31/05/2022	33875891	www.ncbi.nlm.nih.gov/pubmed/33875891	aparc-DKTatlas lh area fusiform
9	17:44118848	17	43758887	rs76539714	31/05/2022	33875891	www.ncbi.nlm.nih.gov/pubmed/33875891	IDP dMRI TBSS L1 External capsule R
9	17:44118848	17	44189858	rs76640332	30/08/2017	27863252	www.ncbi.nlm.nih.gov/pubmed/27863252	Lymphocyte percentage of white cells
9	17:44118848	17	43776741	rs76761706	22/01/2019	29500382	www.ncbi.nlm.nih.gov/pubmed/29500382	Neurociticism
9	17:44118848	17	43658409	rs769375	31/05/2022	33875891	www.ncbi.nlm.nih.gov/pubmed/33875891	IDP dMRI TBSS L3 Superior fronto-occipital fasciculus L
9	17:44118848	17	43810896	rs77804065	22/01/2019	29500382	www.ncbi.nlm.nih.gov/pubmed/29500382	Feeling guilty
9	17:44118848	17	43810896	rs77804065	1/03/2018	29397368	www.ncbi.nlm.nih.gov/pubmed/29397368	Headache
9	17:44118848	17	43810896	rs77804065	18/09/2019	31151762	www.ncbi.nlm.nih.gov/pubmed/31151762	Maximum habitual alcohol consumption
9	17:44118848	17	43810896	rs77804065	31/10/2018	29942085	www.ncbi.nlm.nih.gov/pubmed/29942085	Neuroticism

9	17:44118848	17	43810896	rs77804065	1/02/2018	29255261	www.ncbi.nlm.nih.gov/pubmed/29255261	Neuroticism
9	17:44118848	17	44051612	rs77875796	29/04/2022	34613391	www.ncbi.nlm.nih.gov/pubmed/34613391	Occupational attainment
9	17:44118848	17	44339607	rs77917260	31/05/2022	33875891	www.ncbi.nlm.nih.gov/pubmed/33875891	IDP dMRI TBSS L1 External capsule L
9	17:44118848	17	44290850	rs78826239	31/05/2022	33875891	www.ncbi.nlm.nih.gov/pubmed/33875891	aparc-a2009s rh area G-oc-temp-lat-fusifor
9	17:44118848	17	44189910	rs78929339	14/12/2021	34594039	www.ncbi.nlm.nih.gov/pubmed/34594039	Medication use (anilides)
9	17:44118848	17	43828764	rs79172804	31/05/2022	33875891	www.ncbi.nlm.nih.gov/pubmed/33875891	IDP dMRI TBSS ICVF Superior fronto-occipital fasciculus L
9	17:44118848	17	43828764	rs79172804	31/05/2022	33875891	www.ncbi.nlm.nih.gov/pubmed/33875891	IDP dMRI TBSS ICVF Superior fronto-occipital fasciculus R
9	17:44118848	17	43828764	rs79172804	31/05/2022	33875891	www.ncbi.nlm.nih.gov/pubmed/33875891	IDP dMRI TBSS L1 Superior fronto-occipital fasciculus R
9	17:44118848	17	43828764	rs79172804	31/05/2022	33875891	www.ncbi.nlm.nih.gov/pubmed/33875891	IDP dMRI TBSS L2 Superior fronto-occipital fasciculus L
9	17:44118848	17	43828764	rs79172804	31/05/2022	33875891	www.ncbi.nlm.nih.gov/pubmed/33875891	IDP dMRI TBSS MD Superior corona radiata L
9	17:44118848	17	43828764	rs79172804	31/05/2022	33875891	www.ncbi.nlm.nih.gov/pubmed/33875891	IDP dMRI TBSS MD Superior fronto-occipital fasciculus L
9	17:44118848	17	43828764	rs79172804	31/05/2022	33875891	www.ncbi.nlm.nih.gov/pubmed/33875891	IDP dMRI TBSS MD Superior fronto-occipital fasciculus R
9	17:44118848	17	44207932	rs79301522	22/01/2019	29500382	www.ncbi.nlm.nih.gov/pubmed/29500382	Neurociticism
9	17:44118848	17	43940021	rs79412431	27/03/2019	30804560	www.ncbi.nlm.nih.gov/pubmed/30804560	FEV1
9	17:44118848	17	43940021	rs79412431	27/03/2019	30804560	www.ncbi.nlm.nih.gov/pubmed/30804560	Lung function (FVC)
9	17:44118848	17	43940021	rs79412431	27/03/2019	30804560	www.ncbi.nlm.nih.gov/pubmed/30804560	Peak expiratory flow
9	17:44118848	17	43897722	rs79600142	21/07/2020	32193296	www.ncbi.nlm.nih.gov/pubmed/32193296	Cortical surface area
9	17:44118848	17	43463493	rs79724577	31/05/2022	33875891	www.ncbi.nlm.nih.gov/pubmed/33875891	IDP dMRI ProbtrackX MD atr l
9	17:44118848	17	43463493	rs79724577	31/05/2022	33875891	www.ncbi.nlm.nih.gov/pubmed/33875891	IDP dMRI TBSS ICVF Anterior corona radiata R
9	17:44118848	17	43463493	rs79724577	31/05/2022	33875891	www.ncbi.nlm.nih.gov/pubmed/33875891	IDP dMRI TBSS ICVF Retrolenticular part of internal capsule L
9	17:44118848	17	43849415	rs79730878	28/01/2019	30598549	www.ncbi.nlm.nih.gov/pubmed/30598549	Heel bone mineral density

9	17:44118848	17	43657257	rs79742625	20/05/2019	30837455	www.ncbi.nlm.nih.gov/pubmed/30837455	Mouth ulcers
9	17:44118848	17	44013475	rs79857651	22/01/2019	29500382	www.ncbi.nlm.nih.gov/pubmed/29500382	Experiencing mood swings
9	17:44118848	17	44161470	rs80028338	4/08/2022	35585065	www.ncbi.nlm.nih.gov/pubmed/35585065	F-savoury food liking (derived food-liking factor)
9	17:44118848	17	44161470	rs80028338	5/02/2020	31666681	www.ncbi.nlm.nih.gov/pubmed/31666681	White matter microstructure (fractional anisotropy)
9	17:44118848	17	44224940	rs80103986	2/02/2018	29313844	www.ncbi.nlm.nih.gov/pubmed/29313844	Hand grip strength
9	17:44118848	17	43776742	rs80143279	7/07/2020	31972866	www.ncbi.nlm.nih.gov/pubmed/31972866	Sensitivity to environmental stress and adversity
9	17:44118848	17	44081064	rs8070723	5/04/2022	34855049	www.ncbi.nlm.nih.gov/pubmed/34855049	Lifetime smoking
9	17:44118848	17	44081064	rs8070723	5/04/2022	34855049	www.ncbi.nlm.nih.gov/pubmed/34855049	Lifetime smoking (without educational attainment)
9	17:44118848	17	44081064	rs8070723	3/01/2011	21044948	www.ncbi.nlm.nih.gov/pubmed/21044948	Parkinson's disease
9	17:44118848	17	44081064	rs8070723	14/07/2011	21685912	www.ncbi.nlm.nih.gov/pubmed/21685912	Progressive supranuclear palsy
9	17:44118848	17	44081064	rs8070723	4/11/2019	30239722	www.ncbi.nlm.nih.gov/pubmed/30239722	Waist-hip ratio
9	17:44118848	17	43893716	rs8072451	3/11/2015	25607358	www.ncbi.nlm.nih.gov/pubmed/25607358	Subcortical brain region volumes
9	17:44091886	17	44162597	rs8080583	12/01/2018	29186694	www.ncbi.nlm.nih.gov/pubmed/29186694	Cognitive ability
9	17:44118848	17	44065740	rs919462	12/01/2018	29146897	www.ncbi.nlm.nih.gov/pubmed/29146897	Male-pattern baldness
9	17:44091886	17	44187257	rs9303525	1/06/2012	22504418	www.ncbi.nlm.nih.gov/pubmed/22504418	Intracranial volume
9	17:44118848	17	44150152	rs974295	29/01/2020	31844048	www.ncbi.nlm.nih.gov/pubmed/31844048	Household income
9	17:44826056	17	44826056	rs9896243	31/10/2018	29942085	www.ncbi.nlm.nih.gov/pubmed/29942085	Worry
9	17:44118848	17	43822772	rs9906974	18/09/2020	32888494	www.ncbi.nlm.nih.gov/pubmed/32888494	Eosinophil percentage of white cells
10	17:62017421	17	61996255	rs2005172	7/02/2020	31761296	www.ncbi.nlm.nih.gov/pubmed/31761296	Appendicular lean mass
10	17:62017421	17	61996255	rs2005172	4/11/2020	33097823	www.ncbi.nlm.nih.gov/pubmed/33097823	Appendicular lean mass
10	17:62017421	17	61996255	rs2005172	8/02/2019	30593698	www.ncbi.nlm.nih.gov/pubmed/30593698	Fat-free mass
10	17:62017421	17	61996255	rs2005172	8/02/2019	30593698	www.ncbi.nlm.nih.gov/pubmed/30593698	Fat-free mass
10	17:62017421	17	61996255	rs2005172	8/02/2019	30593698	www.ncbi.nlm.nih.gov/pubmed/30593698	Fat-free mass

10	17:62017421	17	61996255	rs2005172	14/12/2021	34594039	www.ncbi.nlm.nih.gov/pubmed/34594039	Weight
10	17:62017421	17	62007498	rs2070776	24/09/2015	25282103	www.ncbi.nlm.nih.gov/pubmed/25282103	Height
10	17:62017421	17	62007498	rs2070776	24/10/2012	20881960	www.ncbi.nlm.nih.gov/pubmed/20881960	Height
10	17:62017421	17	62007498	rs2070776	2/07/2019	31217584	www.ncbi.nlm.nih.gov/pubmed/31217584	Height
10	17:62017421	17	62007498	rs2070776	5/08/2022	35831902	www.ncbi.nlm.nih.gov/pubmed/35831902	Height
10	17:62017421	17	62008318	rs2320125	5/08/2022	35831902	www.ncbi.nlm.nih.gov/pubmed/35831902	Height
10	17:62017421	17	62008318	rs2320125	22/09/2019	30575882	www.ncbi.nlm.nih.gov/pubmed/30575882	Waist-hip ratio
10	17:62017421	17	62008318	rs2320125	17/04/2020	31669095	www.ncbi.nlm.nih.gov/pubmed/31669095	Waist-to-hip ratio adjusted for BMI
10	17:62017421	17	62008318	rs2320125	4/11/2019	30239722	www.ncbi.nlm.nih.gov/pubmed/30239722	Waist-to-hip ratio adjusted for BMI
10	17:62017421	17	62008318	rs2320125	22/09/2019	30575882	www.ncbi.nlm.nih.gov/pubmed/30575882	Waist-to-hip ratio adjusted for BMI
10	17:62017421	17	62017421	rs2532111	17/07/2019	28552196	www.ncbi.nlm.nih.gov/pubmed/28552196	Height
10	17:62017421	17	62017421	rs2532111	24/08/2021	34021172	www.ncbi.nlm.nih.gov/pubmed/34021172	Hip circumference adjusted for BMI
10	17:62017421	17	62017421	rs2532111	8/02/2019	30595370	www.ncbi.nlm.nih.gov/pubmed/30595370	Waist-hip ratio
11	20:47627095	20	47648856	rs1022688	21/01/2019	29970889	www.ncbi.nlm.nih.gov/pubmed/29970889	Loneliness
11	20:47627095	20	47648856	rs1022688	21/01/2019	29970889	www.ncbi.nlm.nih.gov/pubmed/29970889	Loneliness (MTAG)
11	20:47746974	20	47817815	rs13036715	30/11/2018	30279531	www.ncbi.nlm.nih.gov/pubmed/30279531	Eudaimonic well-being
11	20:47746974	20	47849216	rs13038866	17/04/2020	31669095	www.ncbi.nlm.nih.gov/pubmed/31669095	Waist circumference adjusted for body mass index
11	20:47746974	20	47800829	rs13041213	23/02/2018	29326435	www.ncbi.nlm.nih.gov/pubmed/29326435	Intelligence (MTAG)
11	20:47627095	20	47705496	rs1538482	8/02/2019	30593698	www.ncbi.nlm.nih.gov/pubmed/30593698	Body fat percentage
11	20:47627095	20	47705496	rs1538482	8/02/2019	30593698	www.ncbi.nlm.nih.gov/pubmed/30593698	Body fat percentage
11	20:47627095	20	47705496	rs1538482	17/03/2017	27089181	www.ncbi.nlm.nih.gov/pubmed/27089181	Positive affect
11	20:47627095	20	47705496	rs1538482	24/09/2021	33349686	www.ncbi.nlm.nih.gov/pubmed/33349686	Subjective wellbeing conditioned on cognitive performance (multi-trait conditioning and joint analysis)
11	20:47627095	20	47603241	rs1569750	17/11/2022	34887591	www.ncbi.nlm.nih.gov/pubmed/34887591	Non-HDL cholesterol levels
11	20:47746974	20	47772264	rs17450430	24/09/2015	25282103	www.ncbi.nlm.nih.gov/pubmed/25282103	Height
11	20:47627095	20	47701765	rs1885163	17/11/2022	34887591	www.ncbi.nlm.nih.gov/pubmed/34887591	Non-HDL cholesterol levels

11	20:47746974	20	47552467	rs189331311	24/08/2021	34021172	www.ncbi.nlm.nih.gov/pubmed/34021172	Waist circumference adjusted for body mass index
11	20:47746974	20	47701024	rs2075677	18/03/2019	30643256	www.ncbi.nlm.nih.gov/pubmed/30643256	Positive affect
11	20:47746974	20	47701024	rs2075677	17/03/2017	27089181	www.ncbi.nlm.nih.gov/pubmed/27089181	Subjective well-being
11	20:47746974	20	47701024	rs2075677	7/07/2022	35589828	www.ncbi.nlm.nih.gov/pubmed/35589828	Subjective well-being
11	20:47627095	20	47582884	rs2295027	17/11/2022	34887591	www.ncbi.nlm.nih.gov/pubmed/34887591	Low density lipoprotein cholesterol levels
11	20:47627095	20	47582884	rs2295027	17/11/2022	34887591	www.ncbi.nlm.nih.gov/pubmed/34887591	Low density lipoprotein cholesterol levels
11	20:47627095	20	47582884	rs2295027	17/11/2022	34887591	www.ncbi.nlm.nih.gov/pubmed/34887591	Total cholesterol levels
11	20:47746974	20	47903019	rs237743	24/10/2012	20881960	www.ncbi.nlm.nih.gov/pubmed/20881960	Height
11	20:47746974	20	47851919	rs238150	7/07/2022	35589828	www.ncbi.nlm.nih.gov/pubmed/35589828	Subjective well-being
11	20:47746974	20	47851919	rs238150	7/07/2022	35589828	www.ncbi.nlm.nih.gov/pubmed/35589828	Subjective well-being
11	20:47746974	20	47844341	rs238164	16/10/2019	31562340	www.ncbi.nlm.nih.gov/pubmed/31562340	Height
11	20:47627095	20	47780105	rs2426139	15/12/2021	34560273	www.ncbi.nlm.nih.gov/pubmed/34560273	Cortical thickness
11	20:47495794	20	47493894	rs34116853	12/11/2018	30038396	www.ncbi.nlm.nih.gov/pubmed/30038396	Highest math class taken (MTAG)
11	20:47495794	20	47493894	rs34116853	12/11/2018	30038396	www.ncbi.nlm.nih.gov/pubmed/30038396	Self-reported math ability
11	20:47495794	20	47493894	rs34116853	12/11/2018	30038396	www.ncbi.nlm.nih.gov/pubmed/30038396	Self-reported math ability (MTAG)
11	20:47627095	20	47768988	rs348258	21/01/2020	31518406	www.ncbi.nlm.nih.gov/pubmed/31518406	Loneliness
11	20:47627095	20	47786190	rs348276	12/01/2018	29186694	www.ncbi.nlm.nih.gov/pubmed/29186694	Cognitive ability
11	20:47627095	20	47786190	rs348276	4/09/2019	31374203	www.ncbi.nlm.nih.gov/pubmed/31374203	Cognitive ability, years of educational attainment or schizophrenia (pleiotropy)
11	20:47627095	20	47786190	rs348276	24/09/2021	33349686	www.ncbi.nlm.nih.gov/pubmed/33349686	Subjective wellbeing conditioned on average household income before tax (multi-trait conditioning and joint analysis)
11	20:47627095	20	47786190	rs348276	24/09/2021	33349686	www.ncbi.nlm.nih.gov/pubmed/33349686	Subjective wellbeing conditioned on Townsend deprivation index (multi-trait conditioning and joint analysis)
11	20:47746974	20	47746974	rs34841991	30/11/2018	30279531	www.ncbi.nlm.nih.gov/pubmed/30279531	Hedonic well-being
11	20:47746974	20	47806092	rs3795075	14/12/2021	34594039	www.ncbi.nlm.nih.gov/pubmed/34594039	Height
11	20:47495794	20	47440220	rs4809728	18/09/2020	32888494	www.ncbi.nlm.nih.gov/pubmed/32888494	Mean reticulocyte volume

11	20:47495794	20	47495794	rs4810883	17/09/2018	29844566	www.ncbi.nlm.nih.gov/pubmed/29844566	General cognitive ability
11	20:47495794	20	47495794	rs4810883	21/09/2020	32888493	www.ncbi.nlm.nih.gov/pubmed/32888493	Red blood cell count
11	20:47495794	20	47495794	rs4810883	21/09/2020	32888493	www.ncbi.nlm.nih.gov/pubmed/32888493	Red blood cell count
11	20:47495794	20	47495794	rs4810883	18/09/2020	32888494	www.ncbi.nlm.nih.gov/pubmed/32888494	Red blood cell count
11	20:47495794	20	47477706	rs6012542	23/02/2018	29326435	www.ncbi.nlm.nih.gov/pubmed/29326435	Intelligence (MTAG)
11	20:47627095	20	47541517	rs6019535	5/09/2022	35764056	www.ncbi.nlm.nih.gov/pubmed/35764056	Attention deficit hyperactivity disorder or autism spectrum disorder or intelligence (pleiotropy)
11	20:47627095	20	47541517	rs6019535	12/09/2018	29942086	www.ncbi.nlm.nih.gov/pubmed/29942086	Intelligence
11	20:47627095	20	47541517	rs6019535	17/11/2022	34887591	www.ncbi.nlm.nih.gov/pubmed/34887591	Total cholesterol levels
11	20:47627095	20	47685815	rs6019624	17/09/2018	29844566	www.ncbi.nlm.nih.gov/pubmed/29844566	Reaction time
11	20:47627095	20	47666027	rs6090940	18/03/2019	30643256	www.ncbi.nlm.nih.gov/pubmed/30643256	Life satisfaction
11	20:47495794	20	47446556	rs6095319	26/10/2022	36150907	www.ncbi.nlm.nih.gov/pubmed/36150907	Common executive function
11	20:47627095	20	47532536	rs6095360	30/11/2018	30279531	www.ncbi.nlm.nih.gov/pubmed/30279531	Eudaimonic well-being
11	20:47627095	20	47532536	rs6095360	6/02/2019	29520040	www.ncbi.nlm.nih.gov/pubmed/29520040	Extremely high intelligence
11	20:47627095	20	47532536	rs6095360	23/02/2018	29326435	www.ncbi.nlm.nih.gov/pubmed/29326435	Intelligence (MTAG)
11	20:47627095	20	47627095	rs6095395	7/03/2022	34910505	www.ncbi.nlm.nih.gov/pubmed/34910505	Vertex-wise cortical thickness
11	20:47627095	20	47678201	rs6095417	17/09/2018	29844566	www.ncbi.nlm.nih.gov/pubmed/29844566	General cognitive ability
11	20:47495794	20	47481726	rs67021138	12/11/2018	30038396	www.ncbi.nlm.nih.gov/pubmed/30038396	Educational attainment (MTAG)
11	20:47627095	20	47652254	rs707533	24/09/2021	33349686	www.ncbi.nlm.nih.gov/pubmed/33349686	Subjective wellbeing conditioned on educational attainment (multi-trait conditioning and joint analysis)
11	20:47627095	20	47652254	rs707533	24/09/2021	33349686	www.ncbi.nlm.nih.gov/pubmed/33349686	Subjective wellbeing conditioned on highest math class (multi-trait conditioning and joint analysis)
11	20:47627095	20	47652254	rs707533	24/09/2021	33349686	www.ncbi.nlm.nih.gov/pubmed/33349686	Subjective wellbeing conditioned on self-rated math ability (multi-trait conditioning and joint analysis)
11	20:47746974	20	47701309	rs7264419	18/10/2022	34865855	www.ncbi.nlm.nih.gov/pubmed/34865855	Post-traumatic stress disorder (MTAG)
11	20:47495794	20	47447870	rs7267607	21/09/2020	32888493	www.ncbi.nlm.nih.gov/pubmed/32888493	Mean corpuscular hemoglobin
11	20:47746974	20	47762249	rs8122757	8/02/2019	30595370	www.ncbi.nlm.nih.gov/pubmed/30595370	Lung function (FVC)

Supplementary Table 4: The 197 mapped genes from the IC candidate SNPs

ensg	symbol	chr	start	end	strand	type	entrezID	HUGO
ENSG00000164163	*ABCE1*	4	146019084	146050331	1	protein_coding	6059	*ABCE1*
ENSG00000224505	*AC002117.1*	17	43227767	43238877	-1	antisense	-	-
ENSG00000236234	*AC091132.1*	17	43530210	43541431	1	antisense	-	-
ENSG00000264038	*AC091132.2*	17	43494179	43494272	-1	miRNA	-	-
ENSG00000181513	*ACBD4*	17	43209967	43221548	1	protein_coding	79777	*ACBD4*
ENSG00000212673	*AL136115.1*	1	32379174	32380745	1	protein_coding	-	-
ENSG00000164162	*ANAPC10*	4	145888264	146019693	-1	protein_coding	10393	*ANAPC10*
ENSG00000143412	*ANXA9*	1	150954493	150968110	1	protein_coding	8416	*ANXA9*
ENSG00000124198	*ARFGEF2*	20	47538427	47653230	1	protein_coding	10564	*ARFGEF2*
ENSG00000159314	*ARHGAP27*	17	43471275	43511787	-1	protein_coding	201176	*ARHGAP27*
ENSG00000189079	*ARID2*	12	46123448	46301823	1	protein_coding	196528	*ARID2*
ENSG00000185829	*ARL17A*	17	44594068	44657088	-1	protein_coding	100506084	*ARL17A*
ENSG00000228696	*ARL17B*	17	44352150	44439130	-1	protein_coding	100506084	*ARL17B*
ENSG00000165997	*ARL5B*	10	18948334	18970568	1	protein_coding	221079	*ARL5B*
ENSG00000143437	*ARNT*	1	150782181	150849244	-1	protein_coding	405	*ARNT*
ENSG00000163141	*BNIPL*	1	151009046	151020076	1	protein_coding	149428	*BNIPL*
ENSG00000186665	*C17orf58*	17	65987217	65989765	-1	protein_coding	284018	*C17orf58*
ENSG00000224383	*C17orf72*	17	62075711	62081664	1	protein_coding	92340	*C17orf72*
ENSG00000119280	*C1orf198*	1	230972865	231005335	-1	protein_coding	84886	*C1orf198*
ENSG00000167131	*CCDC103*	17	42976510	42982758	1	protein_coding	388389	*CCDC103*
ENSG00000180329	*CCDC43*	17	42750437	42767147	-1	protein_coding	124808	*CCDC43*
ENSG00000108588	*CCDC47*	17	61822610	61853711	-1	protein_coding	57003	*CCDC47*
ENSG00000007312	*CD79B*	17	62006100	62009714	-1	protein_coding	974	*CD79B*
ENSG00000197622	*CDC42SE1*	1	151023447	151042801	-1	protein_coding	56882	*CDC42SE1*
ENSG00000258890	*CEP95*	17	62502706	62538579	1	protein_coding	90799	*CEP95*
ENSG00000143418	*CERS2*	1	150933059	150947479	-1	protein_coding	29956	*CERS2*

ENSG00000084636	*COL16A1*	1	32117848	32169920	-1	protein_coding	1307	*COL16A1*
ENSG00000126267	*COX6B1*	19	36139125	36149763	1	protein_coding	1340	*COX6B1*
ENSG00000120088	*CRHR1*	17	43699267	43913194	1	protein_coding	1394	*CRHR1*
ENSG00000204650	*CRHR1-IT1*	17	43697694	43725582	1	pseudogene	401884	*CRHR1-IT1*
ENSG00000124207	*CSE1L*	20	47662849	47713489	1	protein_coding	1434	*CSE1L*
ENSG00000213218	*CSH2*	17	61949372	61951126	-1	protein_coding	1443	*CSH2*
ENSG00000204414	*CSHL1*	17	61986957	61996198	-1	protein_coding	1444	*CSHL1*
ENSG00000267344	*CTB-39G8.3*	17	43474298	43474843	1	antisense	-	-
ENSG00000267121	*CTD-2020K17.1*	17	43268298	43299589	-1	antisense	339192	-
ENSG00000233483	*CTD-2020K17.4*	17	43322553	43326203	-1	antisense	101927036	-
ENSG00000253347	*CTD-2026D20.2*	17	45486688	45500892	-1	antisense	-	-
ENSG00000047230	*CTPS2*	23	16606126	16731059	-1	protein_coding	56474	*CTPS2*
ENSG00000143387	*CTSK*	1	150768684	150780799	-1	protein_coding	1513	*CTSK*
ENSG00000163131	*CTSS*	1	150702672	150738433	-1	protein_coding	1520	*CTSS*
ENSG00000161692	*DBF4B*	17	42785976	42829632	1	protein_coding	80174	*DBF4B*
ENSG00000163257	*DCAF16*	4	17802278	17812381	-1	protein_coding	54876	*DCAF16*
ENSG00000172992	*DCAKD*	17	43100708	43138473	-1	protein_coding	79877	*DCAKD*
ENSG00000124228	*DDX27*	20	47835884	47860614	1	protein_coding	55661	*DDX27*
ENSG00000198231	*DDX42*	17	61850963	61896677	1	protein_coding	11325	*DDX42*
ENSG00000108654	*DDX5*	17	62495734	62504317	-1	protein_coding	100616408	*DDX5*
ENSG00000137628	*DDX60*	4	169137444	169239958	-1	protein_coding	55601	*DDX60*
ENSG00000264070	*DND1P1*	17	43663237	43664295	1	pseudogene	-	*DND1P1*
ENSG00000178498	*DTX3*	12	57998405	58003587	1	protein_coding	196403	*DTX3*
ENSG00000121310	*ECHDC2*	1	53361656	53392884	-1	protein_coding	55268	*ECHDC2*
ENSG00000143369	*ECM1*	1	150480538	150486265	1	protein_coding	1893	*ECM1*
ENSG00000108883	*EFTUD2*	17	42927311	42977030	-1	protein_coding	9343	*EFTUD2*
ENSG00000143420	*ENSA*	1	150573327	150602088	-1	protein_coding	2029	*ENSA*

ENSG00000178607	*ERN1*	17	62116502	62208179	-1	protein_coding	2081	*ERN1*
ENSG00000214447	*FAM187A*	17	42977135	42982758	1	protein_coding	388389	*FAM187A*
ENSG00000232300	*FAM215B*	17	44636196	44640161	-1	sense_intronic	23591	*FAM215B*
ENSG00000143409	*FAM63A*	1	150969025	150980851	-1	protein_coding	55793	*FAM63A*
ENSG00000184922	*FMNL1*	17	43298811	43324687	1	protein_coding	752	*FMNL1*
ENSG00000183090	*FREM3*	4	144498455	144621828	-1	protein_coding	166752	*FREM3*
ENSG00000108592	*FTSJ3*	17	61896793	61907372	-1	protein_coding	117246	*FTSJ3*
ENSG00000180340	*FZD2*	17	42634925	42636907	1	protein_coding	2535	*FZD2*
ENSG00000143458	*GABPB2*	1	151043054	151098018	1	protein_coding	126626	*GABPB2*
ENSG00000259384	*GH1*	17	61994560	61996179	-1	protein_coding	2688	*GH1*
ENSG00000135423	*GLS2*	12	56864736	56882198	-1	protein_coding	27165	*GLS2*
ENSG00000143457	*GOLPH3L*	1	150618701	150669630	-1	protein_coding	55204	*GOLPH3L*
ENSG00000108433	*GOSR2*	17	45000483	45105003	1	protein_coding	9570	*GOSR2*
ENSG00000173264	*GPR137*	11	64037534	64056972	1	protein_coding	56834	*GPR137*
ENSG00000121764	*HCRTR1*	1	32083287	32098119	1	protein_coding	3061	*HCRTR1*
ENSG00000168517	*HEXIM2*	17	43238067	43247407	1	protein_coding	124790	*HEXIM2*
ENSG00000164161	*HHIP*	4	145567173	145666423	1	protein_coding	64399	*HHIP*
ENSG00000248890	*HHIP-AS1*	4	145564074	145582509	-1	antisense	646576	*HHIP-AS1*
ENSG00000197568	*HHLA3*	1	70820488	70851022	1	protein_coding	11147	*HHLA3*
ENSG00000108622	*ICAM2*	17	62079954	62097994	-1	protein_coding	3384	*ICAM2*
ENSG00000137965	*IFI44*	1	79115481	79129763	1	protein_coding	10561	*IFI44*
ENSG00000140968	*IRF8*	16	85932409	85956215	1	protein_coding	3394	*IRF8*
ENSG00000143543	*JTB*	1	153946745	153950164	-1	protein_coding	10899	*JTB*
ENSG00000120071	*KANSL1*	17	44107282	44302733	-1	protein_coding	101929776	*KANSL1*
ENSG00000214401	*KANSL1-AS1*	17	44270942	44274089	1	antisense	644246	*KANSL1-AS1*
ENSG00000158445	*KCNB1*	20	47980414	48099184	-1	protein_coding	3745	*KCNB1*
ENSG00000121774	*KHDRBS1*	1	32479430	32526451	1	protein_coding	10657	*KHDRBS1*
ENSG00000002549	*LAP3*	4	17578815	17609595	1	protein_coding	51056	*LAP3*
ENSG00000178177	*LCORL*	4	17842822	18023499	-1	protein_coding	254251	*LCORL*

ENSG00000108679	*LGALS3BP*	17	76967320	76976191	-1	protein_coding	3959	*LGALS3BP*
ENSG00000136490	*LIMD2*	17	61773262	61778532	-1	protein_coding	80774	*LIMD2*
ENSG00000228126	*LINC00568*	1	150488233	150490508	1	lincRNA	100874054	*LINC00568*
ENSG00000176681	*LRRC37A*	17	44370099	44415160	1	protein_coding	474170	*LRRC37A*
ENSG00000263142	*LRRC37A17P*	17	45055847	45131935	1	pseudogene	-	*LRRC37A17P*
ENSG00000238083	*LRRC37A2*	17	44588877	44633016	1	protein_coding	474170	*LRRC37A2*
ENSG00000176809	*LRRC37A3*	17	62850430	62915598	-1	protein_coding	101930430	*LRRC37A3*
ENSG00000214425	*LRRC37A4P*	17	43578685	43627701	-1	pseudogene	55073	*LRRC37A4P*
ENSG00000163155	*LYSMD1*	1	151132224	151138424	-1	protein_coding	388695	*LYSMD1*
ENSG00000006062	*MAP3K14*	17	43340488	43394414	-1	processed_transcript	-	*MAP3K14*
ENSG00000267278	*MAP3K14-AS1*	17	43325292	43345997	1	antisense	100133991	*MAP3K14-AS1*
ENSG00000198909	*MAP3K3*	17	61699775	61773663	1	protein_coding	4215	*MAP3K3*
ENSG00000186868	*MAPT*	17	43971748	44105700	1	protein_coding	4137	*MAPT*
ENSG00000264589	*MAPT-AS1*	17	43921017	43972966	-1	antisense	NA	*MAPT-AS1*
ENSG00000143384	*MCL1*	1	150547032	150552066	-1	protein_coding	4170	*MCL1*
ENSG00000151376	*ME3*	11	86152150	86383678	-1	protein_coding	10873	*ME3*
ENSG00000118579	*MED28*	4	17616254	17635728	1	protein_coding	80306	*MED28*
ENSG00000157601	*MX1*	21	42792231	42831141	1	protein_coding	4599	*MX1*
ENSG00000104177	*MYEF2*	15	48431625	48470714	-1	protein_coding	50804	*MYEF2*
ENSG00000136448	*NMT1*	17	43128978	43186384	1	protein_coding	4836	*NMT1*
ENSG00000073969	*NSF*	17	44668035	44834830	1	protein_coding	101930324	*NSF*
ENSG00000260075	*NSFP1*	17	44450221	44564507	1	pseudogene	728806	*NSFP1*
ENSG00000106268	*NUDT1*	7	2281857	2290781	1	protein_coding	4521	*NUDT1*
ENSG00000164164	*OTUD4*	4	146031990	146101313	-1	protein_coding	54726	*OTUD4*
ENSG00000041880	*PARP3*	3	51976361	51982883	1	protein_coding	10039	*PARP3*
ENSG00000162517	*PEF1*	1	32095463	32110497	-1	protein_coding	553115	*PEF1*
ENSG00000152556	*PFKM*	12	48498922	48540187	1	protein_coding	5213	*PFKM*
ENSG00000148985	*PGAP2*	11	3818954	3847601	1	protein_coding	27315	*PGAP2*

ENSG00000196155	*PLEKHG4*	16	67311413	67323402	1	protein_coding	25894	*PLEKHG4*
ENSG00000225190	*PLEKHM1*	17	43513266	43568115	-1	protein_coding	9842	*PLEKHM1*
ENSG00000256525	*POLG2*	17	62473902	62493154	-1	protein_coding	11232	*POLG2*
ENSG00000124126	*PREX1*	20	47240790	47444420	-1	protein_coding	57580	*PREX1*
ENSG00000117360	*PRPF3*	1	150293925	150325671	1	protein_coding	9129	*PRPF3*
ENSG00000143363	*PRUNE*	1	150980896	151008189	1	protein_coding	58497	*PRUNE*
ENSG00000087191	*PSMC5*	17	61904512	61909379	1	protein_coding	5705	*PSMC5*
ENSG00000159352	*PSMD4*	1	151227179	151239955	1	protein_coding	5710	*PSMD4*
ENSG00000184007	*PTP4A2*	1	32372022	32410457	-1	protein_coding	8073	*PTP4A2*
ENSG00000158079	*PTPDC1*	9	96793076	96872138	1	protein_coding	138639	*PTPDC1*
ENSG00000109113	*RAB34*	17	27041299	27045447	-1	protein_coding	100861437	*RAB34*
ENSG00000265315	*RN7SL199P*	17	44614900	44615180	1	misc_RNA	-	*RN7SL199P*
ENSG00000264584	*RN7SL600P*	1	150541449	150541745	-1	misc_RNA	-	*RN7SL600P*
ENSG00000265411	*RN7SL656P*	17	44397045	44397325	1	misc_RNA	-	*RN7SL656P*
ENSG00000264225	*RN7SL730P*	17	43547899	43548188	-1	misc_RNA	-	*RN7SL730P*
ENSG00000252698	*RNU7-101P*	17	44204762	44204817	1	snRNA	-	*RNU7-101P*
ENSG00000263715	*RP11-105N13.4*	17	43699274	43893909	1	processed_transcript	-	-
ENSG00000262633	*RP11-156P1.2*	17	45000499	45124520	1	protein_coding	9570	-
ENSG00000262879	*RP11-156P1.3*	17	45061411	45177689	-1	processed_transcript	101927060	-
ENSG00000261575	*RP11-259G18.1*	17	44344403	44346060	1	pseudogene	-	-
ENSG00000262500	*RP11-259G18.2*	17	44320972	44322410	1	pseudogene	-	-
ENSG00000262539	*RP11-259G18.3*	17	44336917	44337972	-1	pseudogene	-	-
ENSG00000203325	*RP11-277A4.4*	1	32517892	32539075	-1	antisense	-	-
ENSG00000265964	*RP11-293E1.1*	17	43810719	43812035	-1	lincRNA	-	-
ENSG00000265547	*RP11-293E1.2*	17	43809069	43810343	-1	lincRNA	-	-
ENSG00000231073	*RP11-316M1.3*	1	150945599	150948010	1	antisense	-	-
ENSG00000125695	*RP11-51F16.8*	17	61780205	61829457	-1	protein_coding	-	-

ENSG00000227431	*RP1-155G6.4*	20	47657000	47662581	-1	antisense	-	-
ENSG00000261886	*RP11-63A1.1*	17	44994136	45000400	-1	lincRNA	-	-
ENSG00000262881	*RP11-669E14.4*	17	43985036	43988145	-1	antisense	-	-
ENSG00000262372	*RP11-669E14.6*	17	44112679	44113136	1	antisense	-	-
ENSG00000261168	*RP11-68I18.10*	1	151102551	151104086	-1	sense_overlapping	-	-
ENSG00000263503	*RP11-707O23.5*	17	43678235	43679706	-1	pseudogene	-	-
ENSG00000235790	*RP11-73M7.6*	1	32109650	32125539	1	antisense	-	-
ENSG00000264078	*RP11-73M7.9*	1	32110295	32114972	1	antisense	-	-
ENSG00000266504	*RP11-798G7.4*	17	43631076	43636436	-1	pseudogene	-	-
ENSG00000131484	*RP11-798G7.5*	17	43580626	43612076	1	antisense	-	-
ENSG00000267198	*RP11-798G7.6*	17	43623170	43640596	1	lincRNA	101930029	-
ENSG00000267246	*RP11-798G7.7*	17	43627147	43636104	-1	processed_transcript	-	-
ENSG00000266918	*RP11-798G7.8*	17	43608943	43611204	1	lincRNA	-	-
ENSG00000254545	*RP11-84A19.3*	1	32254731	32256923	1	antisense	-	-
ENSG00000269967	*RP11-84A19.4*	1	32317514	32387442	-1	lincRNA	-	-
ENSG00000266497	*RP11-995C19.2*	17	44581312	44586705	1	pseudogene	-	-
ENSG00000246448	*RP13-578N3.3*	4	144621410	144786225	1	antisense	-	-
ENSG00000223382	*RP1-65J11.1*	1	31297075	31307131	1	lincRNA	-	-
ENSG00000231119	*RP4-569M23.2*	20	45981305	45983377	1	antisense	101927377	-
ENSG00000273451	*RP4-569M23.4*	20	45976881	45977886	-1	sense_intronic	-	-
ENSG00000163125	*RPRD2*	1	150335567	150449042	1	protein_coding	23248	*RPRD2*
ENSG00000179673	*RPRML*	17	45055523	45056614	-1	protein_coding	388394	*RPRML*
ENSG00000204652	*RPS26P8*	17	43685909	43686349	1	pseudogene	-	*RPS26P8*
ENSG00000213326	*RPS7P11*	17	44798948	44799533	-1	pseudogene	-	*RPS7P11*
ENSG00000007314	*SCN4A*	17	62015914	62050278	-1	protein_coding	6329	*SCN4A*
ENSG00000163156	*SCNM1*	1	151129140	151142773	1	protein_coding	100534012	*SCNM1*
ENSG00000143434	*SEMA6C*	1	151104161	151119104	-1	protein_coding	10500	*SEMA6C*

ENSG00000143379	*SETDB1*	1	150898739	150937213	1	protein_coding	9869	*SETDB1*
ENSG00000146409	*SLC18B1*	6	133090509	133119701	-1	protein_coding	116843	*SLC18B1*
ENSG00000151729	*SLC25A4*	4	186064395	186071536	1	protein_coding	291	*SLC25A4*
ENSG00000139514	*SLC7A1*	13	30083547	30169825	-1	protein_coding	6541	*SLC7A1*
ENSG00000108604	*SMARCD2*	17	61909444	61920425	-1	protein_coding	6603	*SMARCD2*
ENSG00000230758	*SNAP23P*	20	47654894	47655139	-1	pseudogene	-	*SNAP23P*
ENSG00000266402	*SNORA76*	17	62223330	62223836	1	lincRNA	677842	*SNORA76*
ENSG00000199753	*SNORD104*	17	62223443	62223512	1	snoRNA	692227	*SNORD104*
ENSG00000212304	*SNORD12*	20	47897220	47897309	1	snoRNA	692057	*SNORD12*
ENSG00000222365	*SNORD12B*	20	47896856	47896946	1	snoRNA	100113393	*SNORD12B*
ENSG00000209042	*SNORD12C*	20	47895477	47895565	1	snoRNA	26765	*SNORD12C*
ENSG00000184361	*SPATA32*	17	43331760	43339479	-1	protein_coding	124783	*SPATA32*
ENSG00000185294	*SPPL2C*	17	43922256	43924438	1	protein_coding	162540	*SPPL2C*
ENSG00000124214	*STAU1*	20	47729878	47804904	-1	protein_coding	6780	*STAU1*
ENSG00000256762	*STH*	17	44076616	44077060	1	protein_coding	246744	*STH*
ENSG00000266173	*STRADA*	17	61780192	61819330	-1	protein_coding	92335	*STRADA*
ENSG00000099365	*STX1B*	16	31000577	31021949	-1	protein_coding	112755	*STX1B*
ENSG00000136143	*SUCLA2*	13	48510622	48612125	-1	protein_coding	8803	*SUCLA2*
ENSG00000196562	*SULF2*	20	46285092	46415360	-1	protein_coding	55959	*SULF2*
ENSG00000143374	*TARS2*	1	150459887	150480078	1	protein_coding	102465529	*TARS2*
ENSG00000198933	*TBKBP1*	17	45771447	45789416	1	protein_coding	9755	*TBKBP1*
ENSG00000240280	*TCAM1P*	17	61926652	61941739	1	pseudogene	146771	*TCAM1P*
ENSG00000172465	*TCEAL1*	23	102883632	102885881	1	protein_coding	9338	*TCEAL1*
ENSG00000136478	*TEX2*	17	62224587	62340661	-1	protein_coding	55852	*TEX2*
ENSG00000121775	*TMEM39B*	1	32537632	32568467	1	protein_coding	55116	*TMEM39B*
ENSG00000163154	*TNFAIP8L2*	1	151129105	151132225	1	protein_coding	79626	*TNFAIP8L2*
ENSG00000137473	*TTC29*	4	147627790	147867034	-1	protein_coding	83894	*TTC29*
ENSG00000146530	*VWDE*	7	12370511	12443567	-1	protein_coding	221806	*VWDE*
ENSG00000108379	*WNT3*	17	44839872	44910520	-1	protein_coding	101929777	*WNT3*

ENSG00000177410	*ZFAS1*	20	47894715	47905797	1	antisense	441951	*ZFAS1*
ENSG00000101040	*ZMYND8*	20	45837859	45985567	-1	protein_coding	23613	*ZMYND8*
ENSG00000167377	*ZNF23*	16	71481500	71496998	-1	protein_coding	7571	*ZNF23*
ENSG00000124201	*ZNFX1*	20	47854483	47894963	-1	protein_coding	57169	*ZNFX1*

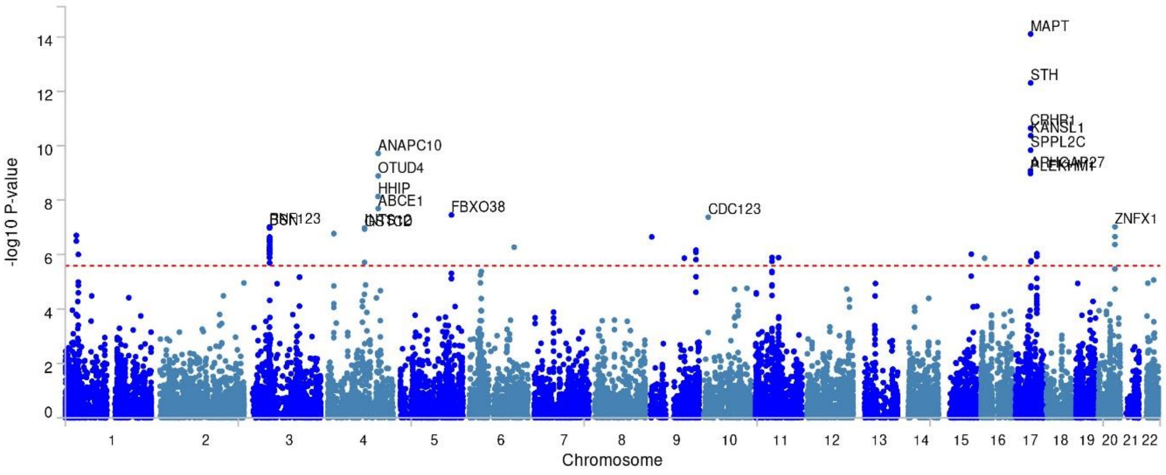

Supplementary Figure 2: Gene-based Manhattan plot

The plot displays genes associated with intrinsic capacity, for the gene mapped from whole SNPs included in the Meta GWAS (mapped using MAGMA gene mapping). The horizontal axis represents the chromosome numbers (1–22), while the vertical axis shows the significance level for the gene-based test (−log10(P-value)). The horizontal dashed line indicates the significance threshold, with genes above this line

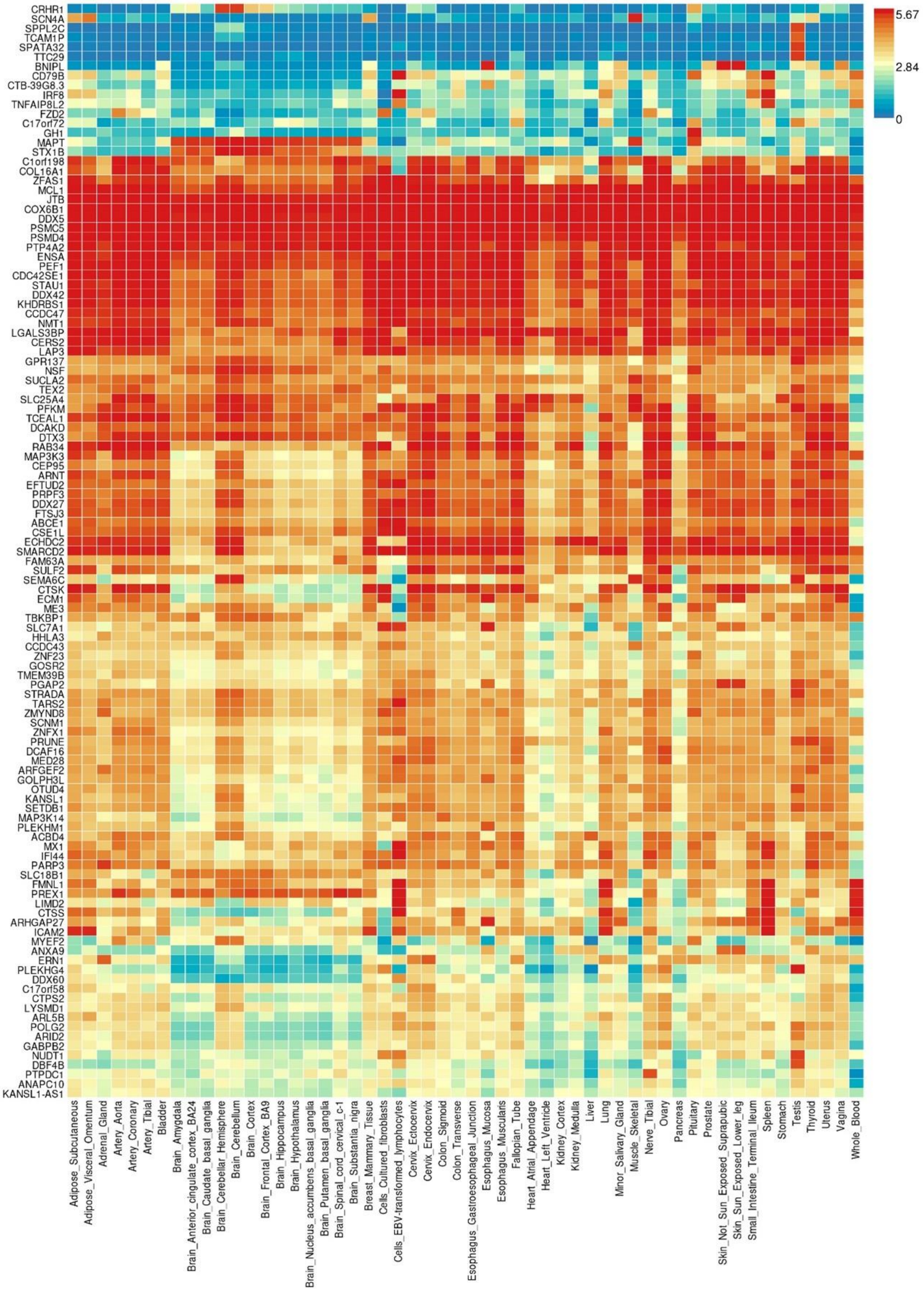

Supplementary Figure 3: Dot plot of beta coefficients and 95% confidence intervals for linear regression of IC on PGS deciles, adjusted for covariates.

Each point represents the estimated beta coefficient for a specific decile, while the vertical lines (whiskers) indicate the corresponding 95% confidence intervals.

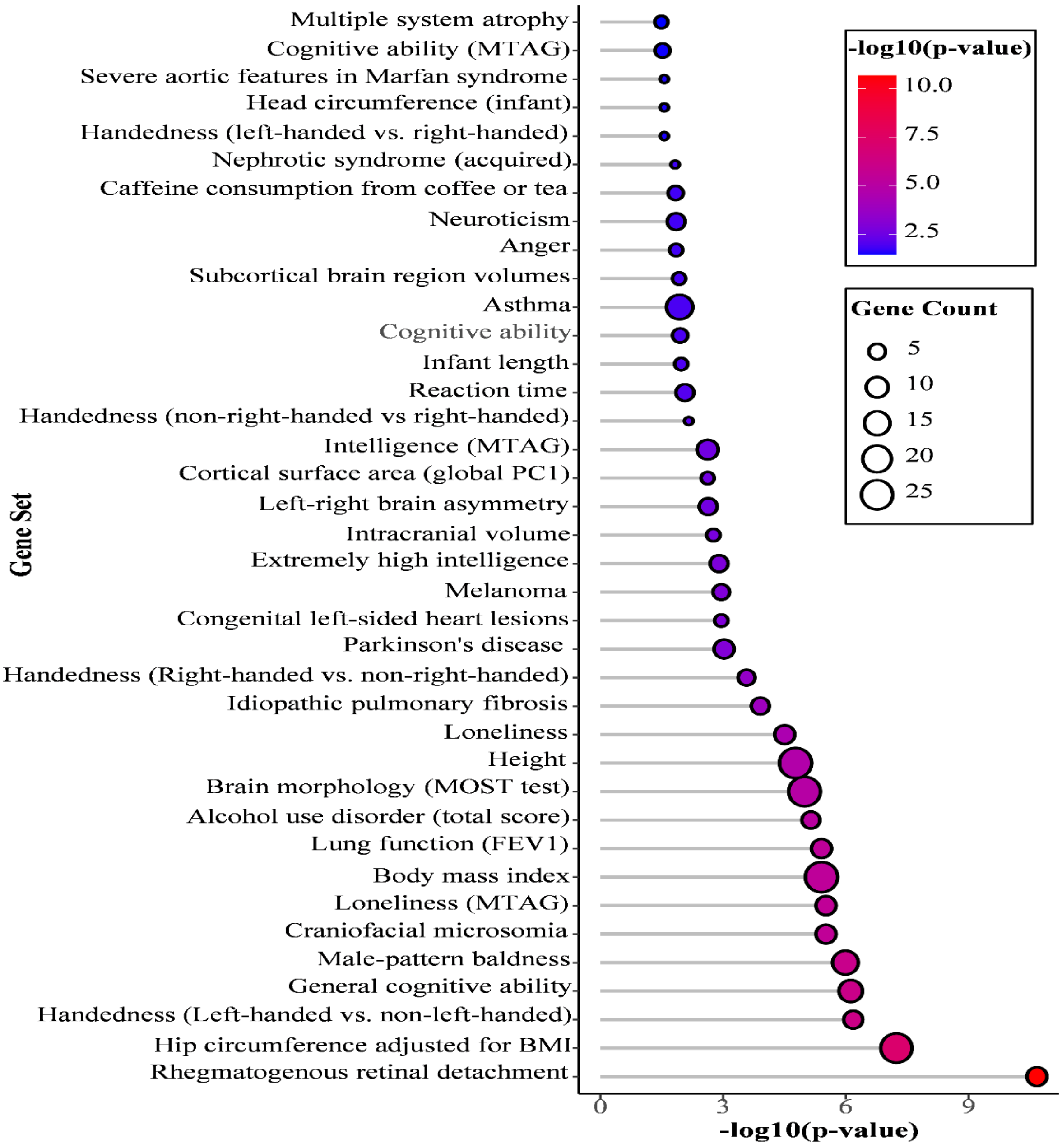

Supplementary Figure 4: Lollipop plot for gene set enrichment analysis

The figure shows the overlap of our 197 prioritized gene set with gene sets associated with different traits or diseases from the GWAS catalog. On the x-axis are the -log10 adjusted P-values, while the y-axis represents the gene sets ranked by level of significance, with stronger associations at the bottom. The length of the grey horizontal line indicates the -log10 adjusted P-value, with longer lines signifying greater enrichment significance. Circle sizes reflect the number of overlapping genes between our prioritized genes and the GWAS Catalog gene sets. MTAG=Multi Trait Analysis of GWAS, PC1 = principal componenent1, MOST=Multivariate Omnibus Statistical Test, FEV1 = Forced Expiratory Volume in 1 second, BMI = Body Mass Index.

Supplementary Table 6: Nominally Enriched pathways

Pathways	#Genes	#Genes enriched	expected	Fold Enrichment	+/-	raw P value	Pathway databse
Activation of APC/C and APC/C:Cdc20 mediated degradation of mitotic proteins	76	3	0.47	6.4	+	0.0116	Reactome
Activation, myristolyation of BID and translocation to mitochondria	4	1	0.02	40.51	+	0.0245	Reactome
Alzheimer disease-presenilin pathway	127	3	0.78	3.83	+	0.0441	Panther pathway
Antigen processing-Cross presentation	106	3	0.65	4.59	+	0.028	Reactome
APC/C:Cdc20 mediated degradation of mitotic proteins	75	3	0.46	6.48	+	0.0112	Reactome
APC/C:Cdc20 mediated degradation of Securin	67	3	0.41	7.26	+	0.00825	Reactome
APC/C:Cdh1 mediated degradation of Cdc20 and other APC/C:Cdh1 targeted proteins in late mitosis/early G1	73	3	0.45	6.66	+	0.0104	Reactome
APC/C-mediated degradation of cell cycle proteins	87	3	0.54	5.59	+	0.0167	Reactome
APC:Cdc20 mediated degradation of cell cycle proteins prior to satisfation of the cell cycle checkpoint	73	3	0.45	6.66	+	0.0104	Reactome
Apoptosis	173	4	1.07	3.75	+	0.0224	Reactome
Aryl hydrocarbon receptor signalling	7	1	0.04	23.15	+	0.0424	Reactome
Assembly of the pre-replicative complex	110	3	0.68	4.42	+	0.0307	Reactome
Asymmetric localization of PCP proteins	63	3	0.39	7.72	+	0.00696	Reactome
AUF1 (hnRNP D0) binds and destabilizes mRNA	53	2	0.33	6.11	+	0.0424	Reactome
Autodegradation of Cdh1 by Cdh1:APC/C	63	3	0.39	7.72	+	0.00696	Reactome
Autodegradation of the E3 ubiquitin ligase COP1	51	2	0.31	6.35	+	0.0396	Reactome
Cdc20:Phospho-APC/C mediated degradation of Cyclin A	72	3	0.44	6.75	+	0.01	Reactome
CDC42 GTPase cycle	154	4	0.95	4.21	+	0.0153	Reactome
CDK-mediated phosphorylation and removal of Cdc6	72	3	0.44	6.75	+	0.01	Reactome
Cell cycle	22	2	0.14	14.73	+	0.00805	Panther pathway
Cellular response to hypoxia	74	3	0.46	6.57	+	0.0108	Reactome
Citric acid cycle (TCA cycle)	22	2	0.14	14.73	+	0.00805	Reactome
Class B/2 (Secretin family receptors)	93	3	0.57	5.23	+	0.0199	Reactome
CLEC7A (Dectin-1) signaling	96	3	0.59	5.06	+	0.0216	Reactome
Coenzyme A biosynthesis	7	1	0.04	23.15	+	0.0424	Reactome
Coenzyme A biosynthesis	8	1	0.05	20.26	+	0.0483	Panther pathway

Cross-presentation of soluble exogenous antigens (endosomes)	49	2	0.3	6.61	+	0.0368	Reactome
C-type lectin receptors (CLRs)	138	4	0.85	4.7	+	0.0106	Reactome
Dectin-1 mediated noncanonical NF-kB signaling	59	3	0.36	8.24	+	0.0058	Reactome
Degradation of AXIN	54	2	0.33	6	+	0.0439	Reactome
Degradation of DVL	56	2	0.35	5.79	+	0.0469	Reactome
DNA Replication Pre-Initiation	127	3	0.78	3.83	+	0.0441	Reactome
FBXL7 down-regulates AURKA during mitotic entry and in early mitosis	54	2	0.33	6	+	0.0439	Reactome
GSK3B and BTRC:CUL1-mediated-degradation of NFE2L2	51	2	0.31	6.35	+	0.0396	Reactome
Hedgehog 'on' state	84	3	0.52	5.79	+	0.0152	Reactome
Hh mutants abrogate ligand secretion	58	2	0.36	5.59	+	0.0499	Reactome
Hh mutants are degraded by ERAD	55	2	0.34	5.89	+	0.0454	Reactome
Host Interactions of HIV factors	125	3	0.77	3.89	+	0.0424	Reactome
Interferon alpha/beta signaling	76	3	0.47	6.4	+	0.0116	Reactome
Interleukin-1 signaling	112	3	0.69	4.34	+	0.0322	Reactome
Intra-Golgi traffic	43	2	0.27	7.54	+	0.0289	Reactome
IRE1alpha activates chaperones	49	2	0.3	6.61	+	0.0368	Reactome
Ligand-receptor interactions	8	1	0.05	20.26	+	0.0483	Reactome
Metabolism of polyamines	57	2	0.35	5.69	+	0.0484	Reactome
mRNA Splicing	212	4	1.31	3.06	+	0.0425	Reactome
mRNA splicing	7	1	0.04	23.15	+	0.0424	Panther pathway
mRNA Splicing - Major Pathway	204	4	1.26	3.18	+	0.0378	Reactome
mRNA Splicing - Minor Pathway	50	2	0.31	6.48	+	0.0382	Reactome
Negative regulation of NOTCH4 signaling	52	2	0.32	6.23	+	0.041	Reactome
NIK-->noncanonical NF-kB signaling	58	3	0.36	8.38	+	0.00553	Reactome
Orexin and neuropeptides FF and QRFP bind to their respective receptors	8	1	0.05	20.26	+	0.0483	Reactome
p53-Independent DNA Damage Response	51	2	0.31	6.35	+	0.0396	Reactome
p53-Independent G1/S DNA damage checkpoint	51	2	0.31	6.35	+	0.0396	Reactome
PCP/CE pathway	91	3	0.56	5.34	+	0.0188	Reactome
Programmed Cell Death	205	4	1.27	3.16	+	0.0384	Reactome
PTK6 Regulates Proteins Involved in RNA Processing	5	1	0.03	32.41	+	0.0305	Reactome
Pyruvate metabolism and Citric Acid (TCA) cycle	54	2	0.33	6	+	0.0439	Reactome
RAC1 GTPase cycle	183	4	1.13	3.54	+	0.0269	Reactome
Regulation of activated PAK-2p34 by proteasome mediated degradation	49	2	0.3	6.61	+	0.0368	Reactome

Regulation of APC/C activators between G1/S and early anaphase	80	3	0.49	6.08	+	0.0134	Reactome
Regulation of Apoptosis	52	2	0.32	6.23	+	0.041	Reactome
Regulation of mitotic cell cycle	87	3	0.54	5.59	+	0.0167	Reactome
Regulation of ornithine decarboxylase (ODC)	50	2	0.31	6.48	+	0.0382	Reactome
Regulation of RUNX3 expression and activity	53	2	0.33	6.11	+	0.0424	Reactome
Replication of the SARS-CoV-1 genome	4	1	0.02	40.51	+	0.0245	Reactome
Replication of the SARS-CoV-2 genome	4	1	0.02	40.51	+	0.0245	Reactome
RMTs methylate histone arginines	48	2	0.3	6.75	+	0.0354	Reactome
RUNX1 interacts with co-factors whose precise effect on RUNX1 targets is not known	36	2	0.22	9	+	0.0208	Reactome
RUNX1 regulates transcription of genes involved in differentiation of keratinocytes	8	1	0.05	20.26	+	0.0483	Reactome
SARS-CoV-1 Genome Replication and Transcription	4	1	0.02	40.51	+	0.0245	Reactome
SARS-CoV-2 Genome Replication and Transcription	4	1	0.02	40.51	+	0.0245	Reactome
SCF-beta-TrCP mediated degradation of Emi1	54	2	0.33	6	+	0.0439	Reactome
Somitogenesis	54	2	0.33	6	+	0.0439	Reactome
Stabilization of p53	55	2	0.34	5.89	+	0.0454	Reactome
Switching of origins to a post-replicative state	91	3	0.56	5.34	+	0.0188	Reactome
Synaptic vesicle trafficking	30	2	0.19	10.8	+	0.0147	Panther pathway
Synthesis of DNA	120	3	0.74	4.05	+	0.0383	Reactome
TCF dependent signaling in response to WNT	200	4	1.23	3.24	+	0.0355	Reactome
TNFR2 non-canonical NF-kB pathway	98	3	0.6	4.96	+	0.0228	Reactome
Toxicity of botulinum toxin type C (botC)	3	1	0.02	54.02	+	0.0184	Reactome
Trafficking and processing of endosomal TLR	13	2	0.08	24.93	+	0.00282	Reactome
Transcriptional regulation by RUNX1	202	5	1.25	4.01	+	0.0084	Reactome
Ubiquitin Mediated Degradation of Phosphorylated Cdc25A	51	2	0.31	6.35	+	0.0396	Reactome
Ubiquitin proteasome pathway	58	2	0.36	5.59	+	0.0499	Panther pathway
Ubiquitin-dependent degradation of Cyclin D	51	2	0.31	6.35	+	0.0396	Reactome
Vif-mediated degradation of APOBEC3G	52	2	0.32	6.23	+	0.041	Reactome
Vpr-mediated induction of apoptosis by mitochondrial outer membrane permeabilization	3	1	0.02	54.02	+	0.0184	Reactome
Vpu mediated degradation of CD4	51	2	0.31	6.35	+	0.0396	Reactome

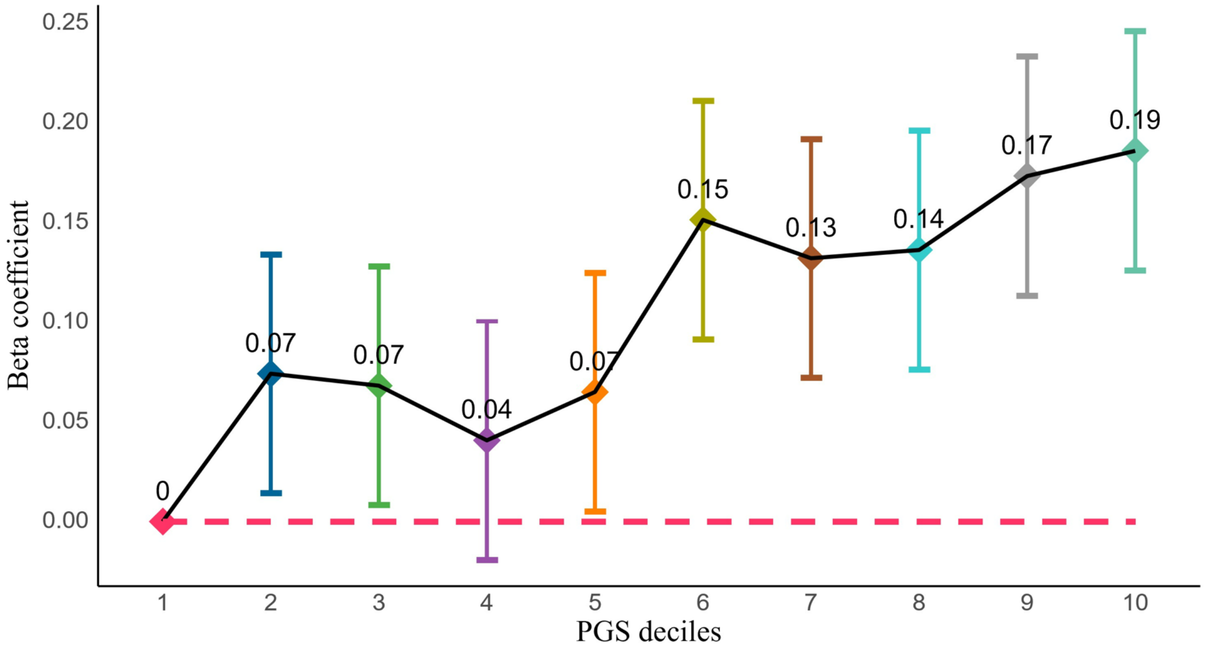

Supplementary Figure 5: Dot plot of beta coefficients and 95% confidence intervals for linear regression of IC on PGS deciles, adjusted for covariates.

Each point represents the estimated beta coefficient for a specific decile, while the vertical lines (whiskers) indicate the corresponding 95% confidence intervals.

Supplementary References

1. Forgetta V, Li R, Darmond-Zwaig C, Belisle A, Balion C, Roshandel D, et al. Cohort profile: genomic data for 26 622 individuals from the Canadian Longitudinal Study on Aging (CLSA). BMJ Open. 2022;12(3):e059021. doi: 10.1136/bmjopen-2021-059021.

2. Raina P, Wolfson C, Kirkland S, Griffith LE, Balion C, Cossette B, et al. Cohort Profile: The Canadian Longitudinal Study on Aging (CLSA). International Journal of Epidemiology. 2019;48(6):1752-3j. doi: 10.1093/ije/dyz173.

3. Beyene MB, Visvanathan R, Ahmed M, Benyamin B, Beard JR, Amare AT. Development and validation of an intrinsic capacity score in the UK Biobank study. Maturitas. 2024;185:107976. doi: https://doi.org/10.1016/j.maturitas.2024.107976.

4. Shrestha N. Factor analysis as a tool for survey analysis. American Journal of Applied Mathematics and Statistics. 2021;9(1):4-11.

5. Stellefson M, Hanik B. Strategies for Determining the Number of Factors to Retain in Exploratory Factor Analysis. Online Submission. 2008.

6. Horn JL. A rationale and test for the number of factors in factor analysis. Psychometrika. 1965;30:179-85.

7. Beaujean AA. Factor Analysis Using" R". Practical Assessment, Research & Evaluation. 2013;18(4):n4.

8. Rosseel Y. lavaan: An R package for structural equation modeling. Journal of statistical software. 2012;48:1-36.
